# Estimating epidemiologic dynamics from cross-sectional viral load distributions

**DOI:** 10.1126/science.abh0635

**Published:** 2021-06-03

**Authors:** James A. Hay, Lee Kennedy-Shaffer, Sanjat Kanjilal, Niall J. Lennon, Stacey B. Gabriel, Marc Lipsitch, Michael J. Mina

**Affiliations:** 1 Center for Communicable Disease Dynamics, Harvard T.H. Chan School of Public Health, Boston, MA, USA; 2 Department of Epidemiology, Harvard T.H. Chan School of Public Health, Boston, MA, USA; 3 Department of Immunology and Infectious Diseases, Harvard T.H. Chan School of Public Health, Boston, MA, USA; 4 Department of Mathematics and Statistics, Vassar College, Poughkeepsie, NY, USA; 5 Department of Population Medicine, Harvard Pilgrim Health Care Institute, Boston, MA, USA; 6 Department of Infectious Diseases, Brigham and Women’s Hospital, Boston, MA, USA; 7 Broad Institute of MIT and Harvard, Cambridge, MA, USA; 8 Department of Pathology, Brigham and Women’s Hospital, Boston, MA, USA

## Abstract

**INTRODUCTION:**

Current approaches to epidemic monitoring rely on case counts, test positivity rates, and reported deaths or hospitalizations. These metrics, however, provide a limited and often biased picture as a result of testing constraints, unrepresentative sampling, and reporting delays. Random cross-sectional virologic surveys can overcome some of these biases by providing snapshots of infection prevalence but currently offer little information on the epidemic trajectory without sampling across multiple time points.

**RATIONALE:**

We develop a new method that uses information inherent in cycle threshold (Ct) values from reverse transcription quantitative polymerase chain reaction (RT-qPCR) tests to robustly estimate the epidemic trajectory from multiple or even a single cross section of positive samples. Ct values are related to viral loads, which depend on the time since infection; Ct values are generally lower when the time between infection and sample collection is short. Despite variation across individuals, samples, and testing platforms, Ct values provide a probabilistic measure of time since infection. We find that the distribution of Ct values across positive specimens at a single time point reflects the epidemic trajectory: A growing epidemic will necessarily have a high proportion of recently infected individuals with high viral loads, whereas a declining epidemic will have more individuals with older infections and thus lower viral loads. Because of these changing proportions, the epidemic trajectory or growth rate should be inferable from the distribution of Ct values collected in a single cross section, and multiple successive cross sections should enable identification of the longer-term incidence curve. Moreover, understanding the relationship between sample viral loads and epidemic dynamics provides additional insights into why viral loads from surveillance testing may appear higher for emerging viruses or variants and lower for out-breaks that are slowing, even absent changes in individual-level viral kinetics.

**RESULTS:**

Using a mathematical model for population-level viral load distributions calibrated to known features of the severe acute respiratory syndrome coronavirus 2 (SARS-CoV-2) viral load kinetics, we show that the median and skewness of Ct values in a random sample change over the course of an epidemic. By formalizing this relationship, we demonstrate that Ct values from a single random cross section of virologic testing can estimate the time-varying reproductive number of the virus in a population, which we validate using data collected from comprehensive SARS-CoV-2 testing in long-term care facilities. Using a more flexible approach to modeling infection incidence, we also develop a method that can reliably estimate the epidemic trajectory in even more-complex populations, where interventions may be implemented and relaxed over time. This method performed well in estimating the epidemic trajectory in the state of Massachusetts using routine hospital admissions RT-qPCR testing data—accurately replicating estimates from other sources for the entire state.

**CONCLUSION:**

This work provides a new method for estimating the epidemic growth rate and a framework for robust epidemic monitoring using RT-qPCR Ct values that are often simply discarded. By deploying single or repeated (but small) random surveillance samples and making the best use of the semiquantitative testing data, we can estimate epidemic trajectories in real time and avoid biases arising from nonrandom samples or changes in testing practices over time. Understanding the relationship between population-level viral loads and the state of an epidemic reveals important implications and opportunities for interpreting virologic surveillance data. It also highlights the need for such surveillance, as these results show how to use it most informatively.

## Abstract (summary)

Estimating an epidemic’s trajectory is crucial for developing public health responses to infectious diseases, but case data used for such estimation are confounded by variable testing practices. We show that the population distribution of viral loads observed under random or symptom-based surveillance—in the form of cycle threshold (Ct) values obtained from reverse transcription quantitative polymerase chain reaction testing—changes during an epidemic. Thus, Ct values from even limited numbers of random samples can provide improved estimates of an epidemic’s trajectory. Combining data from multiple such samples improves the precision and robustness of this estimation. We apply our methods to Ct values from surveillance conducted during the severe acute respiratory syndrome coronavirus 2 (SARS-CoV-2) pandemic in a variety of settings and offer alternative approaches for real-time estimates of epidemic trajectories for outbreak management and response.

**Figure:**
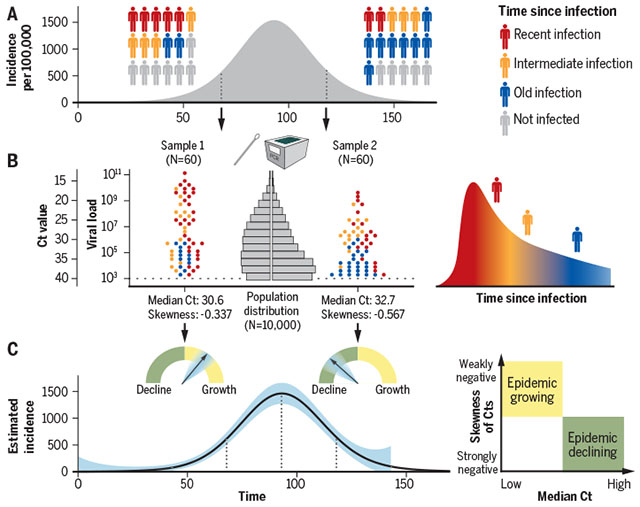
**Ct values reflect the epidemic trajectory and can be used to estimate incidence.** (**A** and **B**) Whether an epidemic has rising or falling incidence will be reflected in the distribution of times since infection (A), which in turn affects the distribution of Ct values in a surveillance sample (B). (**C**) These values can be used to assess whether the epidemic is rising or falling and estimate the incidence curve.

Real-time tracking of the epidemic trajectory and infection incidence is fundamental for public health planning and intervention during a pandemic (1, 2). In the severe acute respiratory syndrome coronavirus 2 (SARS-CoV-2) pandemic, key epidemiological parameters, such as the effective reproductive number *R_t_* have typically been estimated using the time series of observed case counts, hospitalizations, or deaths, usually on the basis of reverse transcription quantitative polymerase chain reaction (RT-qPCR) testing. However, limited testing capacities, changes in test availability over time, and reporting delays all influence the ability of routine testing to detect underlying changes in infection incidence (3–5). The question of whether changes in case counts at different times reflect epidemic dynamics or simply changes in testing have economic, health, and political ramifications.

RT-qPCR tests provide semiquantitative results in the form of cycle threshold (Ct) values, which are inversely correlated with log_10_ viral loads, but they are often reported only as binary “positives” or “negatives” (6, 7). It is common when testing for other infectious diseases to use this quantification of sample viral load, for example, to identify individuals with higher clinical severity or transmissibility (8–11). For SARS-CoV-2, Ct values may be useful in clinical determinations about the need for isolation and quarantine (7, 12), identification of the phase of an individual’s infection (13, 14), and predictions of disease severity (14, 15). However, individual-level decision-making on the basis of Ct values has not been widely implemented, owing to measurement variation across testing platforms and samples and a limited understanding of SARS-CoV-2 viral kinetics in asymptomatic and presymptomatic infections. Although a single high Ct value may not guarantee a low viral load in one specimen—for example, because of variable sample collection—measuring high Ct values in many samples will indicate a population with predominantly low viral loads. Cross-sectional distributions of Ct values should therefore represent viral loads in the underlying population over time, which may coincide with changes in the epidemic trajectory. For example, a systematic increase in the distribution of quantified Ct values has been noted alongside epidemic decline (12, 14, 16).

Here, we demonstrate that Ct values from single or successive cross-sectional samples of RT-qPCR data can be used to estimate the epidemic trajectory without requiring additional information from test positivity rates or serial case counts. We demonstrate that population-level changes in the distribution of observed Ct values can arise as an epidemiological phenomenon, with implications for interpreting RT-qPCR data over time in light of emerging SARS-CoV-2 variants. We also demonstrate how multiple cross-sectional samples can be combined to improve estimates of population incidence, a measure that is often elusive without serological surveillance studies. Collectively, we provide metrics for monitoring outbreaks in real time—using Ct data that are collected but currently usually discarded—and our methods motivate the development of testing programs intended for outbreak surveillance.

## Relationship between observed Ct values and epidemic dynamics

**Fig. 1.**
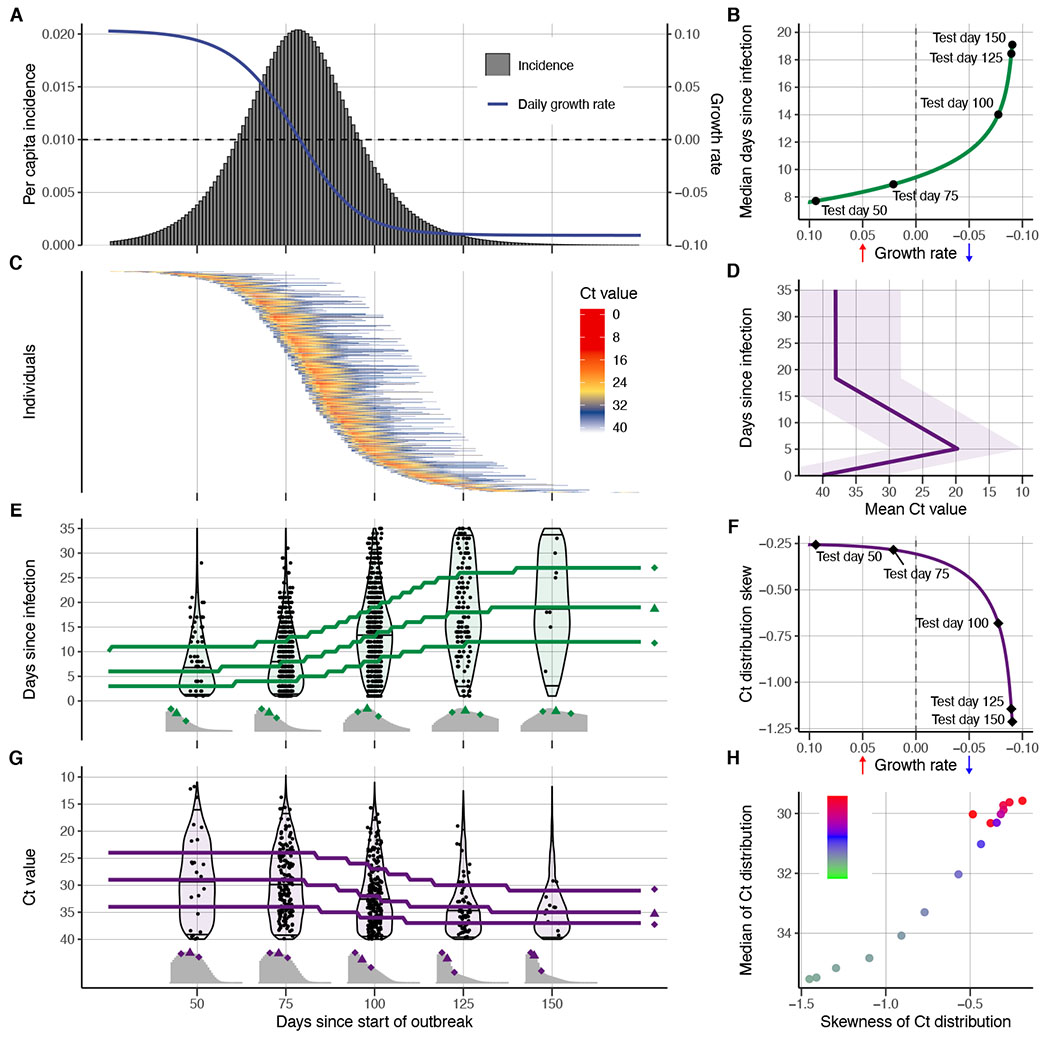
The Ct value distribution reflects epidemiological dynamics over the course of an outbreak. (**A**) Per capita daily incidence (histogram) and daily growth rate (blue line) of new infections in a simulated epidemic using an SEIR model. (**B**) Median days since infection versus daily growth rate of new infections by epidemic day. Labeled points here, and in (E) to (G), show five time points in the simulated epidemic. (**C**) Observed Ct value by day for 500 randomly sampled infected individuals. (**D**) Viral kinetics model (increasing Ct value after peak and subsequent plateau near the limit of detection), demonstrating the time course of Ct values (*x* axis; line shows mean, and ribbon shows 95% quantile range) against days since infection (*y* axis). Note that the *y* axis is arranged to align with (E). (**E**) Distribution of days since infection (violin plots and histograms) for randomly selected individuals over the course of the epidemic. Median and first and third quartiles are shown as green lines and points, respectively. (**F**) Skewness of observed Ct value distribution versus daily growth rate of new infections by epidemic day. (**G**) Distribution of observed Ct values (violin plots and histograms) among sampled infected individuals by epidemic day. Median and first and third quartile are shown as purple lines and points, respectively. (**H**) Time-varying effective reproductive number, *R*_*t*_, derived from the SEIR simulation, plotted against median and skewness of observed Ct value distribution.

First, we show that the interaction of within-host viral kinetics and epidemic dynamics can drive changes in the distribution of Ct values over time, without a change in the underlying pathogen kinetics. That is, population-level changes in Ct value distributions can occur without systematic changes in underlying postinfection viral load trajectories at the individual level. To demonstrate the epidemiological link between transmission rate and measured viral loads or Ct values, we first simulated infections arising under a deterministic susceptible-exposed-infectious-recovered (SEIR) model (Fig. 1A and Materials and methods, “Epidemic transmission models”). Parameters used are supplied in table S1. At selected testing days during the outbreak, simulated Ct values are observed from a random cross-sectional sample of the population using the Ct distribution model described in the “Ct value model” section of the Materials and methods and shown in figs. S1 and S2. By drawing simulated samples for testing from the population at specific time points, these simulations recreate realistic cross-sectional distributions of detectable viral loads across the course of an epidemic. Throughout, we assume everyone is infected at most once, ignoring reinfections because these appear to be a negligible portion of infections in the epidemic so far (17).

Early in the epidemic, infection incidence grows rapidly, and thus most infections arise from recent exposures. As the epidemic wanes, however, the average time elapsed since exposure among infected individuals increases as the rate of new infections decreases (Fig. 1, B and E) (18). This is analogous to the average age being lower in a growing versus declining population (19). Although infections are usually unobserved events, we can rely on an observable quantity, such as viral load, as a proxy for the time since infection. Because Ct values change asymmetrically over time within infected hosts (Fig. 1C), with peak viral load occurring early in the infection, a random sampling of individuals during epidemic growth is more likely to sample recently infected individuals in the early phase of their infection and therefore with higher quantities of viral RNA. Conversely, randomly sampled infected individuals during epidemic decline are more likely to be in the later phase of infection, typically sampling lower quantities of viral RNA, although there is substantial sampling and viral load variability at all time points (Fig. 1D). The overall distribution of observed Ct values under randomized surveillance testing therefore changes over time, as measured by the median, quartiles, and skewness (Fig. 1G). Although estimates for an individual’s time since infection based on a single Ct value will be highly uncertain, the population-level distribution of observed Ct values will vary with the growth rate—and therefore *R_t_*—of new infections (Fig. 1, F and H).

To summarize this key observation in the context of classic results, we find that fast-growing epidemiologic populations (*R_t_* > 1 and growth rate *r* > 0) will have a predominance of new infections and thus of high viral loads, and shrinking epidemics (*R_t_* < 1 and *r* < 0) will have more older infections and thus low viral loads at a given cross section, where the relationship between *R_t_* and *r* is modulated by the distribution of generation intervals (20). Similar principles have been applied to serologic data to infer unobserved individual-level infection events (16, 21–23) and population-level parameters of infectious disease spread (21, 24–28).

We find that this phenomenon might also be present, though less pronounced, among Ct values obtained under symptom-based surveillance, where individuals are identified and tested after symptom onset. Similar to the case of random surveillance testing, Ct values obtained through the testing of recently symptomatic individuals are predicted to be lower (i.e., viral loads are higher) during epidemic growth than those obtained during epidemic decline (figs. S3 and S4). However, defining the exact nature and strength of this relationship will depend on a number of conditions being met (fig. S4, caption).

By modeling the variation in observed Ct values arising from individual-level viral growth and clearance kinetics and sampling errors, the distribution of observed Ct values in a random sample becomes an estimable function of the times since infection, and the expected median and skewness of Ct values at a given point in time are then predictable from the epidemic growth rate. This function can then be used to estimate the epidemic growth rate from a set of observed Ct values. A relationship between Ct values and epidemic growth rate exists under most sampling strategies, as described above, but calibrating the precise mapping is necessary to enable inference (e.g., using a different RT-qPCR; fig. S5). This mapping can be confounded by testing biases arising, for example, from delays between infection and sample collection date when testing capacity is limited or through systematic bias toward samples with higher viral loads, such as those from severely ill individuals. Here, we focus on the case of random surveillance testing, where individuals are sampled at a random point in their infection course.

## Inferring the epidemic trajectory using a single cross section

From these relationships, we derive a method to infer the epidemic growth rate given a single cross section of randomly sampled RT-qPCR test results. The method combines two models: (i) the probability distribution of observed Ct values (and the probability of a negative result) conditional on the number of days between infection and sampling and (ii) the likelihood of being infected on a given day before the sample date. For the first, we use a Bayesian model and define priors for the mode and range of Ct values after infection on the basis of the existing literature (Materials and methods, “Ct value model” and “Single cross section model”). For the second, we initially develop two models to describe the probability of infection over time: (i) constant exponential growth of infection incidence and (ii) infections arising under an SEIR model. Both models provide estimates for the epidemic growth rate but make different assumptions regarding the possible shape of the outbreak trajectory: The exponential growth model assumes a constant growth rate over the preceding 5 weeks and requires few prior assumptions, whereas the SEIR model assumes that the growth rate changes daily depending on the remaining number of susceptible individuals but requires more prior information.

**Fig. 2.**
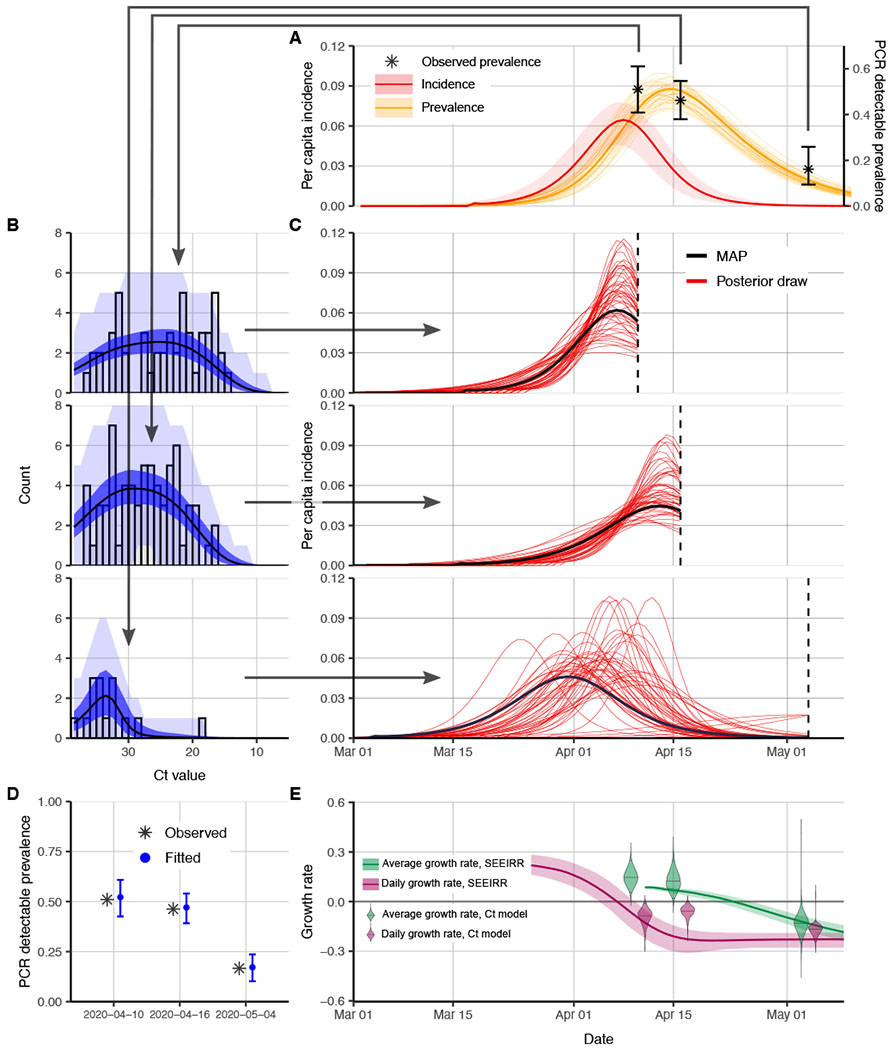
Single cross-sectional distributions of observed Ct values can be used to reconstruct epidemic trajectories in a Massachusetts long-term care facility. (**A**) Estimated prevalence [faint orange lines show posterior samples, solid orange line shows posterior median, and orange ribbon shows 95% credible intervals (CrIs)] and incidence (red line shows posterior median and red ribbon shows 95% CrI) from the standard compartmental (SEEIRR) model fit to point prevalence at three sampling times (error bars show 95% binomial confidence intervals). (**B**) Model-predicted Ct distributions (blue) fitted to the observed Ct values (gray bars) from each of three cross-sectional samples. Shown are the posterior median (black line) and 95% CrI for the expected Ct distribution (dark blue ribbon) and 95% prediction intervals based on simulated observations (light blue ribbon). Note that prediction intervals are much wider than CrIs because they result from simulating observations with a small sample size. (**C**) Each panel shows results from fitting the Ct-based SEIR model separately to three cross sections of virologic data. Shown are random posterior samples (red lines) and the maximum posterior probability (MAP) trajectory (black line) for the incidence curve. (**D**) Fitted median (blue point) and 95% CrI (blue error bars) for the proportion of samples testing positive based on the Ct model compared with the observed proportion tested positive (gray cross). (**E**) Thirty-five–day (green) and 1-day (pink) average growth rates from the Ct model estimates in (C) at three time points (violin plots) compared with growth-rate estimates from the SEEIRR model in (A) (lines and shaded ribbons).

To demonstrate the potential of this method with a single cross section from a closed population, we first investigate how the distribution of Ct values and prevalence of PCR positivity changed over time in four well-observed Massachusetts long-term care facilities that underwent SARS-CoV-2 outbreaks in March and April of 2020 (29). In each facility, we have the results of near-universal PCR testing of residents and staff from three time points after the outbreak began, including the number of positive samples, the Ct values of positive samples, and the number of negative samples (Materials and methods, “Long-term care facilities data”). To benchmark our Ct value-based estimates of the epidemic trajectory, we first estimated the trajectory using a standard compartmental modeling approach fit to the measured point prevalences over time in each facility (Fig. 2A). Specifically, we fit a simple extended SEIR (SEEIRR) model, with additional exposed and recovered compartments describing the duration of PCR positivity (Materials and methods, “Epidemic transmission models”), to the three observed point prevalence values from each facility. Because the testing was nearly universal, this approach provides a near ground truth of the epidemic trajectory, against which we can evaluate the accuracy of the Ct value–based approaches. We call this the baseline estimate. Figure 2 shows results and data for one of the long-term care facilities, and figs. S6 and S7 show results for the other three.

As time passes, the distribution of observed Ct values at each time point in the long-term care facilities (Fig. 2B) shifts higher (lower viral loads) and becomes more left skewed. We observed that these shifts tracked with the changing (i.e., declining) prevalence of infection in the facilities. To assess whether these changes in Ct value distributions reflected underlying changes in the epidemic growth rate, we fit the exponential growth and simple SEIR models using the Ct likelihood to each individual cross section of Ct values to get posterior distributions for the epidemic trajectory up to and at that point in time (Fig. 2C). The only facility-specific data for each of these fits were the Ct values and number of negative tests from each single cross-sectional sample. Additional ancillary information included prior distributions for the epidemic seed time (after 1 March) and the within-host virus kinetics. To assess the fit, we compare the predicted Ct distribution (Fig. 2B) and point prevalence (Fig. 2D) from each fit with the data and compare the growth rates from these fits with the baseline estimates. Posterior distributions of all Ct value model parameters are shown in fig. S8.

Although both sets of results are fitted models, and so neither can be considered the truth, we find that the Ct method fit to one cross section of data provides a similar posterior median trajectory to the baseline estimate, which required three separate point prevalences with near-universal testing at each time point. In particular, the Ct-based models appear to accurately discern whether the samples were taken soon or long after peak infection incidence. Both methods were in agreement over the direction of the past average and recent daily growth rates (i.e., whether the epidemic is currently growing or declining and whether the growth rate has dropped relative to the past average). The average growth-rate estimates were very similar between the prevalence-only and Ct value models at most time points, although the daily growth rate appeared to decline earlier in the prevalence-only compartmental model. These estimates have a great deal of variability, however, and should be interpreted in that context. This is especially clear in fig. S7, where the other facilities exhibit more variability between estimates from the two methods. Overall, these results show that a single cross section of Ct values can provide similar information to point-prevalence estimates from three distinct sampling rounds when the epidemic trajectory is constrained, as in a closed population.

To ensure that our method provides accurate estimates of the full epidemic curve, we performed extensive simulation-recovery experiments using a synthetic closed population undergoing a stochastic SEIR epidemic. Figure S9 shows the results of one such simulation, demonstrating the information gained from using a single cross section of virological test data when attempting to estimate the true infection incidence curve at different points during an outbreak. We assessed performance using simulated data from populations of different sizes and varied key assumptions of the inference method. Specifically, we implemented a version of the method that uses only positive Ct values without information on the fraction positive and tested the impact using prior distributions of decreasing strengths. Details are provided in the “simulated long-term care facility outbreaks” section of the supplementary materials, and results are in figs. S10 to S12.

Although no real long-term care facility data were available to assess the method’s accuracy during the early phase of the epidemic out-break, the simulation experiments reveal that the method can be used at all stages of an epidemic. Furthermore, although there is a substantial uncertainty in the growth-rate estimates, these analyses show that a single cross section of data can be used to determine whether the epidemic has been recently increasing or decreasing. The posterior probability of growth versus decline can be used for this assessment, acting like a hypothesis test when the credible interval excludes zero or in a broader inferential way if it does not. Although this is a trivial result for SARS-CoV-2 incidence in many settings, where cases, hospitalizations, or deaths already provide a clear picture of epidemic growth or decline, for locations and future outbreaks where testing capacity is restricted, our results show that a single cross-sectional random sample of a few hundred tested individuals combined with reasonable priors (for example, constraining the epidemic seed time to within a 1- to 2-month window) could be used to immediately estimate the stage of an outbreak. Moreover, this inferential method provides the basis for combining cross sections for multiple testing days.

## Inferring the epidemic trajectory using multiple cross sections

Although a single cross section of Ct values can reasonably estimate the trajectory of a simple outbreak represented by a compartmental model, more-complex epidemic trajectories will require more cross sections for proper estimation. Here, we extend our method to combine data from multiple cross sections, allowing us to estimate the full epidemic trajectory more reliably (Materials and methods, “Multiple cross sections model” and “MCMC framework”). In many settings, the epidemic trajectory is monitored using reported case counts. Limiting reported cases to those with positive test results, the daily number of new positives can be used to calculate *R_t_* (3). However, this approach can be obscured when the definition of a case changes during the course of an epidemic (30). Furthermore, such data often represent the growth rate of positive tests, which can change markedly on the basis of changing test capacity rather than the incidence of infection, requiring careful monitoring and adjustments to account for changes in testing capacity, the delay between infection and test report date, and the conversion from prevalence to incidence. Death counts are also used to estimate the epidemic trajectory, but these are substantially delayed, and the relationship between cases and deaths is not stable (31). When, instead, Ct values from surveillance sampling are available, our methods can overcome these limitations by providing a direct mapping between the distribution of Ct values and infection incidence. Although case-count methods exhibit bias as a result of changing test rates (5), our method provides a means to estimate *R_t_* using only one or a few surveillance samples, and this method can accommodate random sampling schemes that increase or decrease over time with test availability.

To demonstrate the performance of these Ct-based methods, we simulate outbreaks under a variety of testing schemes using SEIR-based simulations and sample Ct values from the outbreaks (Materials and methods, “Simulated testing schemes”). We compare the performance of *R_t_* estimation using reported case counts (based on the testing scheme) through the R package *EpiNow2* (32, 33)—where reporting depends on testing capacity and the symptom status of infected individuals—with the performance of our methods when one, two, or three surveillance samples are available with observed Ct values, with a total of ~0.3% of the population sampled (3000 tests spread among the samples).

**Fig. 3.**
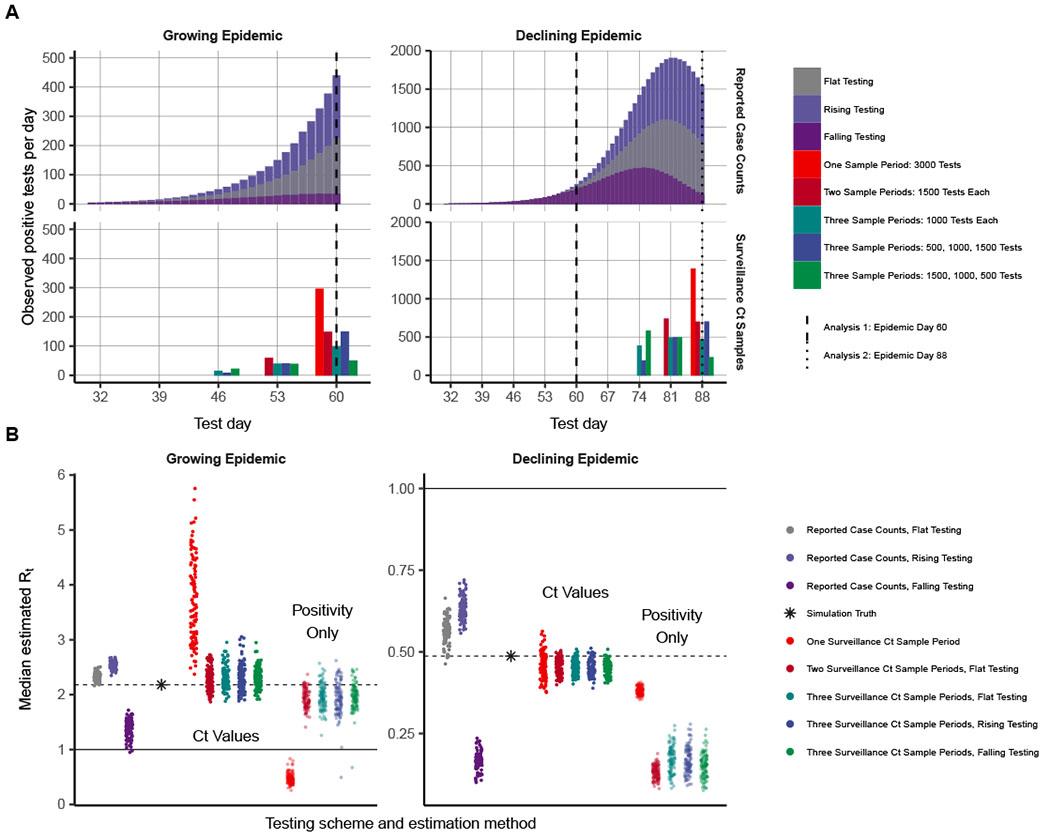
Inferring epidemic trajectory from cross-sectional surveillance samples with observed Ct values yields nearly unbiased estimates of the time-varying effective reproductive number, *R*_*t*_, whereas changing testing rates lead to biased estimation using reported case counts. (**A**) Number of positive tests per day by sampling time in epidemic and testing scheme for reported case counts (top row) and surveillance Ct sampling (bottom row), from a simulated SEIR epidemic. Analysis times corresponding to (**B**) are shown by the dashed vertical lines. (**B**) *R*_*t*_ estimates from 100 simulations for each epidemic sampling time, testing scheme, and estimation method. Each point is the posterior median from a single simulation. *R*_*t*_ estimates for reported case counts use *EpiNow2* estimation and for surveillance Ct samples use the Ct-based likelihood for one or multiple cross sections fitted to an SEIR model. The semitransparent points at the right of the plots are those surveillance samples fit to an SEIR model using only a binary result of testing, assuming PCR positivity reflects the infectious compartment. True model-based *R*_*t*_ on the sampling day is indicated by the black star and dashed horizontal line, whereas an *R*_*t*_ of 1, indicating a flat outbreak, is indicated by the solid horizontal line.

Figure 3 plots the posterior median *R_t_* from each of the 100 simulations of each method when the epidemic is growing (day 60) and declining (day 88). Except when only one sample is used, the Ct-based methods fitting to an SEIR model exhibit minimal bias, even when the number of tests substantially changes across sample days. For the single-sample estimates during the growth phase, the posterior median estimates are shifted above the true value because a range of *R*_0_ values are consistent with the data—the prior density for *R*_0_ is uniform between 1 and 10 with a median of 5.5, which weights the posterior median higher than the true value. Methods based on reported case counts, on the other hand, consistently exhibit noticeable upward bias when testing rates increase over the observed period and substantial downward bias when testing rates decrease. The Ct-based methods do exhibit higher variability, however. This is captured by the Bayesian inference model, as all of the Ct-based methods achieve at least nominal coverage of the 95% credible intervals among these 100 simulations (fig. S13).

An alternative approach to estimating *R_t_* using case counts is to fit a standard compartmental model to the observed proportion of positive tests from a random sample. To demonstrate the value of incorporating Ct values rather than simply using positivity rates from a surveillance sample, we also compare the results with an SEIR model fit to point prevalence observed at the same sample times, assuming PCR positivity represents the infectious stage of the disease. In this alternative method, this misspecification of the SEIR model results in inaccurate *R_t_* estimates during the decline phase of the simulation (Fig. 3B). Although a more accurate model might distinguish the infectious stage and duration of PCR positivity, as in the SEEIRR model, this simple model represents an approach that might be used to infer incidence changes from prevalence data in the absence of a quantified relationship between infection state and PCR positivity.

We also assessed the precision of our estimates using smaller sample sizes and different deployment of tests among testing days for a given sample size. These comparisons are shown in fig. S13, which also compares the Ct-based method with the positivity-based estimation. The Ct-based method performs well in many cases with sample sizes as low as 200 to 500 tests. When testing is stable, reported case counts provide a more precise estimate of the trajectory. However, a small number of tests (e.g., the same number of tests as used for 1 day of routine case detection) devoted to two or three surveillance samples can provide unbiased estimation when reported case counts may be biased.

## Reconstructing complex incidence curves using Ct values

Simple epidemic models are useful to understand recent incidence trends when data are sparse or in relatively closed populations where the epidemic start time is approximately known (supplementary materials, “epidemic seed time priors”). In reality, however, the epidemic usually follows a more complex trajectory that is difficult to model parametrically. For example, the SEIR model does not account for the implementation or relaxation of nonpharmaceutical interventions and behavior changes that affect pathogen transmission unless explicitly specified in the model. For a more flexible approach to estimating the epidemic trajectory from multiple cross sections, we developed a third model for infection incidence, using a Gaussian process (GP) prior for the underlying daily probabilities of infection (34). The GP method provides estimated daily infection probabilities without making strong assumptions about the epidemic trajectory—assuming only that infection probabilities on contemporaneous days are correlated, with decreasing correlation at increasing temporal distances (supplementary materials, “epidemic transmission models”). Movie S1 demonstrates how estimates of the full epidemic trajectory, representing a simulation for the implementation and subsequent relaxation of nonpharmaceutical interventions, can be sequentially updated using this model as new samples become available over time. Movie S2 shows how the precision of the estimated epidemic curve decreases at smaller sample sizes, where 200 samples per week were sufficient to reliably track the epidemic curve. Movie S3 shows how the estimation remains accurate if sampling is only initiated partway through the epidemic.

**Fig. 4.**
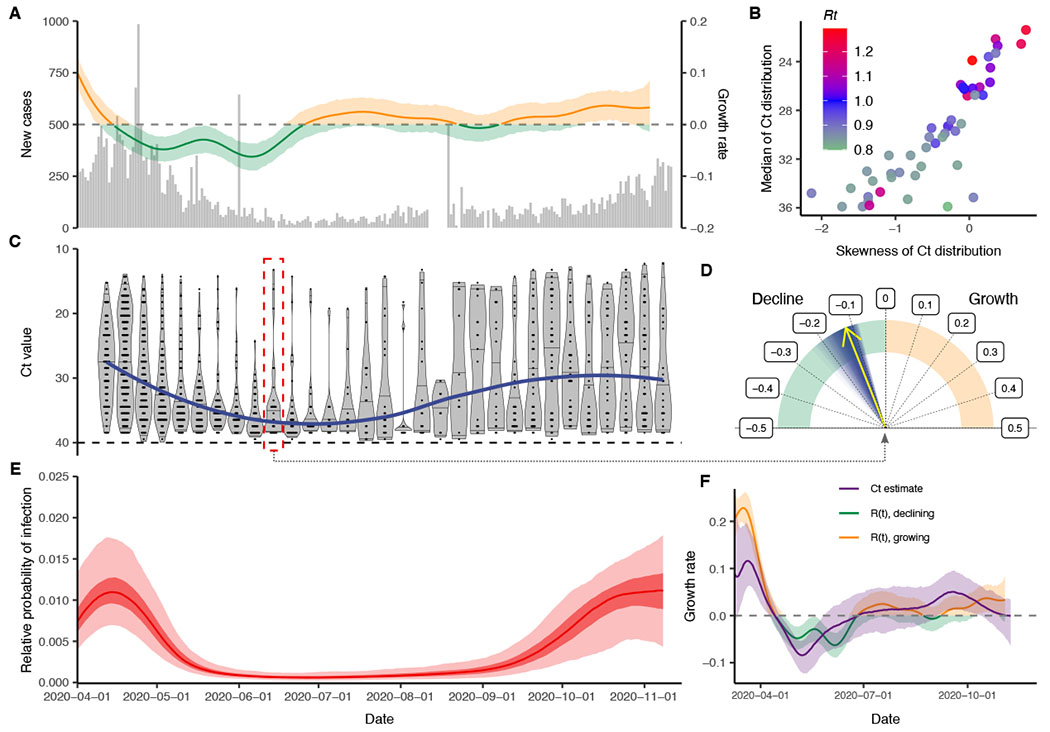
Cross-sectional distributions of observed Ct values can estimate the complex statewide epidemic trajectory from hospital-based surveillance at Brigham and Women’s Hospital in Massachusetts. (**A**) Daily confirmed new cases in Massachusetts (gray bars) and estimated time-varying effective reproductive number, *R*_*t*_. (B) Estimated *R*_*t*_ from the case counts versus median and skewness of observed Ct value distribution by weekly sampling times. (**C**) Distribution (violin plots and points) and smoothed median (blue line) of observed Ct values by sampling week. Red box highlights data used to inform estimates in (D). (**D**) Posterior median (yellow arrow) and distribution (blue shaded area) of estimated daily growth rate of incident infections from an SEIR model fit to a single cross section of observed Ct value data from the week commencing 14 June 2020. Shading density is proportional to posterior density. Fits to all single weekly cross sections are shown in fig. S14. (**E**) Posterior distribution of relative probability of infection by date from a GP model fit to all observed Ct values (ribbons show 95% and 50% CrIs; line shows posterior median). Note that the *y* axis shows relative rather than absolute probability of infection, as the underlying incidence curve must sum to one: Only positive samples were included in the estimation, and all samples were therefore assumed to have been from infections. (**F**) Comparison of estimated daily growth rate of incident infections from the GP model (blue line and shaded ribbons show posterior median and 95% CrI) to that from *R*_*t*_ estimation using observed case counts (red and green line and shaded ribbons show posterior median and 95% CrI) by date. Note that estimates of infection incidence are made for dates before the first observed sample date of 15 April 2020, as far back as 1 March 2020, but the *x* axis is truncated at 1 April 2020 (fig. S19).

With the objective of reconstructing the entire incidence curve using routinely collected RT-qPCR data, we used anonymized Ct values from positive samples measured from near-universal testing of all hospital admissions and nonadmitted emergency room (ER) patients in the Brigham and Women’s Hospital in Boston, Massachusetts, between 15 April and 10 November 2020 (Materials and methods, “Brigham and Women’s Hospital data”). We aligned these with estimates for *R_t_* based on case counts in Massachusetts (Fig. 4, A to C). The median and skewness of the detectable Ct distribution were correlated with *R_t_* (Fig. 4B), in line with our theoretical predictions (depicted in Fig. 1). The median Ct value rose (corresponding to a decline in median viral load) and skewness of the Ct distribution fell in the late spring and early summer, as shelter-in-place orders and other nonpharmaceutical interventions were rolled out (Fig. 4C), but the median declined and skewness rose in late summer and early fall as these measures were relaxed, coinciding with an increase in observed case counts for the state (Fig. 4A).

Using the observed Ct values, we estimated the daily growth rate of infections using the SEIR model on single cross sections (Fig. 4D and figs. S14 and S15) and the full epidemic trajectory using the GP model (Fig. 4E and fig. S16). Similar temporal trends were inferred under both models (fig. S17), and the GP model provided growth-rate estimates that followed those estimated using observed case counts (Fig. 4F). Although these data are not strictly a random sample of the community, and the observed case counts do not necessarily provide a ground truth for the *R_t_* value, these results demonstrate the ability of this method to recreate epidemic trajectories and estimate growth or decline of cases using only positive Ct values collected through routine testing. We assessed the robustness of the estimated GP trajectory to smaller sample sizes by refitting the model after subsampling different numbers of Ct values from the dataset (fig. S18). Notably, our estimated epidemic trajectory using only routinely generated Ct values from a single hospital was markedly correlated with changes in community-level viral loads obtained from wastewater data (fig. S19) (35).

## Discussion

The usefulness of Ct values for public health decision-making is currently the subject of much discussion and debate. One unexplained observation that has been consistently observed in many locations is that the distribution of observed Ct values has varied over the course of the current SARS-CoV-2 pandemic, which has led to questions over whether the fitness of the virus has changed (12, 14, 16). Our results demonstrate that this can be explained as an epidemiologic phenomenon, without invoking any change in individual-level viral kinetics or testing practices. This method alone, however, cannot prove that this is the case for any specific setting, as changing viral properties or changes in test availability may also lead to such shifts in Ct value distributions. We find that properties of the population-level Ct distribution strongly correlate with estimates for the effective reproductive number or growth rate in real-world settings, in line with our theoretical predictions.

Using quantitative diagnostic test results from multiple different tests conducted in a single cross-sectional survey, epidemic trends have previously been inferred from virological data (18). The methods we describe here use the phenomenon observed in the present pandemic and the relationship between incidence rate, time since infection, and virologic test results to estimate a community’s position in the epidemic curve, under various models of epidemic trajectories, based on data from one or more cross-sectional surveys using a single virologic test. Comparisons of simulated Ct values and observed Ct values with growth rates and *R_t_* estimates validate this general approach. Despite the challenges of sampling variability, individual-level differences in viral kinetics, and the limitations of comparing results from different laboratories or instruments, our results demonstrate that RT-qPCR Ct values, with all of their variability for an individual, can be highly informative of population-level dynamics. This information is lost when measurements are reduced to binary positive or negative classifications, as has been the case through most of the SARS-CoV-2 pandemic.

Here, we focused on the case of randomly sampling individuals from the population. This method will therefore be most useful in settings where representative surveillance samples can be obtained independently of COVID-19 symptoms, such as the REACT study in England (36). Even relatively small cross-sectional surveys, for example in a given city, may be very useful for understanding the direction that an out-break is heading. Standardized data collection and management across regions, along with wider use of random sampling, would further improve the usefulness of these methods, which demonstrate another use case for such surveillance (37, 38). These methods will allow municipalities to evaluate and monitor, in real time, the role of various epidemic mitigation interventions—for example, by conducting even a single or a small number of random virologic testing samples as part of surveillance rather than simply relying on routine testing results.

Extrapolation of these findings to Ct values obtained through strategies other than a population census or a mostly random sample requires additional considerations. When testing is based primarily on the presence of symptoms or contact-tracing efforts, infected individuals are more likely to be sampled at specific times since infection, which will affect the distribution of measured Ct values. Further complications arise when the delay between infection or symptom onset and sample collection changes over the course of the epidemic, for example because of a strain on testing capacity. Nonetheless, our simulation results suggest that the epidemic trajectory can still influence Ct values measured under symptom-based surveillance, although the strength of this association will depend on a number of additional considerations, as described in fig. S4. Additional work is needed to extend the inference methods presented here to use non-random surveillance samples.

The overall finding of a link between epidemic growth rates and measured Ct distributions is important for interpreting virologic data in light of emerging SARS-CoV-2 variants (39, 40). When samples are obtained through population-wide testing, an association between lower Ct values and emerging variants can be partially explained by those variants having a higher growth rate with a preponderance of recent infections compared with preexisting, declining variants. For example, a recent analysis of Ct values from P.1 and non-P.1 variant samples in Manaus, Brazil, initially found that P.1 samples had significantly lower Ct values (41). However, after accounting for the time between symptom onset and sample collection date (where shorter delays should lead to lower Ct values), the significance of this difference was lost. We caution that this finding does not exclude the possibility of newer variants causing infections with higher viral loads; rather, it highlights the need for lines of evidence other than surveillance testing data.

These results are sensitive to the true distribution of observed viral loads each day after infection. Different swab types, sample types, instruments, or Ct thresholds may alter the variability in the Ct distribution (15, 16, 42, 43), leading to different relationships between the specific Ct distribution and the epidemic trajectory. Where possible, setting-specific calibrations—for example, based on a reference range of Ct values—will help to generate precise estimates. This method will be most useful in cases where the population-level viral load kinetics can be estimated, either through direct validation or by comparison with a reference standard, for the instruments and samples used in testing. Here, we generated a viral kinetics model on the basis of observed properties of measured viral loads in the literature (proportion detectable over time after symptom onset, distribution of Ct values from positive specimens) and used these results to inform priors on key parameters when estimating growth rates. The growth-rate estimates can therefore be improved by choosing more precise, accurate priors relevant to the observations used during model fitting. In cases where results come from multiple testing platforms, the model should either be adjusted to account for this by specifying a different distribution for each platform on the basis of its properties or, if possible, the Ct values should be transformed to a common scale, such as log viral copies. If these features of the tests change substantially over time, results incorporating multiple cross sections might exhibit bias and will not be reliable.

Results could also be improved if individual-level features that may affect viral load, such as symptom status, age, and antiviral treatment, are available with the data and incorporated into the Ct value model (14–16, 44, 45). A similar approach may also be possible using serologic surveys, as an extension of work that relates time since infection to antibody titers for other infectious diseases (27, 28). If multiple types of tests (e.g., antigen and PCR) are conducted at the same time, combining information could substantially reduce uncertainty in these estimates (18). If variant strains are associated with different viral load kinetics and become common (40, 46), this should be incorporated into the model as well. Other features of the pathogen, such as the relationship between the viral loads of infector and infectee, might also affect population-level variability over time. Using virologic data as a source of surveillance information will require investment in better understanding Ct value distributions, as new instruments and techniques come online and as variants emerge, and in rapidly characterizing these distributions for future emerging infectious diseases. Remaining uncertainty can be incorporated into the Bayesian prior distribution.

This method has several limitations. Whereas the Bayesian framework incorporates the uncertainty in viral load distributions into inference on the growth rate, parametric assumptions and reasonably strong priors on these distributions aid in identifiability. If these parametric assumptions are violated—for example, when SEIR models are used across time periods when interventions likely affected transmission rates—inference may not be reliable. Additionally, the methods described here and the relationship between incidence and skewness of Ct distributions become less reliable when there are very few positive cases, so results should be interpreted with caution and sample sizes increased in periods with low incidence. In some cases, with one or a small number of cross sections, the observed Ct distribution could plausibly result from all individuals very early in their infection at the start of fast epidemic growth, all during the recovery phase of their infection during epidemic decline, or a mixture of both (Fig. 4E and fig. S15). We therefore used a parallel tempering Markov chain Monte Carlo (MCMC) algorithm for the single cross section estimates, which can accurately estimate these multimodal posterior distributions (47). Interpretation of the estimated median growth rate and credible intervals should be done with proper epidemiological context: Estimated growth rates that are grossly incompatible with other data can be safely excluded.

This method may also overstate uncertainty in the viral load distributions if results from different machines or protocols are used simultaneously to inform the prior. A more precise understanding of the viral load kinetics—in particular, modeling these kinetics in a way that accounts for the epidemiologic and technical setting of the measurements—will help improve this approach and determine whether Ct distribution parameters from different settings are comparable. Because of this, semi-quantitative measures from RT-qPCR should be reported regularly for SARS-CoV-2 cases, and early assessment of pathogen load kinetics should be a priority for future emerging pathogens. The use of control measurements, like using the ratio of detected viral RNA to detected human RNA, could also improve the reliability and comparability of Ct measures.

The Ct value is a measurement with magnitude, which provides information on underlying viral dynamics. Although there are challenges to relying on single Ct values for individual-level decision-making, the aggregation of many such measurements from a population contains substantial information. These results demonstrate how one or a small number of random virologic surveys can be best used for epidemic monitoring. Overall, population-level distributions of Ct values, and quantitative virologic data in general, can provide information on important epidemiologic questions of interest, even from a single cross-sectional survey. Better epidemic planning and more-targeted epidemiological measures can then be implemented on the basis of such a survey, or Ct values can be combined across repeated samples to maximize the use of available evidence.

## Materials and methods summary

### Long-term care facilities data

Data from Massachusetts long-term care facilities were nasopharyngeal specimens collected from staff and residents processed at the Broad Institute of MIT and Harvard CRSP CLIA laboratory, with an FDA (Food and Drug Administration) Emergency Use Authorized laboratory-developed assay. Ct values for N1 and N2 gene targets were provided along with sample collection date, a random tube ID, and a unique anonymized institute ID to reflect that specimens came from distinct institutions. The specimens used here originated in early 2020 when public health efforts in Massachusetts led to comprehensively serial testing senior nursing facilities as described previously (29). Swabs from those public health efforts were processed for clinical diagnostics. Sample collection dates ranged from 6 April 2020 to 5 May 2020, with each facility undergoing three sampling rounds. Each round took a median of 2 days (range, 1 to 6 days) to complete. The anonymized Ct data were made available, and the N2 Ct values were used for these analyses. For all analyses presented here, sample collection dates were grouped into sampling rounds and analyzed based on the mean collection date for that round (i.e., the dates shown in Fig. 2 and figs. S6 and S7).

### Brigham and Women’s Hospital data

Data from the Brigham and Women’s Hospital in Boston, Massachusetts, were nasopharyngeal specimens from patients processed on a Hologic Panther Fusion SARS-CoV-2 assay. Ct values for the ORF1ab gene were provided alongside sample collection date, with collection dates ranging from 3 April 2020 to 10 November 2020. For these analyses, we grouped samples by week of collection on the epidemiological calendar and used the midpoint of each week for the analyses shown in Fig. 4. Testing during the first 2 weeks in April 2020 was restricted to patients with symptoms consistent with COVID-19 and who needed hospital admission. After 15 April, testing criteria for this platform were expanded to include all asymptomatic hospital admissions, symptomatic patients in the emergency room who were not admitted to the hospital, and inpatients requiring testing who were not in labor. Symptomatic ER patients who were admitted to the hospital were tested on a different PCR platform and are not considered here. In the analyses presented here, we use only samples taken after 15 April. Although this is not a perfectly representative surveillance sample, the routine testing of hospital admissions who were not seeking COVID-19 treatment creates a cohort that is less biased than symptom-based testing and represents the overall rise and fall of cases in the hospital’s catchment area. Daily data are aggregated by week. Daily confirmed case counts for Massachusetts were obtained from *The New York Times,* based on information from state and local health agencies (48).

### Epidemic transmission models

Throughout these analyses, we used four mathematical models to describe daily SARS-CoV-2 transmission over the course of an epidemic. Full model descriptions are given in the “epidemic transmission models” section of the supplementary materials, and a brief overview is provided here in order of introduction in the main text. First, the *SEIR Model* is a compartmental model which assumes that the growth rate of new infections depends on the current prevalence of infectious and susceptible individuals by modeling the proportion of the population who are susceptible, exposed, infected, or recovered with respect to disease over time. Second, the *Exponential Growth Model* assumes that new infections arise under a constant exponential growth rate. Third, the *SEEIRR Model* is a modification of the SEIR model with additional compartments for individuals who are exposed but not yet detectable by PCR and individuals who are recovered but still detectable by PCR. Finally, the *Gaussian Process Model* describes the epidemic trajectory as a vector of daily infection probabilities, where a GP prior is used to ensure that daily infection probabilities are correlated in time; days that are chronologically close in time are more correlated than those that are chronologically distant.

### Ct value model

We developed a mathematical model describing the distribution of observed SARS-CoV-2 viral loads over time after infection. The model is described in full in the “Ct value model” section of the supplementary materials. This model is similar to that used by Larremore *et al.* (49), but allows for more flexibility in the decline of viral load during recovery. We used a parametric model describing the modal Ct value, *C*_mode_(*a*), for an individual *a* days after infection, represented by the solid black line in fig. S1B. The measured Ct value is a linear function of the log of the viral load in the sample, but we describe the model on the Ct scale to match the data. Because we are interested in the population-level distribution and not individual trajectories, we assumed that observed Ct values *a* days after infection, *C*(*a*), followed a Gumbel distribution with location (mode) parameter *C*_mode_(*a*) and scale parameter σ(*a*) that also may depend on the number of days *a* after infection. We chose a Gumbel distribution to capture overdispersion of high measured Ct values. This distribution captures the variation resulting from both swabbing variability and individual-level differences in viral kinetics. We note that at any point in the infection, there is a considerable amount of person-to-person and swab-to-swab variation in viral loads (50–52), including a possible difference by symptom status (15, 53, 54). Tracking individual-level viral kinetics would require a hierarchical model capturing individual-level parameters, but is not necessary for this analysis.

The rationale behind this parameterization and the chosen parameter values is discussed in the “selecting viral kinetics and compartmental model parameters” section of the supplementary materials. We note that in all analyses, we used informative priors for key features of viral load kinetics rather than fixing point estimates, incorporating uncertainty into our inference. The process for generating these priors is described in the “informing the viral kinetics model” section of the supplementary materials. We performed this calibration step separately for the long-term care facility and Brigham and Women’s Hospital datasets, as the gene targets and testing platform were different, and thus Ct values are not directly comparable.

### Relationship between observed Ct values and daily probability of infection

#### Single cross section model

For a single testing day *t*, let π_*t*−*A*_max__, …, π_*t*−1_ be the marginal daily probabilities of infection for the whole population for *A*_max_ days to 1 day before *t*, respectively, where *t* − *A*_max_ is the earliest day of infection that would result in detectable PCR values on the testing day. That is, π_*t*−*a*_ is the probability that a randomly selected individual in the population was infected on day *t* − *a*. Let *p_a_*(*x*) be the probability that the Ct value is *x* for a test conducted *a* days after infection given that the value is detectable (i.e., the Gumbel probability density function normalized to the observable values). Then *p_a_*(*x*) = *P*[*C*(*a*) = *x*]/*P*[0 ≤ *C*(*a*) < *C_LOD_*], where *P*[*C*(*a*) = *x*] is the Gumbel probability density function with location parameter *C*_mode_(*a*) and scale parameter σ(*a*). Let ϕ_*a*_ be the probability of a Ct value being detectable *a* days after infection, which depends on *C*(*a*) and any additional decline in detectability. Let the PCR test results from a sample of *n* individuals be recorded as *X*_1_, …, *X_n_*. Then, for *x_i_* < *C_LOD_* (i.e., a detectable Ct value), the probability of individual *i* having Ct value *x_i_* is given by:

$$P(X_{i}=x_{i}|\pi_{t-A_{\max}},\ldots ,\pi_{t-1})\;=\sum_{a=1}^{A_{\max}}p_{a}\left(x_{i}\right)\phi_{a}\pi_{t-a}$$

The probability of a randomly chosen individual being detectable to PCR on testing day *t* is:

$$P\left(X_{i}<C_{LOD}|\pi_{t-A_{\max}},\ldots ,\pi_{t-1}\right)=\sum_{a=1}^{A_{\max}}\phi_{a}\pi_{t-a}$$

So the likelihood for the *n* PCR values is given by:

$$L\left(X_{1},\ldots ,X_{n}|\pi_{t-A_{\max}},\ldots ,\pi_{t-a}\right)\;=\prod_{i=1}^{n}[{\left(\sum_{a=1}^{A_{\max}}p_{a}\left(X_{i}\right)\phi_{a}\pi_{t-a}\right)}^{I\left(X_{i}<C_{LOD}\right)}\;\times {\left(1-\sum_{a=1}^{A_{\max}}\phi_{a}\pi_{t-a}\right)}^{I\left(X_{i}<C_{LOD}\right)}]$$

where *I*(·) equals 1 if the interior statement is true and 0 if it is false.

If only detectable Ct values are recorded as *X*_1_,…,*X_n_*, then the likelihood function is given by:

$$L\left(X_{1},\ldots ,X_{n}|\pi_{t-A_{\max}},\ldots ,\pi_{t-1}\right)\;=\prod_{i=1}^{n}\left[\frac{\sum_{a=1}^{A_{\max}}p_{a}\left(X_{i}\right)\phi_{a}\pi_{t-a}}{\sum_{a=1}^{A_{\max}}\phi_{a}\pi_{t-a}}\right]\;=\frac{\prod_{i=1}^{n}\left[\sum_{a=1}^{A_{\max}}p_{a}\left(X_{i}\right)\phi_{a}\pi_{t-a}\right]}{{\left(\sum_{a=1}^{A_{\max}}\phi_{a}\pi_{t-a}\right)}^{n}}$$

Either of these likelihoods can be maximized to get nonparametric estimates of the daily probability of infection, with the constraint that $\sum_{a=1}^{A_{\max}}\pi_{t-a}\leq 1$. To improve power and interpretability of the estimates, however, we consider two parametric models based on the epidemic transmission models described above: (i) a model assuming exponential growth of infection incidence over a defined period before the sampling day and (ii) an SEIR compartmental model in a closed finite population, where the basic reproduction number *R*_0_ is a parameter estimated by the model but does not vary over time (i.e., there are no interventions that reduce transmissibility). See the “parametric models for fitting cross-sectional viral load data” section of the supplementary materials for details of the likelihoods used in these methods.

#### Multiple cross sections model

Now we consider settings where there are multiple days of testing, *t*_1_,…,*t_T_*. We again denote by π_*t*_ the probability of infection on day *t* and now denote the sampled Ct value for the *i*th individual sampled on test day *t_j_* by $X_{i}^{t_{j}}$, where *i* ∈ 1,…,*n_j_* for test day *j* and *j* ∈ 1,…,*T*. Note that individual *i* may refer to different individuals on different testing days. Let {π_*t*_} be the daily probabilities of infection for any day *t* where an infection on day t could be detectable using a PCR test on one of the testing days. By a straightforward extension of the likelihood for the single cross section model, the non-parametric likelihood for the set of infection probabilities {π_*t*_}, when samples with and without a detectable Ct value are included, is given by:

$$L\left(X_{1}^{t_{1}},\ldots X_{n_{1}}^{t_{1}},\ldots ,X_{n_{T}}^{t_{T}}|\left\{\pi_{t}\right\}\right)\;=\prod_{j=1}^{T}\{\prod_{i=1}^{n_{j}}[{\left(\sum_{a=1}^{A_{\max}}p_{a}\left(X_{i}^{t_{j}}\right)\phi_{a}\pi_{t_{j}-a}\right)}^{I\left(X_{i}^{t_{j}}<C_{LOD}\right)}\times {\left(1-\sum_{a=1}^{A_{\max}}\phi_{a}\pi_{t_{j}-a}\right)}^{I\left(X_{i}^{t_{j}}\geq C_{LOD}\right)}]\}\;=\prod_{j=1}^{T}\{[\prod_{i=1}^{n_{j}}{\left(\sum_{a=1}^{A_{\max}}p_{a}\left(X_{i}^{t_{j}}\right)\phi_{a}\pi_{t_{j}-a}\right)}^{I\left(X_{i}^{t_{j}}<C_{LOD}\right)}]\times {\left[1-\sum_{a=1}^{A_{\max}}\phi_{a}\pi_{t_{j}-a}\right]}^{n_{j}^{-}}\}$$

where $n_{j}^{-}$ is the number of undetectable samples on testing day *t_j_*.

Only considering samples with a detectable Ct value gives the likelihood:

$$L\left(X_{1}^{t_{1}},\ldots X_{n_{1}}^{t_{1}},\ldots ,X_{n_{T}}^{t_{T}}|\left\{\pi_{t}\right\}\right)\;=\prod_{j=1}^{T}\left\{\frac{\prod_{i=1}^{n_{j}}\left[\sum_{a=1}^{A_{\max}}p_{a}\left(X_{i}^{t_{j}}\right)\phi_{a}\pi_{t_{j}-a}\right]}{{\left[\sum_{a=1}^{A_{\max}}\phi_{a}\pi_{t_{j}-a}\right]}^{n_{j}}}\right\}$$

Either of these likelihoods can be parameterized using the exponential growth rate model described above. However, the exponential growth rate model is less likely to be a good approximation of the true incidence probabilities over a longer period of time, so it may not be a good model for multiple test days that cover a long stretch of time.

The multiple cross section likelihood is primarily used to fit the GP model, estimating the daily probability of infection, {π_*t*_}, conditional on the set of observed Ct values. (supplementary materials, “parametric models for fitting cross-sectional viral load data”). The SEIR model can be used with multiple testing days as well. It is fit as described for the single cross section model, but with one of these likelihoods in place of the single cross section model likelihood, with posterior distribution estimates obtained through MCMC fitting.

### MCMC framework

All models, including those using Ct values (*SEIR Model*, *Exponential Growth Model*, and the *Gaussian Process Model*) and those using only prevalence (*SEIR Model* and *SEEIRR Model*) were fitted using a MCMC framework. We used a Metropolis-Hastings algorithm to generate either multivariate Gaussian or univariate uniform proposals. For all single cross section analyses (Figs. 2 and 3), we used a modified version of this framework with parallel tempering: an extension of the algorithm that uses multiple parallel chains to improve sampling of multimodal posterior distributions (47). For the multiple cross section analyses including those in Fig. 4, we used the unmodified Metropolis-Hastings algorithm because the computational time of the parallel tempering algorithm is far longer, and these analyses were underpinned by more data and less affected by multimodality. In all analyses, three chains were run upward of 80,000 iterations (500,000 iterations for the GP models). Convergence was assessed based on all estimated parameters having an effective sample size greater than 200 and a potential scale reduction factor ($\hat{R}$) of <1.1, evaluated using the *coda* R package (55). All assumed prior distributions are described in table S1.

### Simulated data

All simulated data were generated under the same framework but with different models and assumptions for the underlying epidemic trajectory. For each simulation, data were generated in four steps: (i) the daily probability of transmission, {π_*t*_}, is calculated using either a deterministic SEIR model, a stochastic SEIR model, or a GP model; (ii) on each day of the simulation, new infections are simulated under the model *I_t_* ~ *Binomial*(*N*, π_*t*_), where *N* is the population size of the simulation and *I_t_* is the number of new infections on day *t* (all other individuals are assumed to have escaped infection); (iii) a subset of individuals are sampled on particular days of the simulation determined by the testing schemes described below and in the “comparison of analysis methods” section of the supplementary materials; and (iv) for each individual sampled on day *u*, a Ct value was simulated under the model *X_i_* ~ *Gumbel*[*C*_mode_(*u* − *t_inf_*), σ(*u* − *t_inf_*)], where *t_inf_* is the time of infection for individual *i*. *C*_mode_(*u* − *t*) and σ(*u* − *t*) are described in the “Ct value model” section of the supplementary materials.

### Simulated testing schemes

Standard approaches to estimating doubling time, growth rate, or *R_t_* are subject to misestimation as a result of changes in testing policies (5). To assess the effect of such changes on our methods, we simulate changes in testing rates and assess the effect on several methods for *R_t_* estimation: using *EpiNow2* with reported case counts (33), using Ct-based methods with random surveillance samples, and using PCR test positivity alone with surveillance samples. We test these methods at two periods of an outbreak—once when the epidemic is rising and once when it is falling. For the random samples for each of these analysis time points, we test from 1 to 3 days of sampling for virologic testing with varying sample sizes across the test days. Results are shown in Fig. 3 and fig. S13; more details are in the “comparison of analysis methods” section of the supplementary materials.

## Supplementary Material

movieS1

movieS2

movieS3

S1data

S2data

supplementary_material_1
