# Supplementary material for: Estimating epidemiologic dynamics from cross-sectional viral load distributions
Source: Science. Author manuscript; Available in PMC 2021 Oct 20. (PMC8527857; doi:10.1126/science.abh0635)
Supplement: supplementary_material_1 [file NIHMS1741625-supplement-supplementary_material_1.pdf]

science.sciencemag.org/cgi/content/full/science.abh0635/DC1

Supplementary Materials for

Estimating epidemiologic dynamics from cross-sectional viral load distributions

James A. Hay*†, Lee Kennedy-Shaffer*†, Sanjat Kanjilal, Niall J. Lennon, Stacey B. Gabriel, Marc Lipsitch, Michael J. Mina*

*Corresponding author. Email: jhay@hsph.harvard.edu (J.A.H.); lkennedyshaffer@vassar.edu (L.K.-S.); mmina@hsph.harvard.edu (M.J.M.)
†These authors contributed equally to this work.

Published 3 June 2021 on *Science* First Release
DOI: 10.1126/science.abh0635

This PDF file includes:

Materials and Methods
Figs. S1 to S19
Table S1
Captions for Movies S1 to S3
Captions for Data S1 and S2
References

Other Supplementary Material for this manuscript includes the following:
(available at science.sciencemag.org/cgi/content/full/science.abh0635/DC1)

MDAR Reproducibility Checklist (.pdf)
Movies S1 to S3 (.mov)
Data S1 and S2 (.csv)

Materials and Methods

Epidemic Transmission Models

SEIR Model

We implemented a standard deterministic SEIR model with compartments for susceptible (S), exposed-not-infectious (E), infectious (I) and recovered (R). This is the model we use to generate Fig. 1, to estimate incidence based on Ct distributions as shown in Fig. 2C, for the model fits shown in Fig. 3 and the single-cross section results in Fig 4. Note that this model does not account for PCR detectability, as the time course of viral loads is modelled separately. The compartmental transition equations are given by:

$$\frac{dS}{dt} = \frac{-\beta SI}{N},$$
$$\frac{dE}{dt} = \frac{\beta SI}{N} - \sigma E,$$
$$\frac{dI}{dt} = \sigma E - \gamma I, \text{ and}$$
$$\frac{dR}{dt} = \gamma I,$$

where $\beta = \frac{R_0}{\gamma}$, and $\beta = 0$ for $t < t_0$. For the Ct model fitting, the daily probability of infection, π_t, is given by the daily per-capita increase in the cumulative exposed compartment, $\frac{dE'}{dt} = \frac{\beta SI}{N}$. For the long-term care facility estimates and corresponding simulations, we assumed that the initial prevalence of infected individuals at the time of seeding was 0.2%. For the Brigham & Women's hospital estimates and corresponding simulations, we assumed an initial seed prevalence of 0.001%.

To represent the implementation and relaxation of non-pharmaceutical interventions which decrease transmission rates, we modified the model to assume that the underlying transmission probability parameter, β, was a function of time, t. This was the model used for the simulations shown in Fig. S4, Fig. S18, and Movies S1 to S3. For the purposes of simulation, we solved this modified model in a stochastic framework (rather than deterministic as above) using the *odin* R package (*60*). Here, we assumed that the transmission rate changed over time as follows:

$$\beta_t = \begin{cases} R_0\gamma & t < 80 \\ R_0^2\gamma & 80 \le t < 150 \\ R_0^3\gamma & t > 150 \end{cases}$$

where $R_0 = 2.5$, $R_0^2 = 0.7$ and $R_0^3 = 1.5$. For this simulation, we assumed that the initial prevalence of infected individuals at time of seeding was 0.001%. To smooth the transitions between these different transmission rates, we fitted a cubic smoothing spline to β_t using the

‘smooth.spline’ function in R version 4.0.2 with a smoothing parameter of 0.5 before solving the ODEs.

Exponential Growth Model

Under the exponential growth model, new infections were assumed to arise with daily probability given by $\pi_t = \pi_0 e^{rt}$, where t is in unit days. When fitting the model, we parameterized π_0 to find the overall probability of infection with the focal period as $\pi_0 = \frac{c}{\sum_0^{t_{max}} e^{rt}}$ where c is the overall probability of infection between $t = 0$ and $t = t_{max}$.

SEEIRR Model

To describe the prevalence of individuals who are PCR positive for SARS-CoV-2, we developed a deterministic SEEIRR transmission model with states for susceptible (*S*), exposed-not-infectious-undetectable (E_1), exposed-not-infectious-detectable (E_2), infected-infectious-detectable (*I*), recovered-not-infectious-detectable (R_1) and recovered-not-infectious-undetectable (R_2), as depicted in Fig. S1A. These additional exposed and recovered compartments account for the periods when an individual will test PCR positive, but is not yet or is no longer infectious. We fit this model to data from the four Massachusetts long-term care facilities using Markov chain Monte Carlo (MCMC) to generate the trajectories in Fig. 2A, placing informative priors on the shared transition rate parameters (Table S1) and uniform priors on the facility-specific basic reproductive numbers, R_0, and the effective seed times, t_0. The compartmental transition equations are given by:

$$\frac{dS}{dt} = \frac{-\beta_l SI}{N},$$
$$\frac{dE_1}{dt} = \frac{\beta_l SI}{N} - \sigma' E,$$
$$\frac{dE_2}{dt} = \sigma' E_1 + \alpha E_2,$$
$$\frac{dI}{dt} = \alpha E_2 - \gamma' I,$$
$$\frac{dR_1}{dt} = \gamma' I - \omega R_1, \text{ and}$$
$$\frac{dR_2}{dt} = \omega R_1,$$

where β_l is the location specific transmission rate and $\beta_l = 0$ for $t < t_{0,l}$, the location specific seed time. The initial prevalence of infected individuals at the time of seeding was assumed to be 0.2%.

Gaussian Process Model

For a highly general version of our method, we developed a flexible model for the daily probability of infection, denoted π_t, for any day t where infection on that day could result in a positive PCR test on the (or one of the) testing day(s). Specifically, we use a Gaussian process prior for a vector of values k_t for each day t; the incidence rate for day t is then given by $\pi_t = (1 + e^{-k_t})^{-1}$. The covariance matrix for the GP prior $k \sim MVN\big((0, \dots, 0), \boldsymbol{K}\big)$ is given by $\boldsymbol{K}_{ij} = \eta^2 \exp\big(-\rho^2 D_{ij}^2\big)$, where D_{ij} is the difference between i and j and ν and ρ are hyperparameters fixed to values of 1.5 and 0.03, respectively (see Table S1). The parameter ρ determines the rate of decline of the covariance as the time between days increases, so a higher value of ρ indicates less correlation. See McElreath for more details (*61*).

This method is more flexible than one based on the SEIR model, allowing the daily incidence rate to reflect changes in transmission rates and contact patterns (although these are not separately identified by the model), as well as the depletion of the pool of susceptible individuals. Thus, it does not require the strict parametric assumptions of the SEIR model. We use it to model the course of the outbreak in Massachusetts, as various policies and behavior changes affect the trajectory over time.

Ct Value Model

The modal Ct value at day a follows a two-hinge function that is at the true undetectable value of C_{zero} for $a \le t_{eclipse}$, decreases linearly (log viral load increases) to a minimum Ct of C_{peak} at $a = t_{eclipse} + t_{peak}$, increases linearly (log viral load wanes) to a Ct value of C_{switch} at $a = t_{eclipse} + t_{peak} + t_{switch}$, and then increases (log viral load wanes) at a slower linear rate until it reaches the limit of detection (LOD), C_{LOD}, at $a = t_{LOD}$. That is, the modal Ct value is given by:

$$C_{mode}(a) = \begin{cases} C_{zero}, & a \le t_{eclipse} \\ C_{zero} + \dfrac{C_{peak} - C_{zero}}{t_{peak}}\left(a - t_{eclipse}\right), & t_{eclipse} < a \le t_{eclipse} + t_{peak} \\ C_{peak} + \dfrac{C_{switch} - C_{peak}}{t_{switch}}\left(a - t_{eclipse} - t_{peak}\right), & t_{eclipse} + t_{peak} < a \le t_{eclipse} + t_{peak} + t_{switch} \\ C_{switch} + \dfrac{C_{LOD} - C_{switch}}{t_{LOD} - t_{switch} - t_{peak} - t_{eclipse}}\left(a - t_{eclipse} - t_{peak} - t_{switch}\right), & t_{eclipse} + t_{peak} + t_{switch} < a \end{cases}$$

The Ct value of a randomly-chosen individual a days after infection, $C(a)$, is then distributed according to: $C(a) \sim Gumbel\big(C_{mode}(a), \sigma(a)\big)$, where $\sigma(a)$ is given by:

$$\sigma(a) = \begin{cases} \sigma_{obs}, & a < t_{eclipse} + t_{peak} + t_{switch} \\ \sigma_{obs}\left[1 - \dfrac{1 - s_{mod}}{t_{mod}}\left(a - t_{eclipse} - t_{peak} - t_{switch}\right)\right], & t_{eclipse} + t_{peak} + t_{switch} \le a < t_{eclipse} + t_{peak} + t_{switch} + t_{mod} \\ \sigma_{obs} s_{mod}, & t_{eclipse} + t_{peak} + t_{switch} + t_{mod} \le a \end{cases}$$

Note that for the Gumbel distribution, the mean and variance are given by $C_{mode}(a) - \sigma(a)\gamma$ and $\frac{\sigma(a)^2\pi^2}{6}$, respectively, where γ is Euler's constant.

This observation model allows for Ct values to gradually shrink towards the mode as the infection is cleared and most individuals become undetectable again. The log-viral load (in $\log_{10}$ RNA copies per mL) a days after infection, $V(a)$, can then be calculated by: $V(a) = V_{LOD} + \frac{C_{LOD} - C(a)}{\log_2(10)}$, where the limit of detection on the Ct scale is $C_{LOD} = 40$ and on the viral load scale depends on test characteristics (we assume $V_{LOD} = 3$ $\log_{10}$ RNA copies per mL). This model captures the shape of the observed modal viral load over time and the features described above.

A feature of viral loads not captured by the above model is that a small fraction of individuals remain PCR positive at very high Ct values for many weeks after recovery, whereas most drop to undetectable levels within a couple of weeks. To account for this, each day after $t_{eclipse} + t_{peak} + t_{switch}$ there is a daily probability, p_{addl}, of an individual fully clearing the virus and becoming undetectable, in addition to the viral load trajectory where the modal Ct value rises above the limit of detection. The probability of being detectable on day $a \leq t_{eclipse} + t_{peak} + t_{switch}$ is $\phi_a = P[C(a) < C_{LOD}]$ and the probability of being detectable on day $a > t_{eclipse} + t_{peak} + t_{switch}$ is $\phi_a = P[C(a) < C_{LOD}](1 - p_{addl})^{a - t_{eclipse} - t_{peak} - t_{switch}}$.

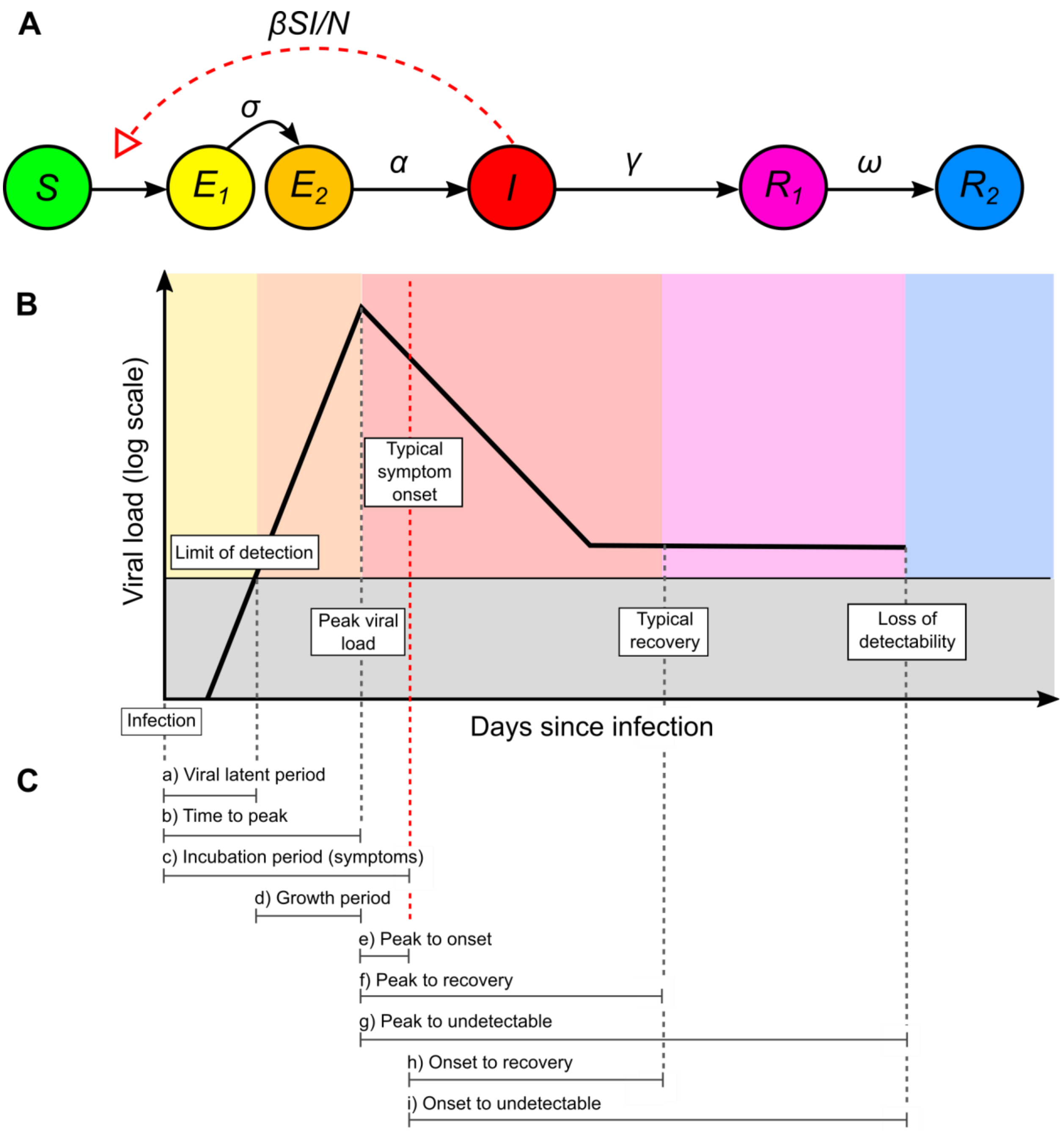

Fig. S1. Schematic of the SEEIRR transmission model, viral kinetics model and periods of viral kinetics used to parameterize the model. (**A**) Transmission model with the following compartments: susceptible (S), exposed, not infectious, undetectable (E_1), exposed, not infectious, detectable (E_2), infectious and detectable (I), recovered still detectable (R_1), and recovered undetectable (R_2). Transition rates were as follows: β, the transmission rate, σ, onset of detectability rate, α, the onset of infectiousness rate, γ, the rate of infectiousness loss, and ω, the rate of loss of detectability after recovery. The red dashed arrow denotes the force of infection exerted on susceptible individuals. Note that the simpler SEIR model effectively combined the E_1

and E_2 compartments and the R_1 and R_2 compartments. (**B**) Schematic of the assumed viral kinetics model with key event times labeled. (**C**) Possible time between events for which data might be available.

Selecting Viral Kinetics and Compartmental Model Parameters

Comprehensive datasets to inform the entire viral load trajectory were lacking at the time of writing, and we therefore parameterized the model based on several key features of viral load kinetics that have been determined for SARS-CoV-2 infection. Viral loads and the timing of key events are reported with different reference points (for example, relative to the timing of symptom onset or relative to timing of exposure). However, most existing viral load time series begin after symptom onset (and are therefore from symptomatic individuals), and it is therefore difficult to corroborate assumptions for the pre-symptomatic period and asymptomatic viral trajectory. Fig. S1 depicts where in the disease course these events and parameters may be reported, which we use as a basis to choose parameter values and priors for our model.

a) Time from infection to first detectable viral load

Human infection times are never observed directly, which makes inference of viral kinetics immediately following infection challenging. Data from rhesus macaque challenge and hamster transmission models suggest that viral loads are detectable on the day of infection (*62–64*). In a cat transmission model, some secondary infected cats had detectable viral loads in oropharyngeal secretions on day two post infection (*65*). These studies suggest a very short or non-existent undetectable phase, however, animal models are likely inoculated with much higher viral loads than natural human infection, which may accelerate the time to detection.

We parameterized our viral kinetics model such that 50% of individuals had measured Ct values below the limit of detection at around day two post infection. We assumed that Ct values at the time of infection were distributed around a modal Ct value of 40 and declined thereafter, which resulted in some individuals having detectable viral loads up to five days prior to the time of typical symptom onset, which fits with studies where Ct values have been detected as early as six days pre symptom onset (*66*). For the SEEIRR compartmental model described above, we assumed a mean duration of pre-detectability ($1/\sigma$) of two days.

b) Time from infection to peak viral load

Challenge studies in rhesus macaques indicate that viral load peaks around two days after infection (*62, 65*). This is not compatible with observations from human data, which suggest that viral loads peak around the time of symptom onset, which typically occurs around five days post infection (see below). Therefore, we assumed that the Ct value reaches a minimum (log viral load peaks) on average five days post infection.

c) Incubation period for symptom onset

The time from infection to onset of symptoms has a median incubation period of 5-6 days with 99% of onsets occurring within 14 days (*67*). Although we do not explicitly model symptomatic vs. asymptomatic individuals, this parameter is useful for quantifying other key events where data are usually reported with respect to symptom onset rather than infection. Furthermore, a model comparison analysis by Ferretti et al. found that infectiousness may be tied to the timing of symptom onset rather than time since infection (for individuals who develop symptoms), which supports the parameterization of the viral kinetics curve with respect to time of symptom onset (*67, 68*).

d) Duration of growth phase and onset of infectiousness

We assumed that mean Ct decreased monotonically from the time of infection until a minimum at day five post infection, ignoring any eclipse phase ($t_{eclipse} = 0$) before the onset of viral growth. We note that the model may be parameterized such that $t_{eclipse} > 0$, but we simplified this part of the kinetics curve due to limited data and to minimize model complexity. For the SEEIRR model described above, we assumed that individuals took two days on average to transition from the pre-infectious (E_2) to the infectious (I) compartment ($1/a$). This was chosen such that infected individuals were pre-infectious for four days on average ($1/\sigma + 1/a$), earlier than the time of symptom onset, capturing the observation that a substantial proportion of transmission occurs pre-symptomatically (*15, 66, 69*). For the simpler SEIR model described above, we assumed that individuals were pre-infectious (E) for a mean of four days.

e) Time from peak viral load to symptom onset

Modelling analyses by He et al. and Ferretti et al. place the most likely time of transmission at around the time of symptom onset, suggesting that infectious viral load and symptom onset may coincide (*67, 69*). Because most viral load data are reported after symptom onset, there is limited pre-symptomatic data to assess if viral titers peak before onset (*70*). However, viral loads and PCR sensitivity appear to decrease monotonically from the time of symptom onset, suggesting that viral loads are highest just after or before the time of symptom onset (*51, 71*). We therefore parameterized our model to place peak viral load at day five post infection, coinciding with the median symptomatic incubation period.

f) Time from peak viral load to loss of infectiousness

The vast majority of studies report viral loads, culture-viable virus and model-inferred infectiousness with respect to symptom onset, which may occur after peak viral loads. We therefore quantify the timing of infectiousness with respect to symptom onset, described below.

g) Time from peak viral load to loss of detectable viral load

As in f), we quantify waning rates with respect to symptom onset as described below due to the lack of viral load data reported with respect to peak viral load.

h) Time from symptom onset to loss of infectiousness

The relationship between viral load and infectiousness are currently unknown for SARS-CoV-2. However, there are currently two main proxies used to estimate time-varying infectiousness: model-based results using generation intervals of known infector-infectee pairs (*67, 69*), and the ability to culture live virus from swab samples taken each day post symptom onset (*67, 72*). Model-based analyses have estimated substantial pre-symptomatic transmission probability, suggesting that individuals are infectious before symptom onset (*67, 69*). For virus culture data, a systematic review found that viable virus is unlikely to be cultured from samples taken more than nine days post symptom onset and another study found that higher viral loads are correlated with probability of live virus culture, though we note that lack of viral culture has not been shown to indicate lack of infectiousness (*15, 70, 73–75*).

For the SEIR transmission model, a rapid scoping review suggested using a median six-day infectious period for asymptomatic infections and median 9.5 days for symptomatic infections (*15, 69*). However, because much of the data we analyze here were collected from populations under transmission-reducing interventions where the infectious period would likely be shorter, we assumed that the observed mean $(1/\gamma)$ infectious period was four days in both the SEIR and SEEIRR models. We note that we do not fix this parameter but estimate it alongside other parameters using a strong prior, and therefore do not exclude the possibility of longer or shorter infectious periods.

i) Time from symptom onset to loss of detectable viral load

Waning of viral loads occurs following the onset of symptoms, with the median time from onset to loss of detectability in upper respiratory tract samples of approximately two weeks, though some studies suggest a more prolonged waning rate and greater persistence in lower respiratory tract and sputum samples (*15, 44, 53, 71, 76*). One patient has been reported detectable at day 83 post symptom onset, indicating that some individuals remain PCR positive long after symptom onset (*77*). We therefore parameterized our model to capture both a median time from symptom onset to detectability loss of around two weeks and the possibility for some individuals remaining detectable for much longer. In the SEEIRR model, we used a point estimate for $1/\omega$ (recovery period) of 11 days, corresponding to an average loss of detectability at 19 days post infection (14 days post typical symptom onset).

Informing the Viral Kinetics Model

The literature summarized in *Supplementary Material: Selecting Viral Kinetics Parameters* informed parameters for the pre-viral peak phase and provided a basis with which to fully parameterize the model. To more formally parameterize the viral kinetics model, we used a least-squares optimization framework to obtain parameter point estimates that gave rise to viral kinetics with the following constraints: the proportion of individuals that are detectable on each day post symptom onset declines in line with existing data (*71*); the lower 99th percentile of possible Ct values at peak viral load is in line with either the lowest observed Ct value in our Brigham & Women's Hospital dataset or from the long-term care facilities data; and the lowest 99th percentile of Ct values 30 days post infection is in line with either a Ct value of 32 in the BWH analyses or 30 in the long-term care facilities analyses. From the calibration steps we obtained estimates for mean peak viral load that were broadly in line with the range reported in other studies (up to 10^9 viral RNA copies per ml) (*52*, *78*). We used these point estimates to derive informative priors on key model parameters, as described in Table S1. The resulting distribution of Ct values and detectable proportions at each day a after infection are shown in Fig. S2. These parameters are used as fixed values in Fig. 1.

We note that for the pre-peak phase, solving the model from an initial Ct value at the LOD on day 0 allows for a proportion of individuals to have detectable viral loads up to five days prior to peak viral load (the typical time of symptom onset), which captures individuals who may have longer incubation periods and therefore detectable viral loads for many days before onset.

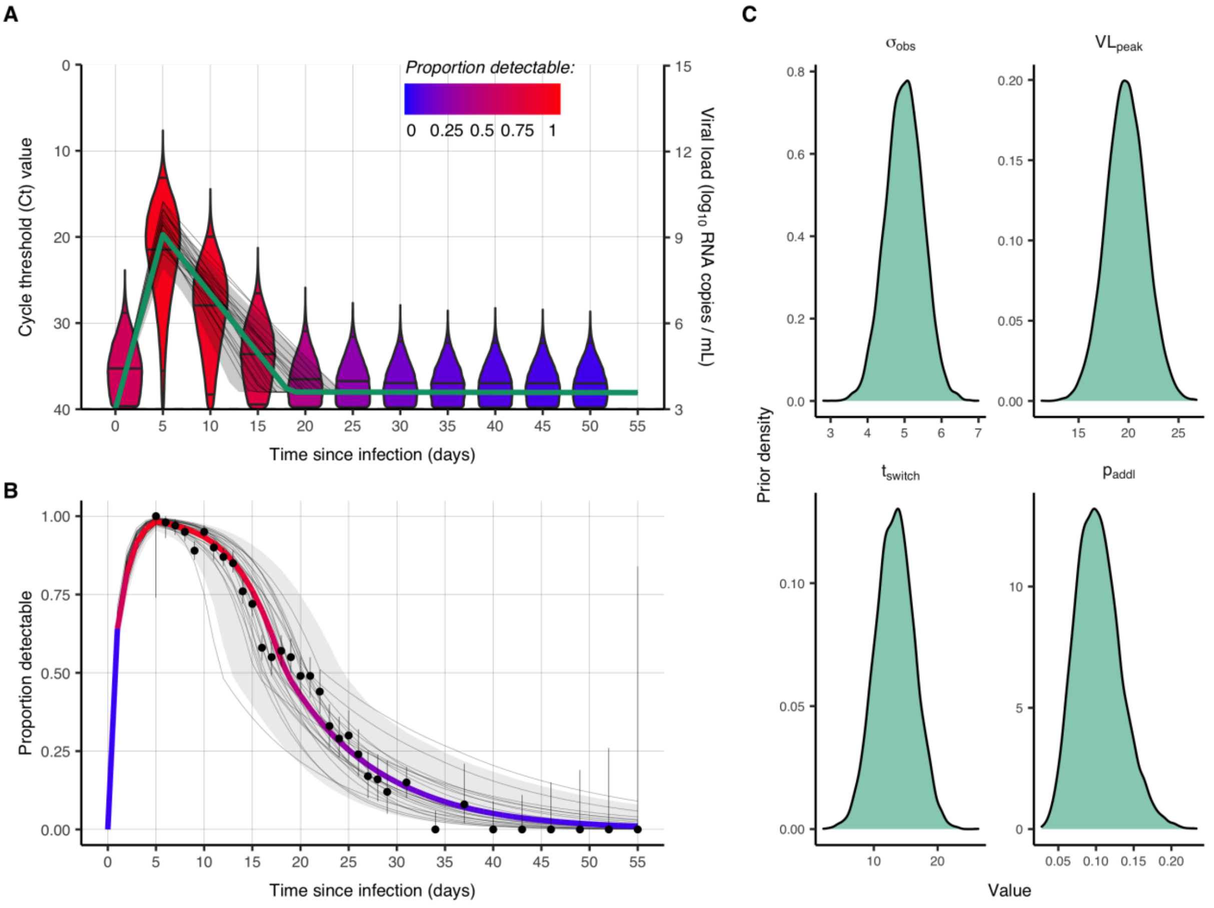

Fig. S2. Fitted viral kinetics model for cycle threshold (Ct) values, assumed loss of detectability over time, and assumed priors on model parameters for analyses of data from Brigham & Women's Hospital, Massachusetts. These assumptions underpin the analyses presented here, but we note that more up to date estimates or alternative data, which may demonstrate different kinetics, can be used where appropriate to improve the accuracy of estimated epidemic trajectories (*79*). (**A**) Solid green line shows the modal viral load trajectory over time since infection. Faint grey lines show trajectories from prior draws, and faint grey ribbon shows 95% quantiles. Violin plots show the distribution of detectable Ct values for each five-day increment post infection using the maximum *a priori* trajectory (green line). Violins are colored by the proportion of Ct values above the limit of detection. (**B**) Least-squares based fit (colored line) to the proportion of individuals detectable in upper respiratory tract samples on each day post symptom onset (assuming an incubation period of five days) from Borremans *et al.* (*71*). Black dots and lines show proportion positive and 95% confidence intervals. Faint grey lines show proportion detectable over time from prior draws, and faint grey ribbon shows 95% quantiles. (**C**) Assumed prior densities for unknown model parameters. Model parameters and priors, as well as those used for the analyses of data from long-term care facilities, are shown in Table S1.

Parametric Models for Fitting Cross-Sectional Viral Load Data

Exponential Growth Model

Assume that over the A_{max} days prior to testing day t, the daily incidence grows (or declines) exponentially at rate r so that $\pi_{t-a} = \pi_0 \exp[r(t-a)]$. The exponential growth rate β is thus the logarithm of the daily growth rate in incidence over the days $t - A_{max}$ to $t-1$. Larger values of β indicate faster growth of new infections and a value of 0 indicates no increase or decrease in the number of new infections each day. For positive values of r, the doubling time (in days) for new infections is given by $\frac{\log 2}{r}$. This model may be a reasonable approximation in the early stages of an outbreak, when the number of susceptible individuals is large compared to the number of infections (*20*). However, constant exponential growth is a poor assumption for an epidemic process over longer periods of time. We therefore set A_{max}, and equivalently t_{max} under the nomenclature in the section *Epidemic Transmission Models,* to be 35 days, chosen to compromise between the duration that most individuals remain PCR positive post infection without assuming constant exponential growth for too long a period.

Fitting this likelihood, and ignoring the nuisance parameter π_0, gives:

$$\mathcal{L}(X_1, \dots, X_n \mid r) \propto \prod_{i=1}^{n} \left[\left(\sum_{a=1}^{A_{max}} p_a(X_i)\, \phi_a \exp[r(t-a)] \right)^{I(X_i < C_{LOD})} \left(1 - \sum_{a=1}^{A_{max}} \phi_a \exp[r(t-a)] \right)^{I(X_i \geq C_{LOD})} \right],$$

if detectable and undetectable Ct values are recorded. If only detectable Ct values are recorded, then the parametric likelihood is given by:

$$\mathcal{L}(X_1, \dots, X_n \mid r) \propto \frac{\prod_{i=1}^{n} \left[\sum_{a=1}^{A_{max}} p_a(X_i)\, \phi_a \exp[r(t-a)] \right]}{\left(\sum_{a=1}^{A_{max}} \phi_a \exp[r(t-a)] \right)^n}.$$

To incorporate uncertainty in the distribution of viral loads on each day after infection, we construct a Bayesian framework for estimation and inference. In this paper, we use a normally-distributed prior for r with mean 0 and standard deviation 0.25, fitted using a Markov chain Monte Carlo (MCMC) algorithm to obtain the posterior distribution. Note that $r = 0.1$ corresponds to a doubling time for new infections of approximately one week. The prior distributions for the parameters for the Ct kinetics model are given in Table S1 and described above.

This method also results in posterior distributions for the parameters for the Ct kinetics model. These are nuisance parameters for estimation of the epidemic trajectory but may be useful in improving the priors for future use of this model.

SEIR Model

As an alternative parameterization of the likelihood, we also use the SEIR compartmental model described above (see *Epidemic Transmission Model*). We generate Ct values for all exposed, infectious, and recovered individuals when they are sampled based on the Ct value model described above. This model is most appropriate for a relatively closed population, where the outbreak is initiated by one or several initial infections and no transmission-reducing measures are taken over the time period studied. We use this model for the long-term care facility outbreaks observed early in the SARS-CoV-2 pandemic in Massachusetts and the single cross-section analyses using the BWH data.

The prior distributions for the parameters of the SEIR model are given in Table S1. For any set of parameter values, the daily probability of infection, π_t, is determined by finding the incidence of newly exposed individuals and the likelihood found using the appropriate nonparametric likelihood equation (either including all samples or only samples with a detectable Ct value) given above. Posterior distributions for the SEIR model parameters are obtained using MCMC fitting, along with posterior distributions for the Ct kinetics model parameters. From the posterior estimates of the SEIR model parameters, posterior distributions can also be found for related model features, such as R_t, as of the testing day t (*20, 32*). Under this model, the parameter A_{max} is simply taken as the number of days between testing day t and day 0 of the modeled period. For example, in the long-term care facility analyses, day 0 was assumed to be February 1, 2020, and the epidemic seed date, t_0, was estimated.

Gaussian Process Model

We present results from the Gaussian process model in terms of the posterior distribution for π_t, the daily incidence rate values. For identifiability, the sum of π_t over all possible values of t is set equal to 1; thus, the resulting estimates should be considered the relative probability of infection on each day, relative to the set of possible days. When only positive PCR test results are included in the inference, we estimate π_t directly. When negative PCR tests are also included, we multiply π_t by an estimated scaling factor between 0 and 1, which is the absolute probability of infection from the entire incidence curve. Xu *et al.* describe and illustrate the use of the GP as a prior distribution for nonparametric inference on incidence rates for various infectious disease settings (*34*). Under this model, π_t is considered for each day of the simulation, and there is no separate epidemic seed time parameter as in the SEIR model. To account for the fact that Ct values sampled on day t will have arisen from infections up to A_{max} days prior to the first sampling time, we set A_{max} to 35 days. This captures most individuals who were infected prior to the first sampling date and are still detectable. Therefore, when fitting the Gaussian process model to multiple rounds of test data, a daily probability of infection parameter π_t is estimated for each day in the time period $T_{min} - A_{max}$ to T_{max}, where T_{min} and T_{max} are the earliest and latest testing days respectively. Finally, we note that the Gaussian process hyperparameters, ν and ρ, were fixed in the model fitting. Although it is theoretically possible to jointly estimate ν and ρ (with

exponentially distributed priors), we found that not constraining these parameters led to very poorly converged chains when fitting the model.

Epidemic Seed Time Priors

External information on the epidemiological context can be used to further constrain estimated epidemic trajectories. When fitting the SEIR model to the single cross-sections of Ct values from the BWH in Massachusetts, we are estimating the dynamics of a single epidemic peak that precedes the observation time. We therefore placed uniform priors on the epidemic seed time, t_0, to reflect prior knowledge of the start of the two epidemic growth phases. Specifically, for samples taken prior to June 1, 2020, we assumed that the seed time was between February 1, 2020, and April 1, 2020. For samples taken between June 1, 2020, and August 1, 2020, we assume that the seed time is unknown between February 1, 2020, and two weeks prior to the sample time. This captures the assumption that we do not know if infections during this time are dominated by the decline phase of the first wave or the growth phase of the second. If the sample time was after August 1, 2020, we assume that the seed time of the second wave is unknown between June 1, 2020, and 2 weeks prior to the sample time. This ensures that we are estimating incidence based on the second wave for these later samples.

As a sensitivity analysis, we instead assumed that the epidemic seed time was unknown between 2020-02-01 and the sampling date for all sampling times (Fig. S14D,E). Estimated growth rates were very similar, though the posterior densities for the first four weeks were wider when the epidemic seed times were less constrained. Some of the posterior estimates were bimodal (Fig. S15), where the same Ct distribution could be explained as resulting from very recent and fast epidemic growth (most high Ct values are from the upswing of a recent infection) or from the downswing of a declining epidemic (most high Ct values are from the clearance phase). Although our method is able to accurately estimate these bimodal posterior distributions, it is important to interpret them alongside the epidemiological context. Without suitable priors, estimates that are mathematically correct are not necessarily epidemiologically plausible.

Comparison of Analysis Methods

Simulated Long-Term Care Facility Outbreaks

To ensure that our method provides accurate estimates of the epidemic trajectory using either only detectable Ct values or all PCR test results, we performed extensive simulation-recovery experiments using synthetic closed populations undergoing stochastic SEIR epidemics (Figs. S9, S10A). We evaluated the accuracy and precision of growth rate estimates from the SEIR and exponential growth models fitted to cross-sectional Ct values observed during epidemic growth, decline and around the peak (Fig. S10B–D). The SEIR model consistently provided unbiased, constrained daily growth rate estimates at all three timepoints when all sample results

were used. When only the distribution of detectable Ct values was used, estimates during the growth and decline phases were accurate but exhibited wide credible intervals, whereas estimates during the peak phase were slightly biased upward. Estimates of the average growth rate from the exponential model were consistent using either all or only positive samples—slightly higher than the daily growth rates from the SEIR model during the peak and decline phases, reflecting the drop in daily growth rate relative to the past average as the epidemic begins to decline.

We also found that increasing the simulated population size from 300 to 5000 did not change the accuracy of our estimates and had only a modest impact on 95% credible interval widths (Fig. S11). Similarly, using progressively less informative priors for the viral kinetics parameters did not change the accuracy of the inferred growth rates, but did increase the uncertainty in the growth rate estimates (Fig. S12).

Symptom-based Reporting

Reported case counts arise from a model of symptom onset and reporting delays following infection. We assume a log-normal incubation period with mean of log(5) days and standard deviation 0.418, as estimated by Lauer et al. (*68*). In the simulation, we assumed that 35% of individuals were symptomatic, with an incubation period drawn from this log-normal distribution. Each symptomatic individual then has some probability of being tested with a delay between symptom onset and test report date, where this probability may vary by day of the outbreak. Three scenarios are considered: flat testing (fixed probability of testing of 10%); increasing testing rates (a linear increase in probability of testing from 10% 36 days prior to the analysis day to 20% one day prior to the analysis day); decreasing testing rates (a linear decrease in probability of testing from 10% 36 days prior to the analysis day to 1% one day prior to the analysis day).

R_t is estimated from these simulated data using the R package *EpiNow2* (*32*, *33*), which is available at https://github.com/epiforecasts/EpiNow2. This requires the following inputs:

1. Time series data for the number of newly confirmed cases per day.
2. A specified incubation period distribution, giving the distribution of delays between infection and symptom onset.
3. A specified reporting delay distribution, giving the distribution of delays between symptom onset and case confirmation.
4. Priors on the generation interval distribution, specifying the mean and standard deviation of the times between infection in infector-infectee pairs.

For the reporting delay distribution, we assumed a discretized gamma distribution with shape and scale parameters of 5 and 2 respectively (mean of 2.5 days and standard deviation of 1.12 days). For the generation interval, in our simulations using the SEIR model, the mean generation interval is given by $T_c = 1/\sigma + 1/\gamma$, where σ and γ are the inverse of the mean incubation and infectious periods in days, respectively. The variance of the generation interval

distribution is given by $Var = 2\left(\frac{T_c}{2}\right)^2$. Therefore, T_c =8 days, with standard deviation of 5.66 days. In *EpiNow2*, normal priors were placed on these quantities with standard deviations of 3 for both.

Random Surveillance

For the surveillance Ct sample analyses, we use random sampling where each individual has a 0.3% probability of being tested at some point in the outbreak. We consider a single testing day (sampling 0.3% of the population), two testing days one week apart (sampling 0.15% of the population on each day), and three testing days each one week apart. For the three testing days, we consider scenarios where the probability of being sampled on any one of those days is flat at approximately 0.1%, rising from 0.05% to 0.10% to 0.15%, or falling from 0.15% to 0.10% to 0.05% across the three days. In all of these settings, approximately 3000 total tests are conducted. Fig. 3B plots the estimates using these methods from 100 simulations at two time points in the epidemic: one before the peak incidence (true $R_t > 1$) and one after the peak incidence (true $R_t < 1$).

Fig. S13 compares results from the surveillance sample analyses using Ct values to those using positivity rates alone for total surveillance sample sizes ranging from 100 to 3000. The sample sizes are split among one to three test days, with multiple test days one week apart, with the three test days further incorporating rising testing, falling testing, or flat testing across the three days. Fig. S13A displays the median and interquartile range (IQR) of the median posterior R_t estimates from 100 samples, Fig. S13B displays the mean squared error of the median posterior R_t estimate from 100 samples, Fig. S13C displays the mean width of the 95% credible intervals for the R_t estimate from 100 samples, and Fig. S13D displays the proportion of the 100 samples in which the 95% credible interval lies entirely above 1 for a growing epidemic or below 1 for a declining epidemic.

Comparison to Model Fitting Using Only Percent Positive Tests

We also consider estimation of the SEIR model using only the positivity rates (i.e., proportion of tests with Ct values below 40) from the surveillance samples. In this case, we fit the SEIR model to the prevalence estimate assuming that PCR positivity occurs if and only if an individual is in the infectious compartment. As in the rest of the model fitting, we used the same MCMC framework and priors, but used a binomial likelihood for the number of observed positive and negative test results conditional on the SEIR model trajectory.

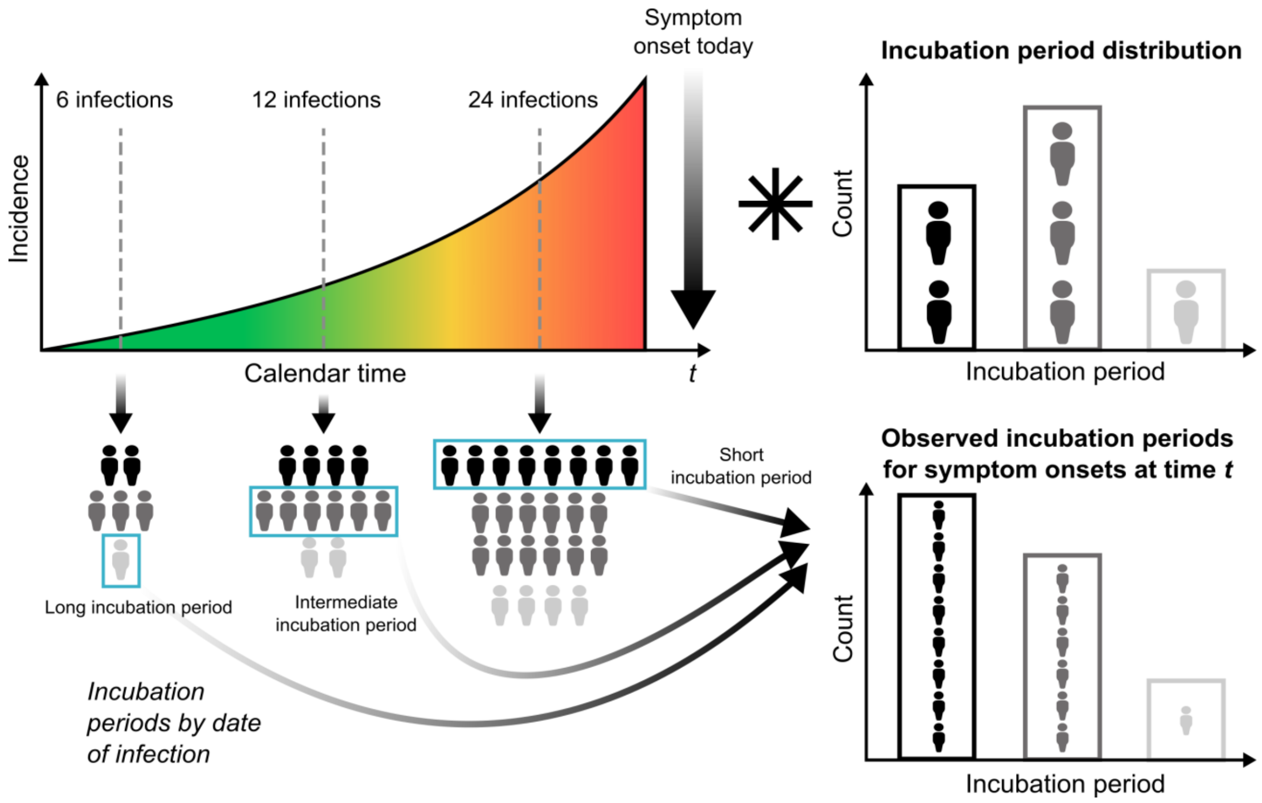

Fig. S3. The incubation period distribution from individuals sampled on a particular day under symptom-based surveillance will vary depending on the epidemic growth rate. Under any surveillance strategy, the distribution of observed delays between infection date and test report date is a convolution of the infection incidence curve and the reporting delay distribution (time from infection to reporting a test result), which includes the incubation period distribution in symptom-based surveillance (*32*). Through this convolution, the distribution of delays between infection date and report date will change depending on the epidemic growth rate even if the underlying incubation period and testing delay distributions remain unchanged. This figure illustrates how the distribution of observed incubation periods *conditional on time of symptom onset* will differ from the incubation period distribution *conditional on time of infection*. From the time of infection, the distribution of incubation periods will always follow the incubation period shown in the top right of the figure (i.e., how long do I expect to wait until symptom onset?). However, when measuring this distribution for all individuals with symptom onset on day *t* (i.e., given that I had symptom onset today, when was I infected?), the distribution of observed incubation periods will be a mixture of infections on previous days with long or short incubation period periods: individuals could have symptom onset from an older infection with a long incubation period (grey figure) or from a recent infection with a short incubation period (black figures). The incidence curve determines the weights of the contribution of recent infections with short incubation periods and of older infections with long incubation periods. Growing incidence results in a higher proportion recent infections, whereas declining incidence results in a higher proportion of older infections. Note, the star symbol represents the mathematical operation of convolution.

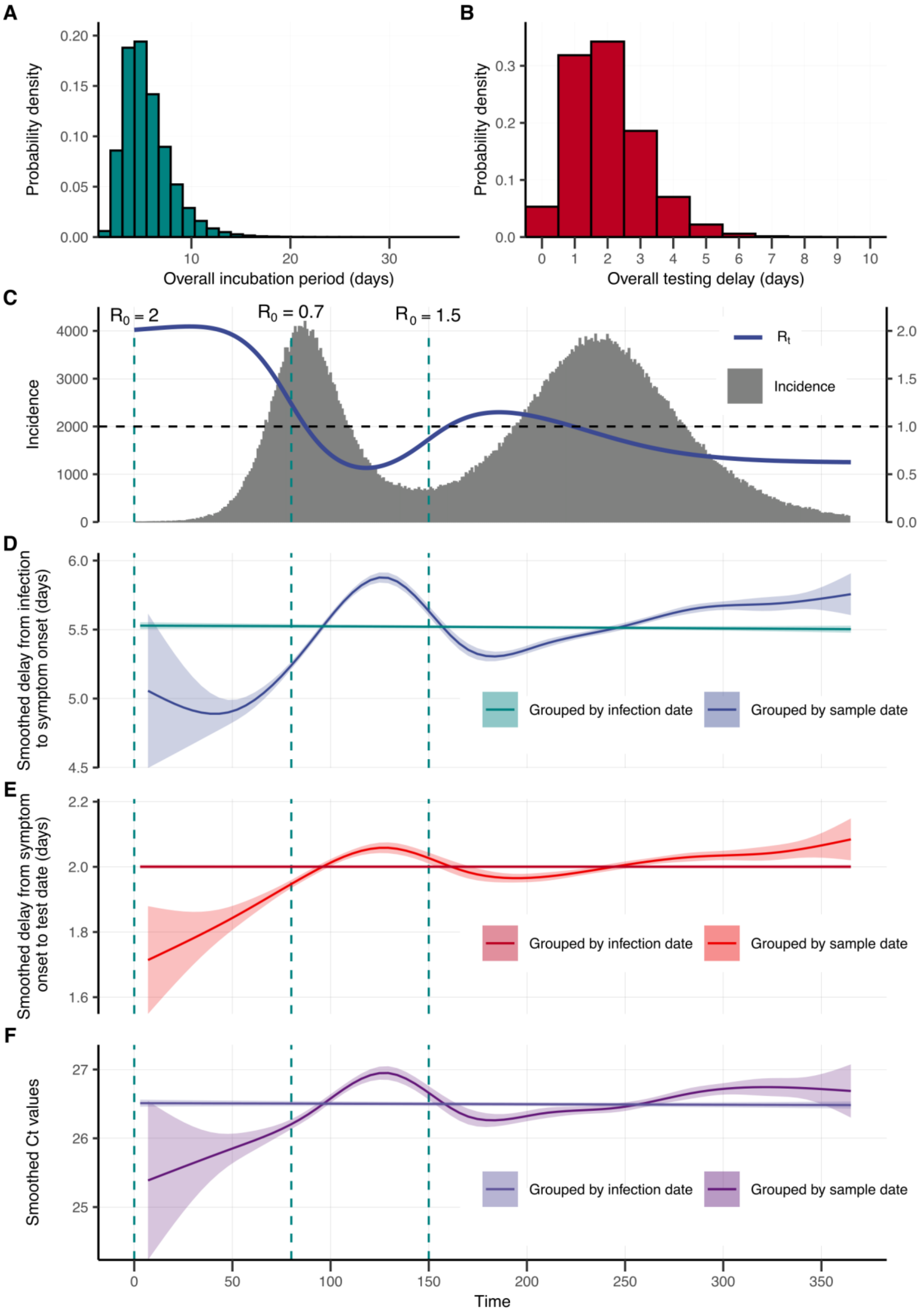
A
B
C
D
E
F
Probability density
Overall incubation period (days)
Overall testing delay (days)
$R_0 = 2$
$R_0 = 0.7$
$R_0 = 1.5$
R_t
Incidence
Smoothed delay from infection to symptom onset (days)
Grouped by infection date
Grouped by sample date
Smoothed delay from symptom onset to test date (days)
Smoothed Ct values
Time

Fig. S4. The Ct value distribution is expected to change over an epidemic under symptom-based surveillance. To demonstrate this point, a stochastic SEIR model was simulated with switch points at days 80 and 150, representing changes in R_0 driven by implementation of non-pharmaceutical interventions or increasing virus transmissibility. R_0 changes were interpolated to smooth transitions between epidemic stages. Symptom-based surveillance was simulated assuming that each infected individual had a 35% change of becoming symptomatic and subsequently tested, had an incubation period drawn from a log-normal distribution shown in (**A**) (*66*), and had a delay between onset and testing drawn from a gamma distribution with mean 2.5 days and variance 1.25 days shown in (**B**). Each sampled individual has a simulated Ct value based on the time since infection (note, independent of the incubation period). Simulated incidence and effective reproductive number, R_t are shown in (**C**), where the vertical blue lines show timing of R_0 switch points. (**D**), (**E**) and (**F**) show LOESS smoothing splines with 95% confidence intervals fitted to simulated (**D**) incubation periods, (**E**) testing delays over time and (**F**) Ct values at time of sampling. Crucially, splines were fit to data stratified by either *date of infection* or by *date of sample collection*. These simulations illustrate two key factors which might lead to changing Ct values over time when observed under symptom-based surveillance: 1) if the expected viral load in a symptomatic person declines with time since infection even after conditioning on time since symptom onset, then the changing distribution of delays between infection and symptom onset shown in (**D**) will influence measured Ct values; 2) the expected viral load in a test sample declines with time from symptom onset to sample collection, therefore measured Ct values will depend on the epidemic trajectory as the distribution of delays between symptom onset and sample collection is influenced by the epidemic growth rate as shown in (**E**). Note that the relationship shown in (**F**) will depend on whether viral load trajectories are anchored to time of infection, as assumed for the results shown in this simulation, or to time of symptom onset (e.g., if a symptom onset is caused by reaching a high viral load). If viral loads among symptomatic individuals are only dependent on the time since symptom onset and not the time since infection, then the relationship shown in (**D**) will no longer have an impact on the Ct distribution. However, the epidemic trajectory will still have a weak influence on the Ct distribution mediated by its convolution with the testing delay distribution as shown in (**E**).

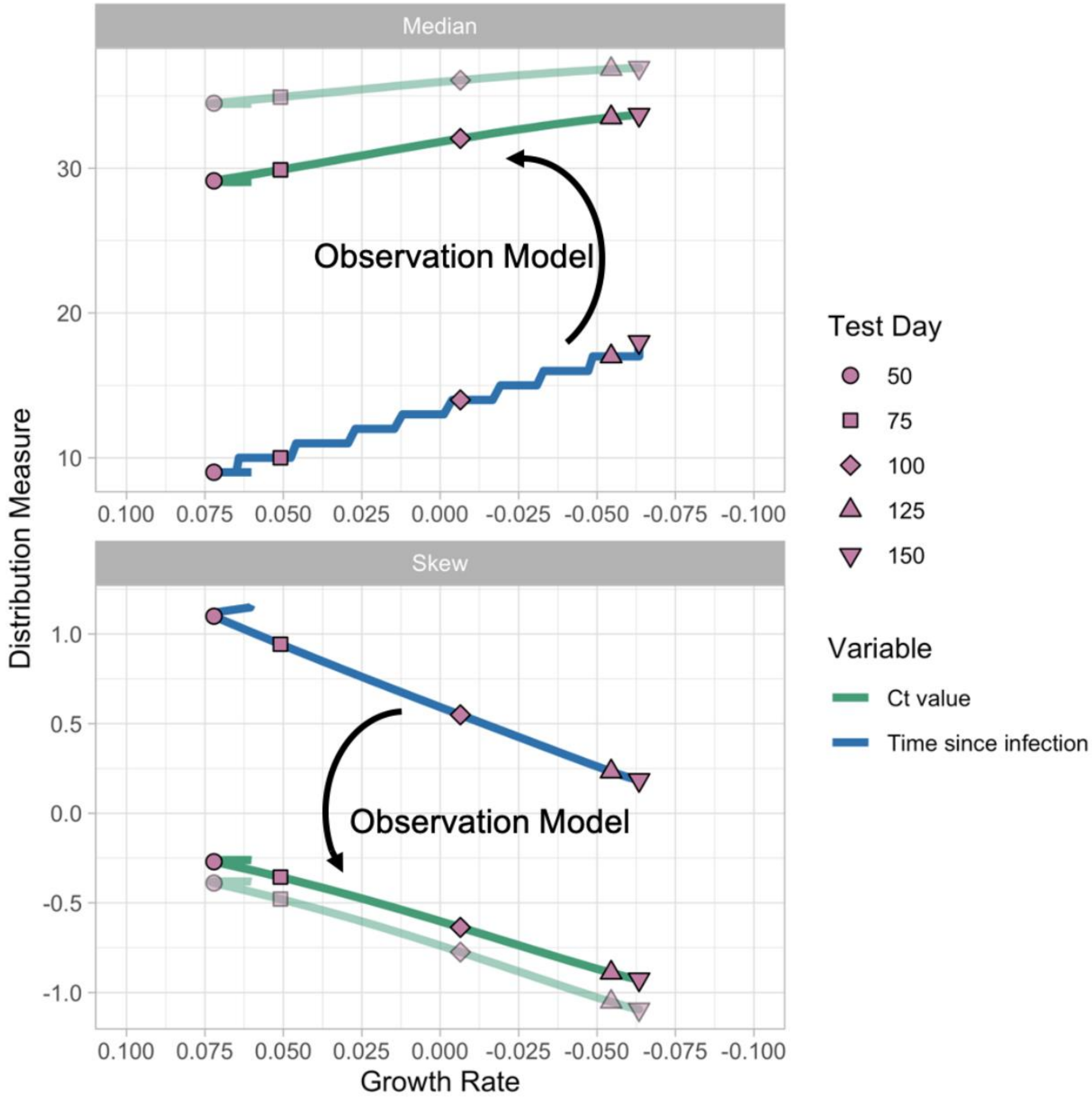

Fig. S5. Distributional properties of times since infection (blue line) correlate with epidemic growth rate, which can be observed using cycle threshold (Ct) values as a proxy with a properly calibrated observation model (green line). Median (**Top**) and skewness (**Bottom**) of the time since infection (blue line) and observed Ct value (green lines) by average 35-day growth rate from the simulated susceptible-exposed-infectious-recovered (SEIR) model. The two green lines represent two possible observation models (e.g., from different RT-qPCR machines, protocols, or swab locations).

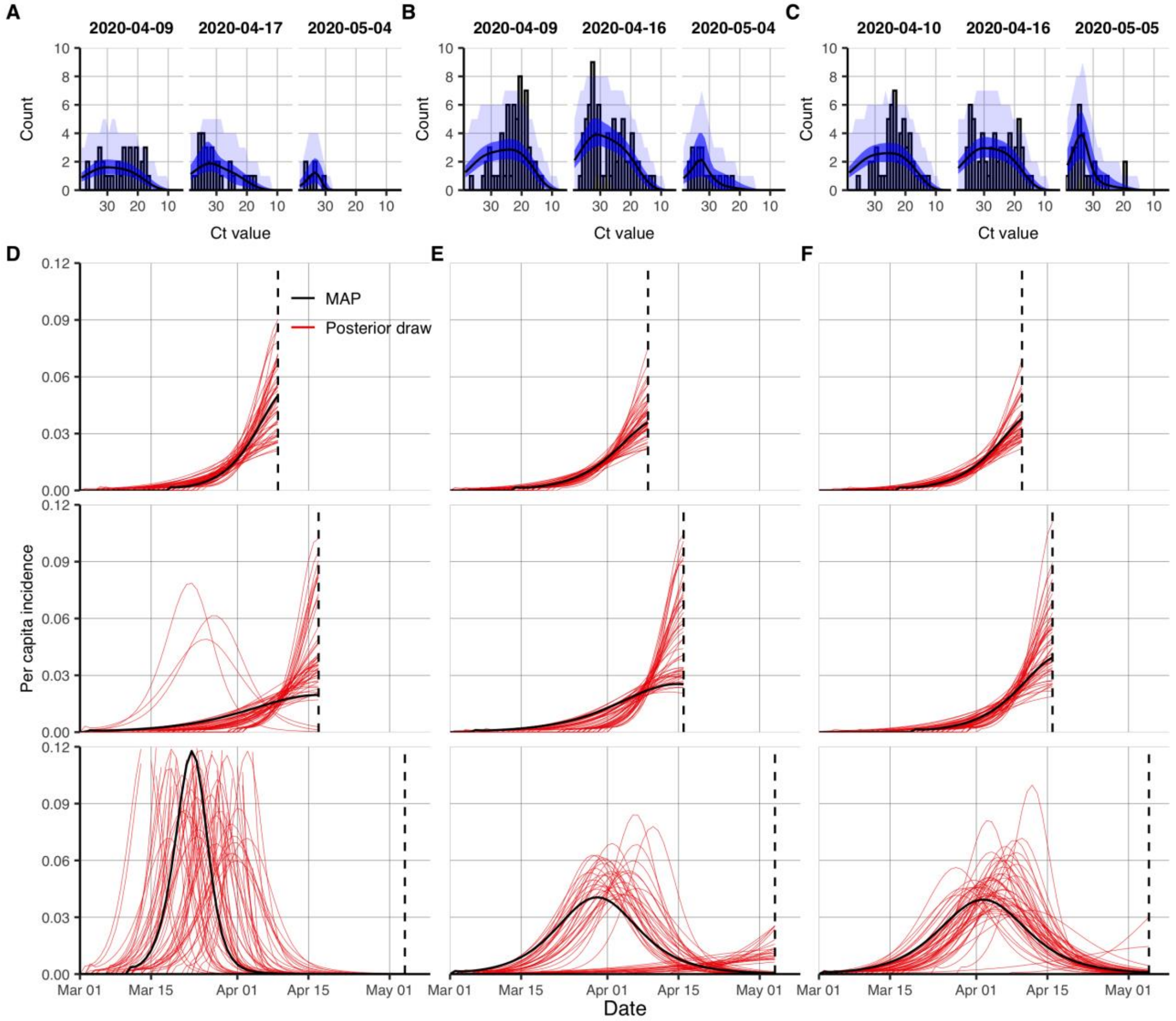

Fig. S6. Single cross-sectional distributions of observed cycle threshold (Ct) values used to reconstruct epidemic trajectories in three additional Massachusetts long-term care facilities. (Top) Model-predicted Ct distributions (blue) fitted to the observed Ct values (grey bars) from that cross-sectional sample for the second (**A**), third (**B**) and fourth (**C**) long-term care facilities. Posterior median (black line) and 95% credible intervals for the expected Ct distribution (dark blue ribbon), and 95% prediction intervals based on simulated observations (light blue ribbon). (**Bottom**) Each panel shows results from fitting the Ct-based SEIR model separately to cross-sections of virologic data from the second (**D**), third (**E**) and fourth (**F**) long-term care facilities. Shown are random posterior samples (red lines) and the maximum posterior probability trajectory (black line) for the incidence curve. Note that we use a parallel tempering algorithm, so conflicting trajectories are an accurate representation of the multi-modal posterior.

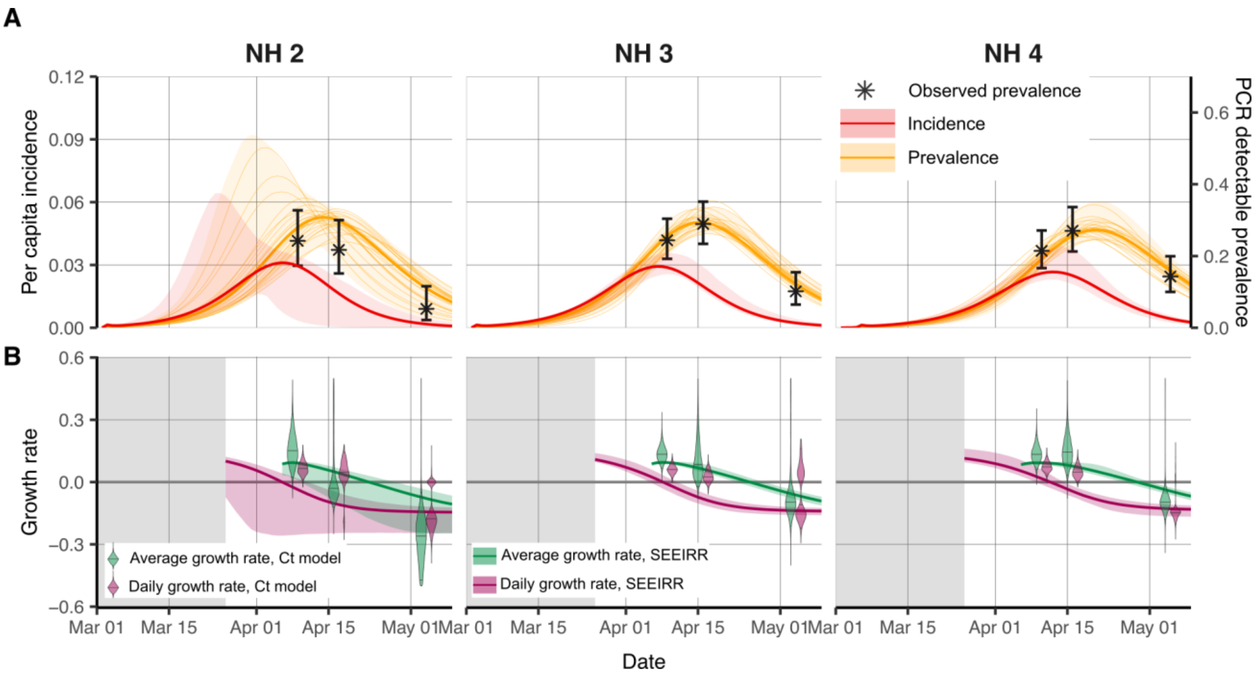

Fig. S7. Comparison of growth rate estimates from the Ct-based methods and from the SEEIRR compartmental model fitted to point prevalence in three additional Massachusetts long-term care facilities. (**A**) Posterior distribution of prevalence (orange lines and shaded ribbon) from the SEEIRR model fit to point prevalence at three sampling times for each facility and posterior distribution of daily per capita incidence (red line and shaded ribbon) from the same model. Black error bars show 95% binomial confidence intervals on PCR positive prevalence. (**B**) 35-day (green) and 1-day (pink) average growth rates from fitting the Ct-based SEIR model to the three time points (violin plots) or from fitting the SEEIRR model to point prevalence (lines and shaded ribbons) for each facility. The three facilities included here are different from the one shown in Fig. 2.

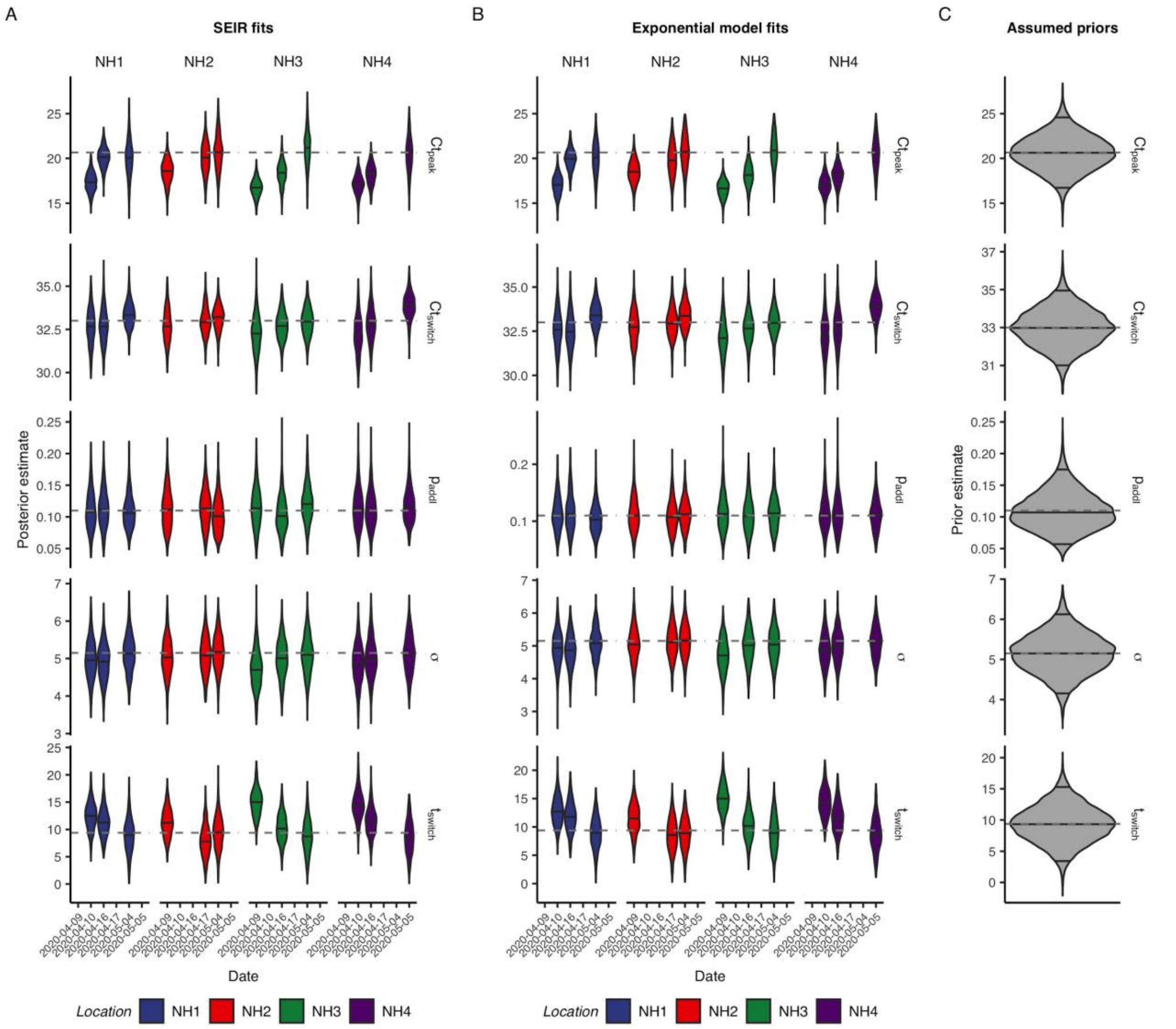

Fig. S8. Prior and posterior distributions of parameters for models fit to observed cycle threshold (Ct) values from four Massachusetts long-term care facilities. (**A** and **B**) Posterior distributions of model parameters from the SEIR model (**A**) and exponential growth model (**B**). (**C**) Prior distributions of model parameters used in both models. NH1 is the facility shown in Fig. 2; the others are shown in Figs. S6 and S7.

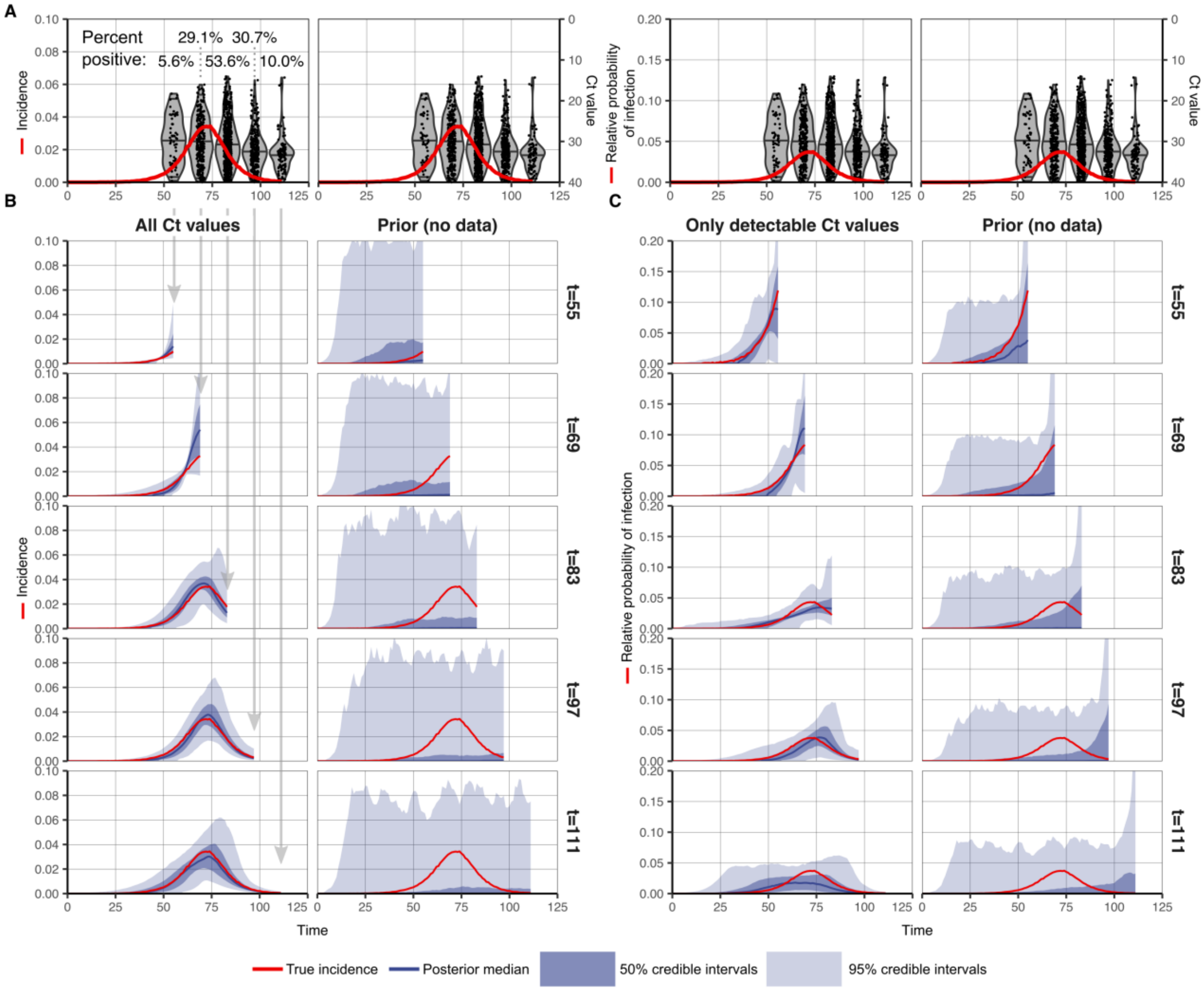

Fig. S9. A single cross-sectional sample of Ct values can be used to accurately re-estimate the true incidence curve at all stages of an epidemic. (**A**) Each plot shows the same simulated dataset, assuming that PCR tests were performed on a different set of 1000 randomly selected individuals on days 55, 69, 83, 97 and 111 of a simulated stochastic SEIR epidemic in a population of 100,000 individuals. Ct values at each sample time were simulated conditional on the time since infection using the Ct model. Text labels show the percentage of samples testing positive. (**B**) For each cross section, an SEIR curve was fitted using the simulated Ct values, including undetectable samples (i.e., the proportion positive). The blue ribbon shows the 95% (light) and 50% (dark) credible intervals for the estimated daily incidence curve. The blue line shows the posterior median daily incidence. The simulation truth is shown in red. The left-hand column shows the results from using all Ct values, and the right-hand column shows the results from excluding the data in the fitting (using prior information only). (**C**) As in (**B**), but using only detectable Ct values (i.e., there is no information on the proportion positive). Note that the y-axis shows the relative rather than absolute probability of infection; each estimated incidence curve must sum to one in the detectable-only estimates, as all samples are positive and therefore all included individuals are assumed have been infected.

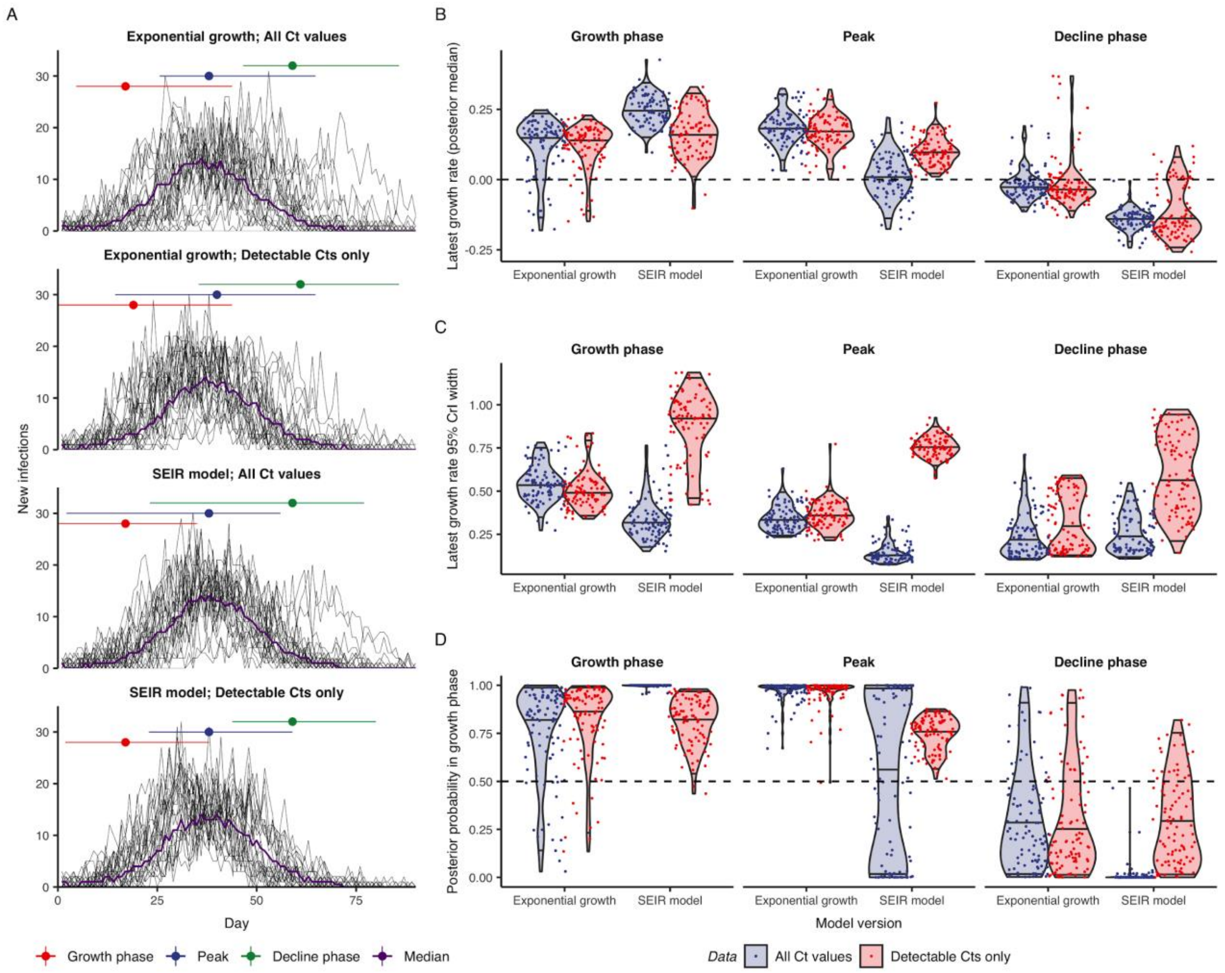

Fig. S10. In simulations representing the Massachusetts long-term care facilities, the cycle threshold (Ct) value-based methods for epidemic trajectory estimation recover the simulation parameters for exponential growth and SEIR models with or without negative test results. (**A**) Distribution across 100 simulations of median posterior estimates of daily infection incidence by model used, stratified by the growth, peak, and decline phases of the epidemic. (**B**–**D**) Distribution across 100 simulations of median posterior estimates for growth rate (**B**), widths of 95% credible intervals for growth rate (**C**), and posterior probability that the growth rate is greater than 0 (**D**) at the end of the three phases of the epidemic for each of four models.

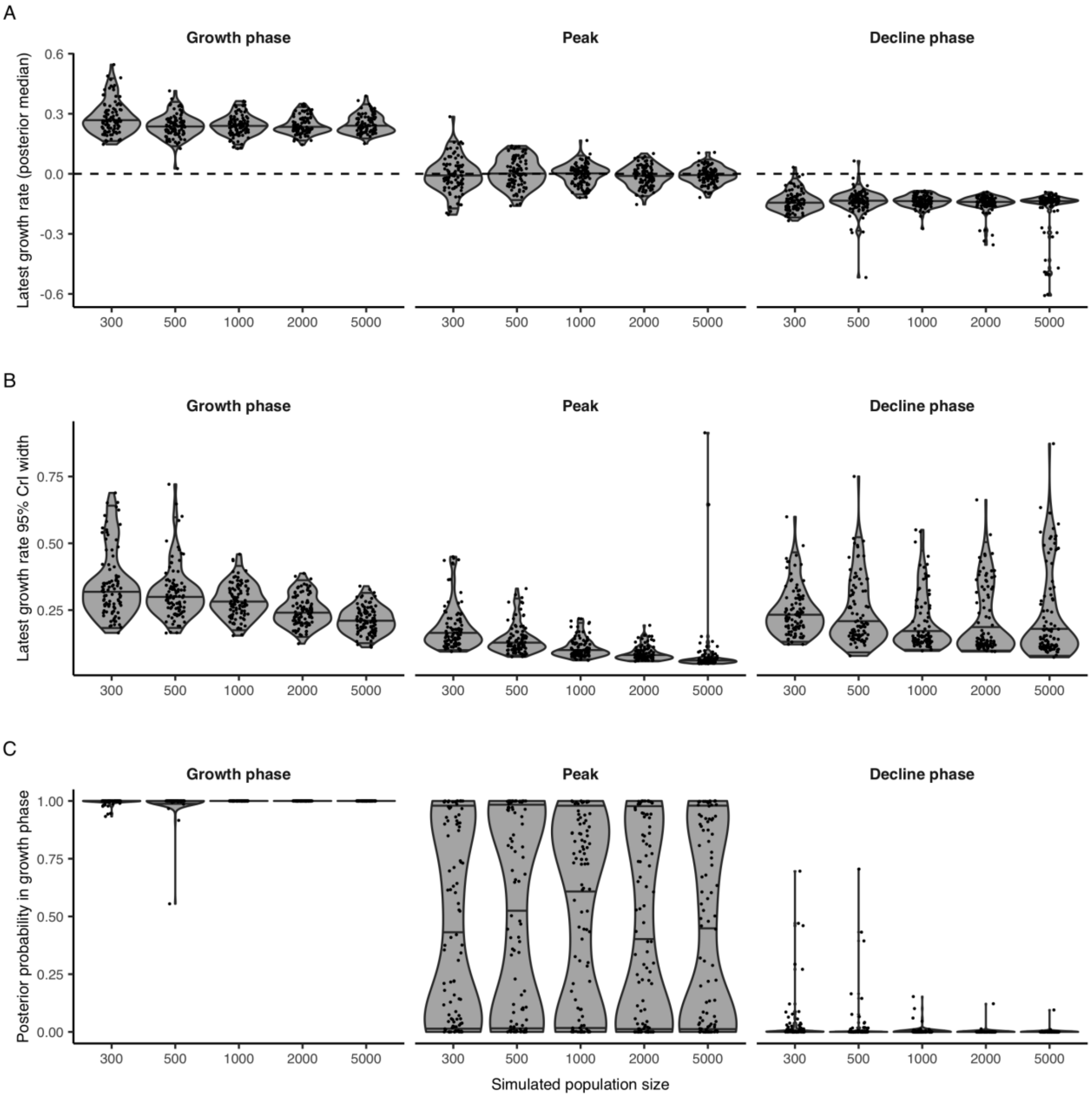

Fig. S11. Varying population size has little impact on the performance of the cycle threshold (Ct) value-based methods for epidemic trajectory estimation using an SEIR model with negative test results in recovering simulation parameters similar to the Massachusetts long-term care facilities parameters. Distribution across 100 simulations of median posterior estimates of growth rate (**A**), widths of 95% credible intervals of growth rate (**B**), and posterior probability that the growth rate is greater than 0 (**C**) at the end of the three phases of the epidemic by population size. Results shown are as in Fig. S10B–D, with population size as an additional x-axis.

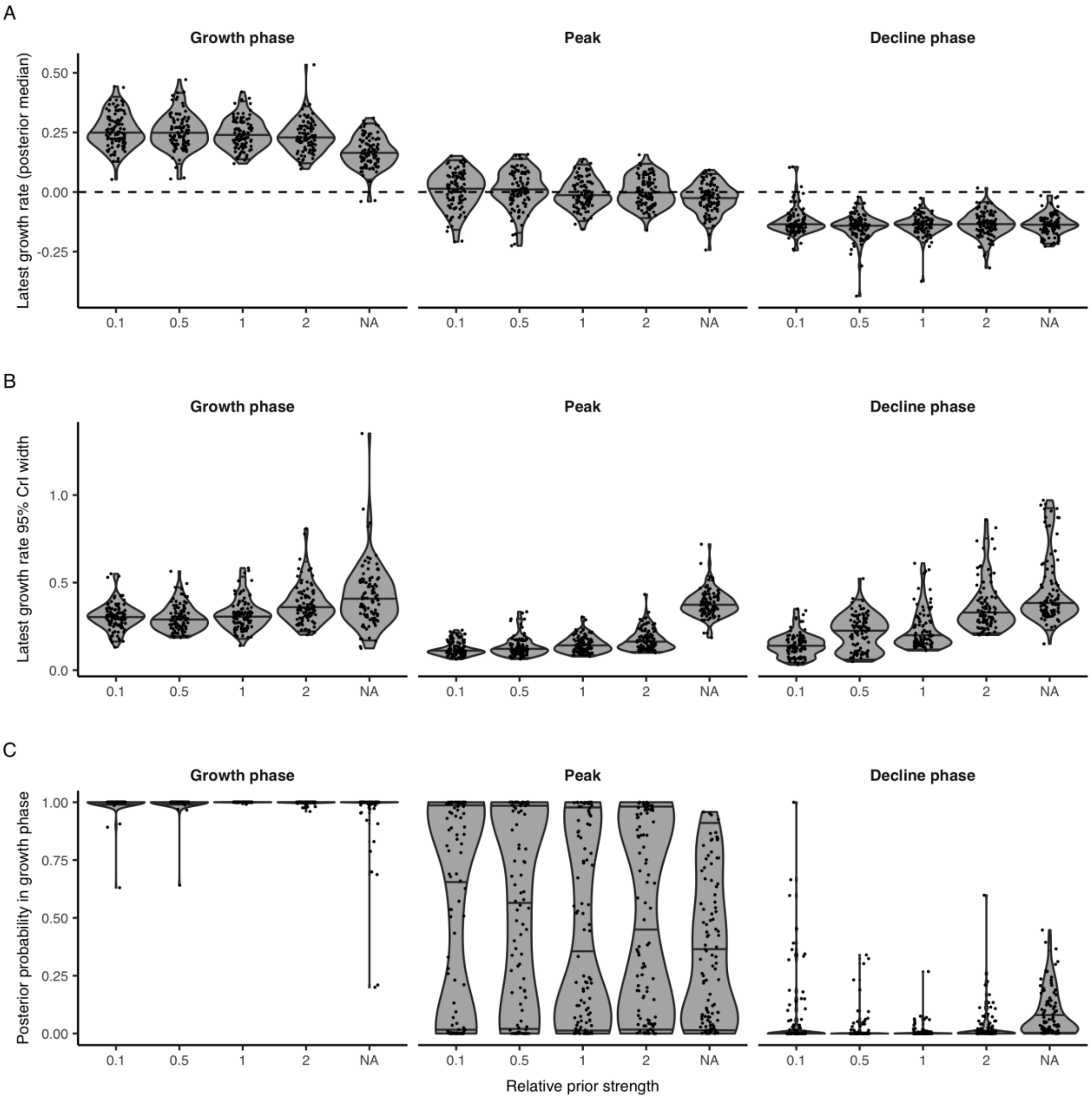

Fig. S12. Varying the relative strength of the prior distributions has a modest impact on the performance of the cycle threshold (Ct) value-based methods for epidemic trajectory estimation using an SEIR model with negative test results in recovering simulation parameters representing the Massachusetts long-term care facilities parameters. Distribution across 100 simulations of median posterior estimates of growth rate (**A**), widths of 95% credible intervals of growth rate (**B**), and posterior probability that the growth rate is greater than 0 (**C**) at the end of the three phases of the epidemic by relative strength of prior distribution. The rightmost estimates in each plot (labeled NA) indicate the use of uniform priors on all model parameters. Results shown are as in Fig. S10B–D, with relative prior strength as an additional x-axis. Note that

less informative priors are likely to have a greater impact on credible interval widths when only detectable Ct values are included, as information in the proportion positive is lost.

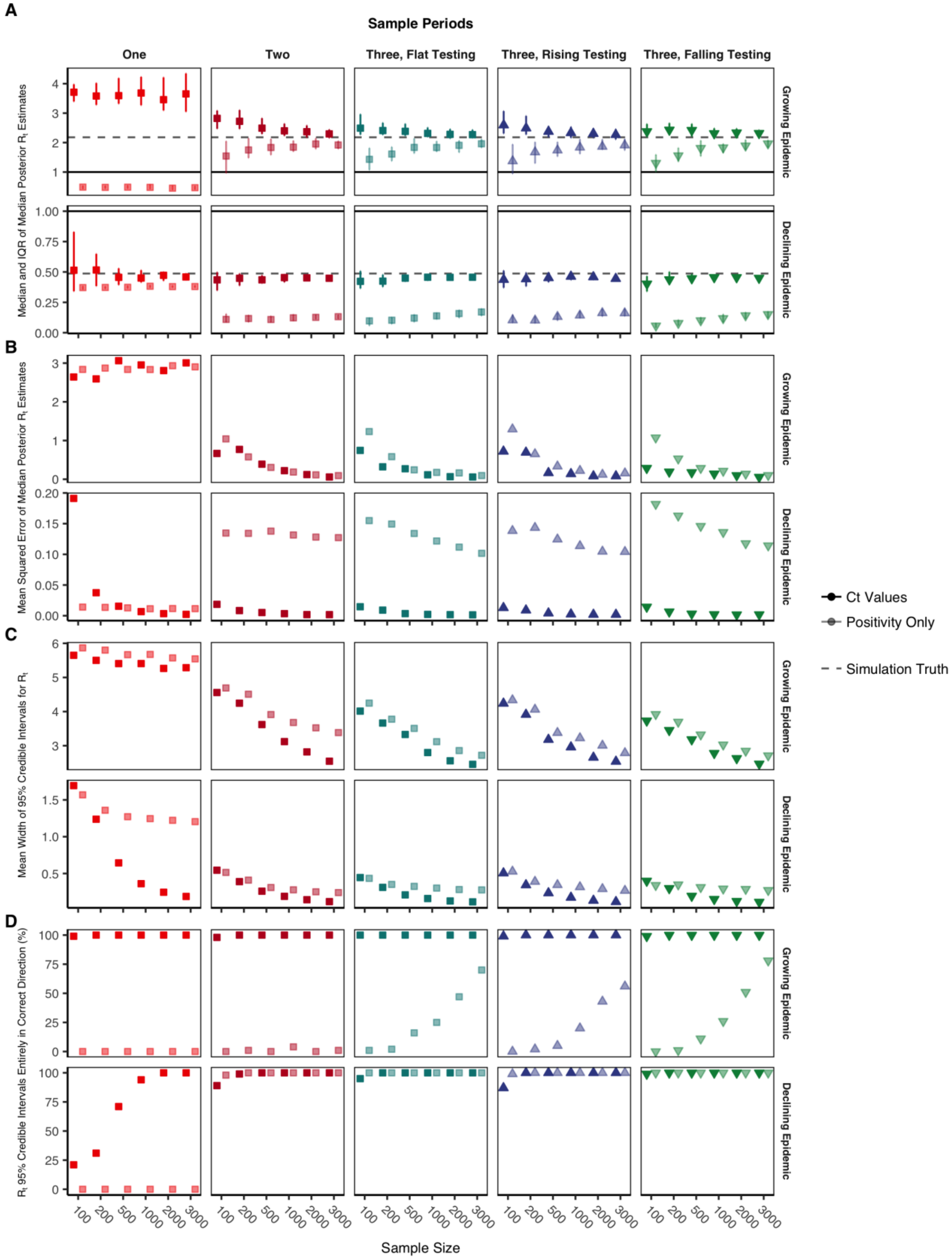

Fig. S13. Incorporating Ct values outperforms positivity rates alone in estimating susceptible-exposed-infectious-recovered (SEIR) model from surveillance samples, across

sample sizes and number of test days. Results across 100 simulations for median and interquartile range of median posterior R_t estimates (**A**), mean squared error of median posterior R_t estimates (**B**), mean width of 95% credible interval for R_t estimate (**C**), and percent of 95% credible intervals which lie entirely above 1 for a growing epidemic or below 1 for a declining epidemic (**D**). The total sample size across all testing days is given on the x-axis. For multiple testing days, the days are one week apart, with the same final day of testing in all cases: epidemic day 60 in the growing epidemic panels and epidemic day 88 in the declining epidemic panels. For each sample size, the leftmost point uses the Ct values, fitting an SEIR model with a population-level viral kinetics model and the rightmost (semi-transparent) point uses the positivity rate of the tests alone, fitting an SEIR model assuming PCR positivity is equivalent to infectiousness.

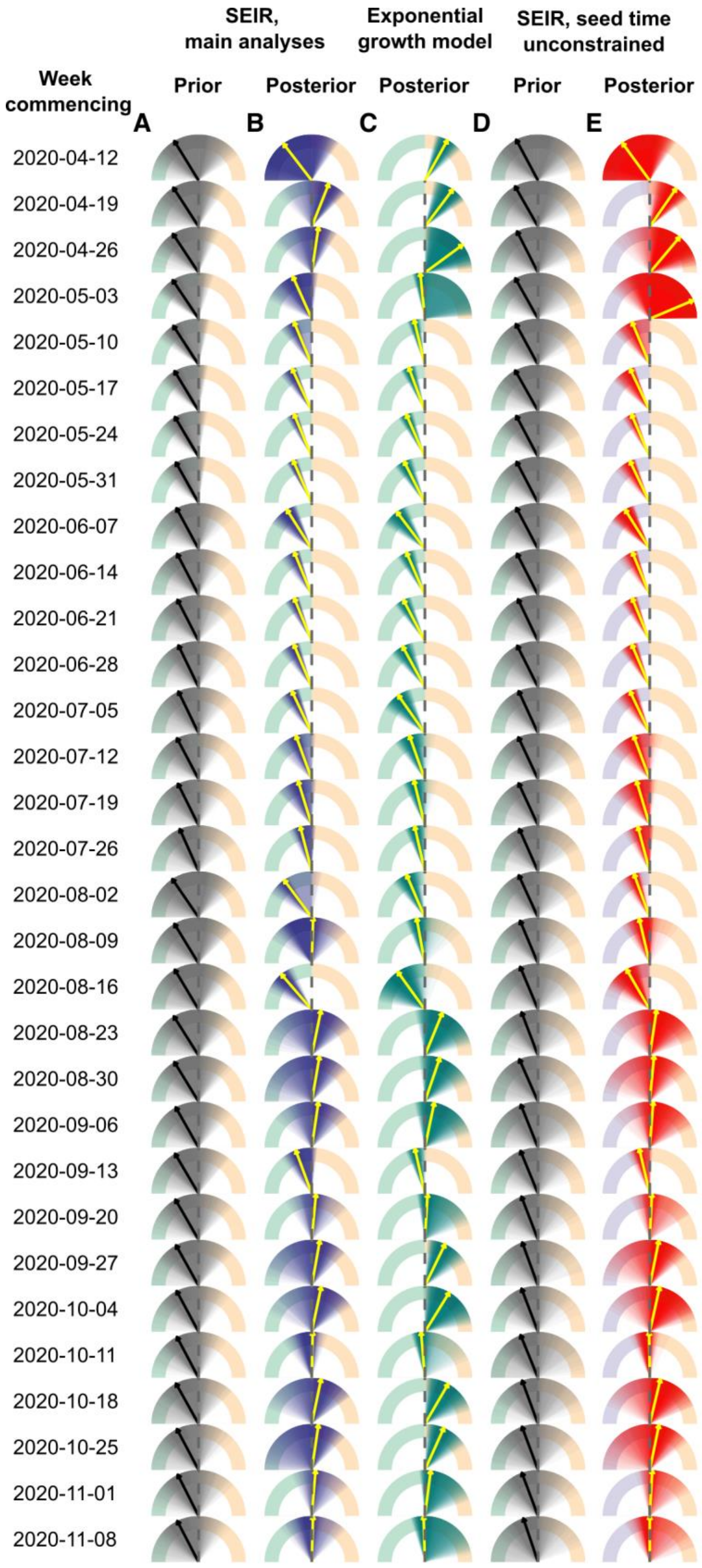
SEIR, main analyses
Exponential growth model
SEIR, seed time unconstrained
Week commencing
Prior
Posterior
Posterior
Prior
Posterior
A
B
C
D
E
2020-04-12
2020-04-19
2020-04-26
2020-05-03
2020-05-10
2020-05-17
2020-05-24
2020-05-31
2020-06-07
2020-06-14
2020-06-21
2020-06-28
2020-07-05
2020-07-12
2020-07-19
2020-07-26
2020-08-02
2020-08-09
2020-08-16
2020-08-23
2020-08-30
2020-09-06
2020-09-13
2020-09-20
2020-09-27
2020-10-04
2020-10-11
2020-10-18
2020-10-25
2020-11-01
2020-11-08

Fig. S14. All single cross-section growth rate estimates using Ct values from Brigham & Women's Hospital, Massachusetts. Dials show estimated growth rates using data collected in the week shown to the left of the plot. Dials range from -0.5 to +0.5 (i.e., left of the vertical axis indicates epidemic decline). Shaded regions show posterior or prior densities. Yellow arrows show posterior medians and black arrows show prior medians. Note that wide estimates represent true multimodal posterior distributions, as these Ct distributions could be generated either at the start of fast growth or in decline phases (see Fig. S15). (**A**) Prior daily growth rates (grey) from the SEIR model assuming constraints on the epidemic seed time. (**B**) Posterior daily growth rates (blue) from the SEIR model assuming constraints on the epidemic seed time. (**C**) Posterior 35-day average growth rates (green) from the exponential growth model. (**D**) Prior daily growth rates (grey) from the SEIR model, assuming no constraints on the epidemic seed time. (**E**) Posterior daily growth rates (red) from the SEIR model, assuming no constraints on the epidemic seed time.

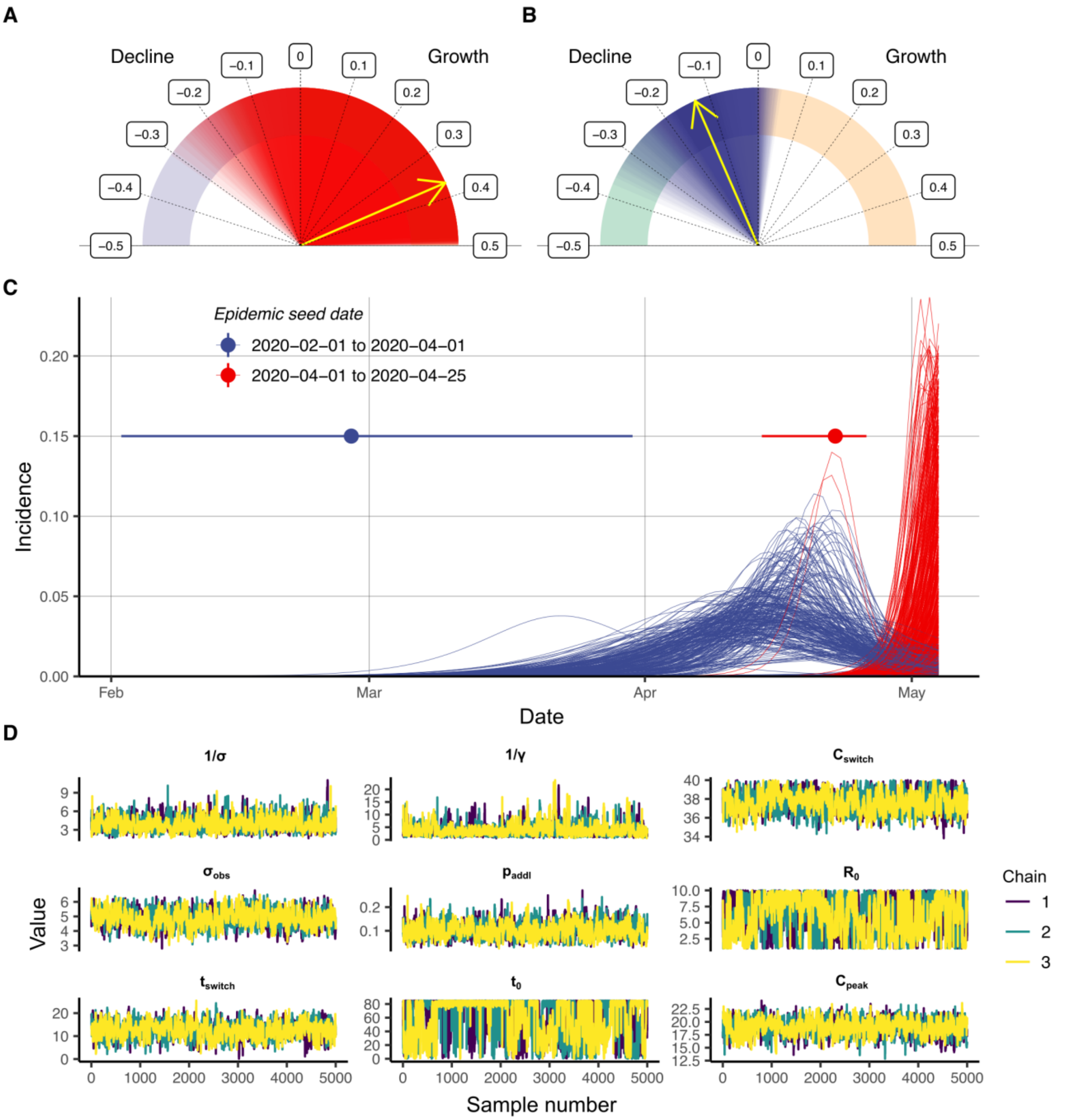

Fig. S15. **Estimated epidemic trajectories from single cross sectional Ct distributions can be multimodal.** (**A**) Estimated daily growth rate using single cross-sections of Ct values from samples obtained in the week commencing 2020-05-03 and the SEIR model with no constraints on the epidemic seed time. Red shaded region shows posterior densities, yellow arrows show posterior medians. (**B**) As in (**A**) but placing a prior constraint on the epidemic seed time to be prior to April 1, 2020. (**C**) Epidemic trajectory using BWH data sampled in the week commencing May 3, 2020. Each line is a randomly drawn posterior sample for the SEIR incidence curve using the posteriors shown in (**A**). Lines are colored based on whether the seed time was before April 1,

2020, or after. Point range plots show 95% credible intervals and posterior median on the epidemic seed time depending on whether the seed time was before or after April 1, 2020, demonstrating a multimodal posterior. (**D**) MCMC trace plots for estimated parameters underpinning the trajectories in (**C**). Trace plots demonstrate good convergence, but clear multi-modality for R_0 and t_0; the same data can be explained with either high R_0 and late t_0, or low R_0 and early t_0.

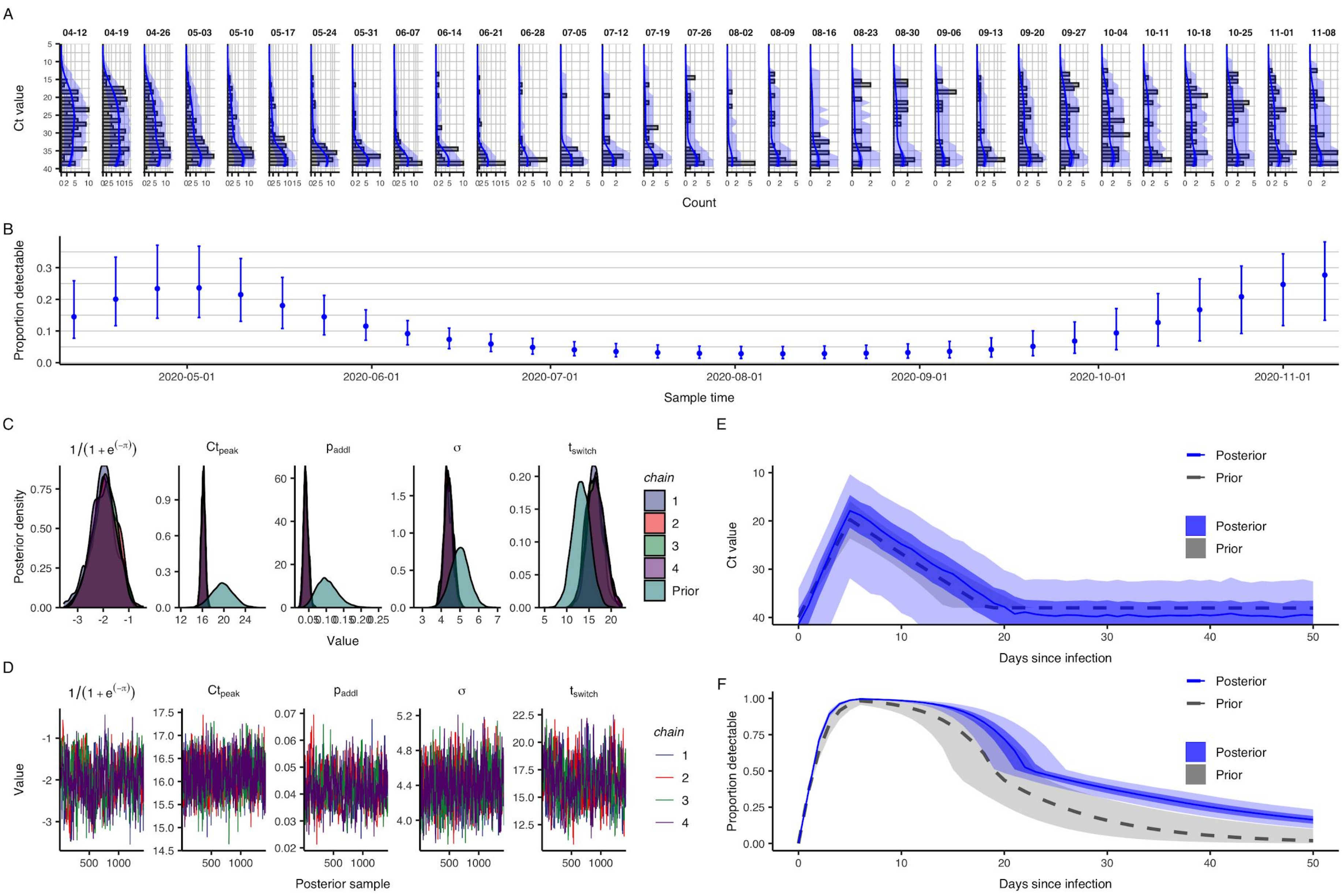

A
B
C
D
E
F
Ct value
Count
Proportion detectable
Sample time
2020-05-01
2020-06-01
2020-07-01
2020-08-01
2020-09-01
2020-10-01
2020-11-01
Posterior density
Value
Posterior sample
chain
Prior
Posterior
Days since infection

Fig. S16. Data and results summaries for estimation of epidemic trajectory in Massachusetts shown in Fig. 4. (**A**) Histogram of observed cycle threshold (Ct) values by weekly sample. Blue line shows posterior median blue ribbon shows 95% credible intervals (CrI) for the expected Ct distribution. (**B**) Model-predicted proportion of samples with detectable Ct value by weekly sample. Point-range plot shows posterior median and 95% CrI. (**C**) Prior vs. posterior density of model parameters from Gaussian process model fit to observed Ct value data. (**D**) Markov chain Monte Carlo trace plots for model parameters fit to observed Ct value data. (**E, F**) Prior vs. posterior density of population-level observed Ct value model, mean Ct value (**E**) and probability of having a detectable Ct value (**F**), by days since infection fit to observed Ct value data.

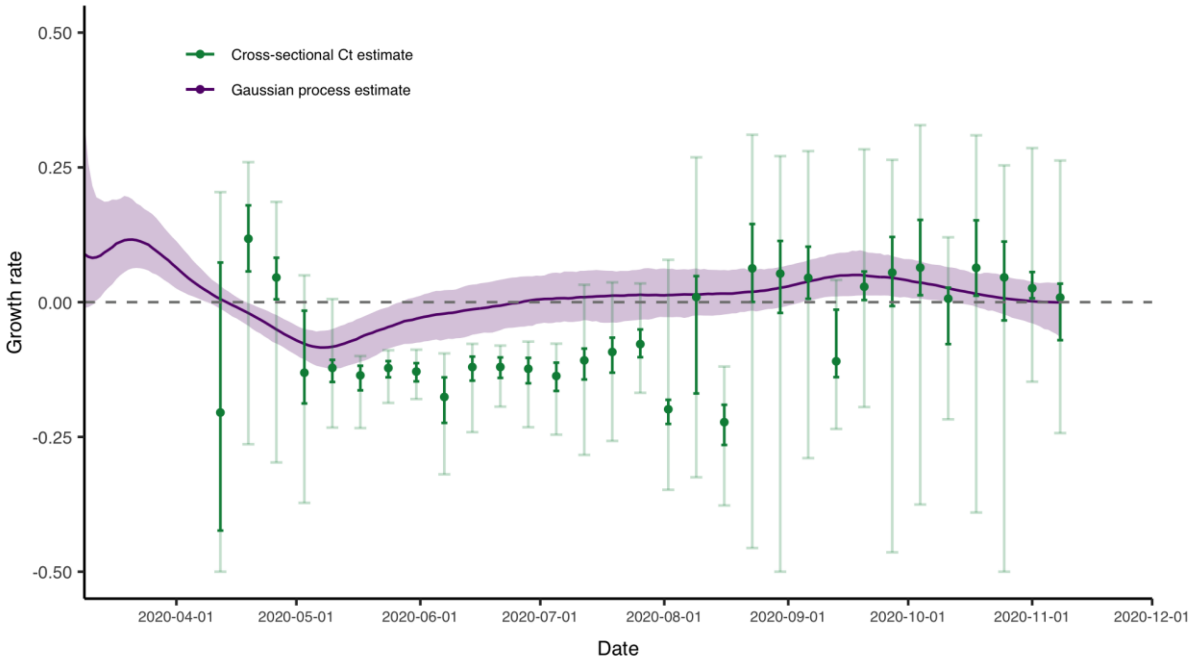

Fig. S17. Growth rates estimated using cycle threshold (Ct) values from the Brigham & Women's Hospital through fitting either the SEIR model to each single-cross section separately or from fitting the Gaussian process model to the full dataset. Green points and error bars show posterior medians (points), 50% (dark green error bar) and 95% (light green error bar) credible intervals (CrI) for the estimated growth rate on the date shown, obtained by fitting the SEIR model to each single cross-section of Ct values obtained in that week. The purple line and ribbon show the posterior median and 95% CrI for the estimated daily growth rate obtained by fitting the Gaussian process model to all of the cross sections simultaneously as in Fig. 4.

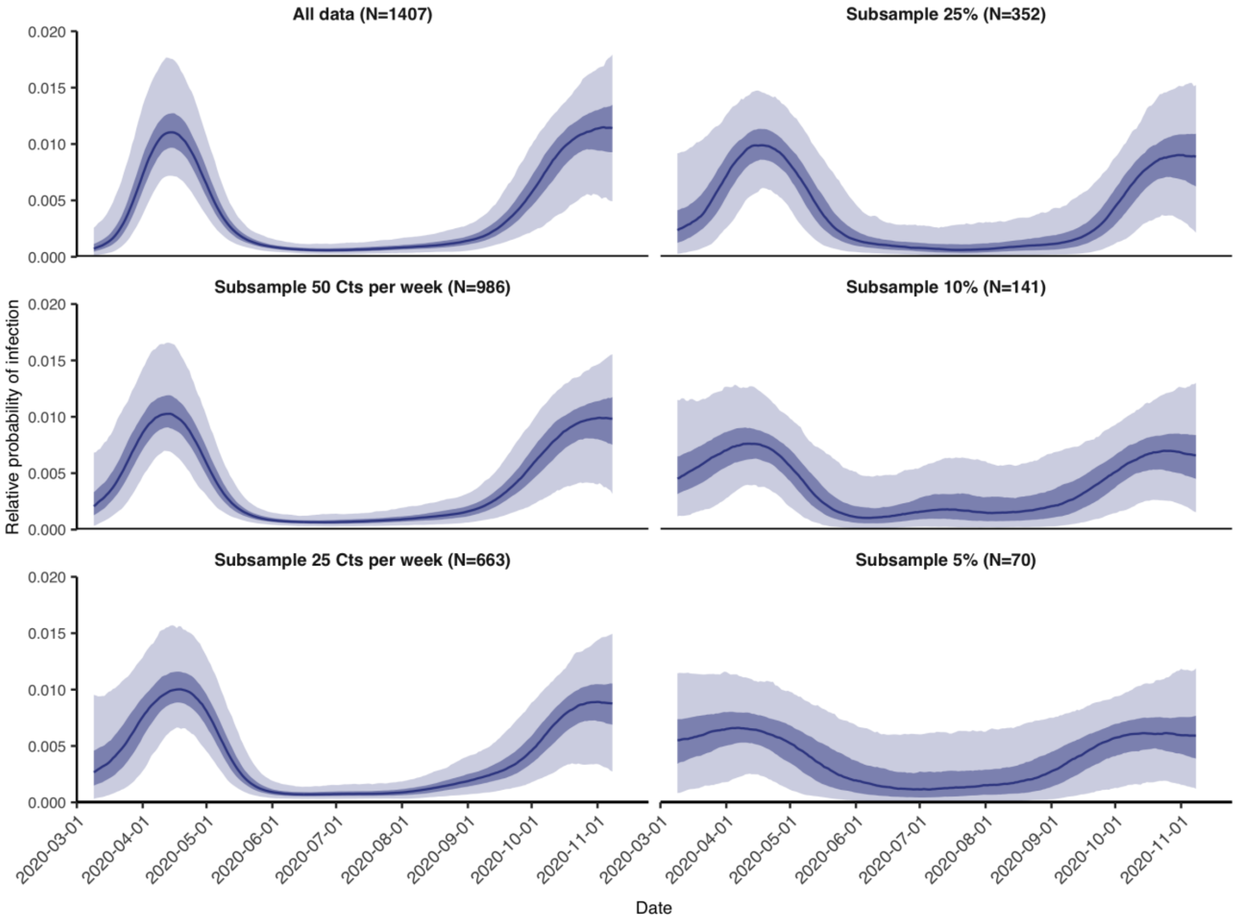

Fig. S18. Estimated epidemic trajectory remained from re-fitting the Gaussian process model to progressively smaller subsamples of the full Brigham & Women's Hospital dataset. Shown are the posterior median (blue line), 50% (dark blue ribbon) and 95% (light blue ribbon) credible interval (CrI) estimates for the scaled daily probability of infection obtained through fitting the Gaussian process model to the entire dataset. Note that the y-axis shows the scaled rather than absolute probability of infection, as all recorded samples were positive and therefore the cumulative probability of infection for the estimated incidence curve must sum to one (all samples in the dataset were from infections). Each panel shows the result from fitting the model to a random subsample of the full dataset after: i) subsampling to give at most 50 cycle threshold (Ct) values per week, with no subsampling in weeks with fewer than 51 Ct values; ii) subsampling to give at most 25 Ct values per week, with no subsampling in weeks with fewer than 26 Ct values; iii) randomly subsampling 25% of the full dataset; iv) randomly subsampling 10% of the full dataset; v) randomly subsampling 5% of the full dataset.

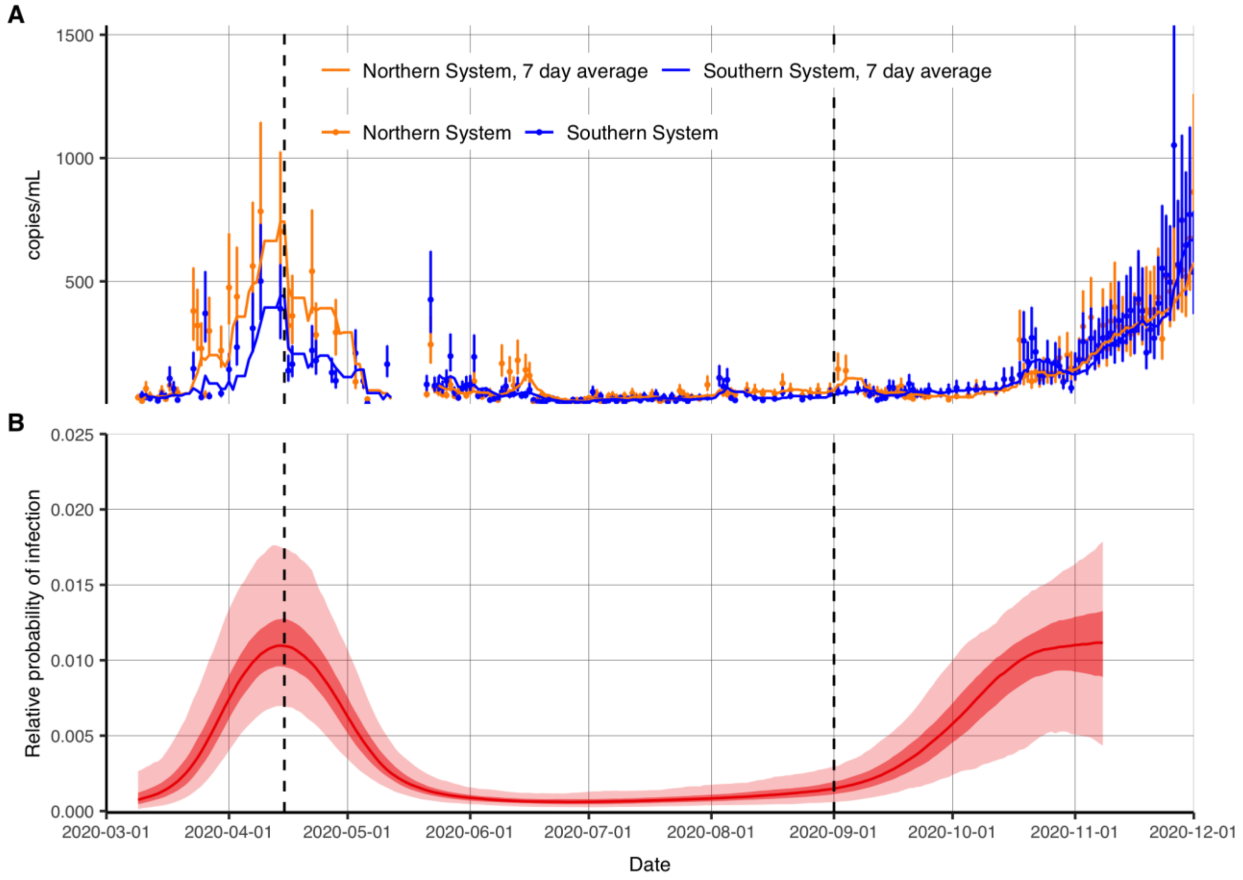

Fig. S19. Epidemic trajectory inferred using cycle threshold (Ct) distributions from routine testing in Brigham & Women's Hospital in Massachusetts tracks viral loads in Massachusetts wastewater samples. (**A**) SARS-CoV-2 viral RNA signal in Massachusetts wastewater over time (*35*). (**B**) As in Fig. 4, posterior distribution of scaled probability of infection by date from a Gaussian process (GP) model fit to all observed Ct values (ribbons show 95% and 50% credible intervals, line shows posterior median). Note that this is the same model fit as in Fig. 4, but the time axis extended back to March 1, 2020.

Parameter	Description	Prior point estimate	Prior
**Viral kinetics and Ct value model**			
$t_{eclipse}$	Time from infection to initial viral growth	0.00 days	Fixed
C_{zero}	Ct value at time of infection	40.0	Fixed
t_{peak}	Time from initial viral growth to peak viral load	5.00 days	Fixed
C_{peak}	Modal Ct value at peak viral load	20.6 (LTCF) or 19.7 (BWH)	Normal(20.6, 2.00) or Normal(19.7, 2.00)
t_{switch}	Time from peak viral load to secondary waning phase	9.38 (LTCF) or 13.3 days (BWH)	Normal(9.38, 3.00) or Normal(13.3, 3.00)
C_{switch}	Modal Ct value at $a = t_{eclipse} + t_{peak} + t_{switch}$	33.0 (LTCF) or 38.0 (BWH)	Normal(33.0, 1.00) or Fixed
t_{LOD}	Time from infection until modal Ct value is equal to the limit of detection	Inf (plateau)	Fixed
p_{addl}	Daily probability of detectability loss after t_{switch}	0.110 (LTCF) or 0.103 (BWH)	Beta(11.9, 95.9) or Beta(10.5, 91.2)
VL_{LOD}	Limit of detection of viral load ($\log_{10}$ RNA copies / mL)	3.00	Fixed
C_{LOD}	Limit of detection of Ct value	40.0	Fixed
σ_{obs}	Initial scale parameter for the Gumbel distribution until $a=t_{eclipse}+t_{peak}+t_{switch}$	5.15 (LTCF) or 5.00 (BWH)	Normal(5.15, 0.50) or Normal(5.00, 0.50)
s_{mod}	Multiplicative factor applied to scale parameter for the Gumbel distribution starting at $a = t_{eclipse}+t_{peak}+t_{switch}+t_{scale}$	0.400 (LTCF) or 0.789 (BWH)	Fixed
t_{mod}	Time from secondary waning phase until Gumbel distribution reaches its minimum scale parameter	14.0 days	Fixed

**SEEIRR and SEIR models**			
R_0	Basic reproductive number	Estimated (4 locations)	Log-normal(log(2.00), 0.60) and bound between (1.00, 10.0) for the SEEIRR model; Uniform(1.00, 10.0) in all SEIR models
t_0	Effective seed time	Estimated (4 locations)	Uniform(2020-03-01, 2020-05-11)
I_0	Proportion infected at seed time	0.002 (1 in 500)	Fixed
$1/\sigma'$	Pre-detectable latent period	2.00	Log-normal(log(2.00), 0.30)
$1/\alpha$	Pre-infectious incubation period	2.00	Log-normal(log(2.00), 0.30)
$1/\gamma'$	Infectious period (SEEIRR)	4.00	Log-normal(log(4.00), 0.60)
$1/w$	Post-infectious detectable period (SEEIRR)	11.0	Log-normal(log(11.0), 0.30)
$1/\sigma$	Incubation period (SEIR)	4.00	Log-normal(log(4.00), 0.25)
$1/\gamma$	Infectious period (SEIR)	4.00	Log-normal(log(4.00), 0.50)
**GP model**			
ν	Maximum covariance between two time points	1.50	Fixed
ρ	Rate of decline of the covariance between two time points as distance increases	0.03	Fixed

Table S1. Parameters used the viral load and cycle threshold (Ct) value distribution, the susceptible-exposed-infectious-recovered (SEIR) transmission model, the SEEIRR transmission model, and the Gaussian process (GP) model. Note that different priors for the viral kinetics parameters are assumed for the Massachusetts Long-Term Care Facilities (LTCF) or Brigham & Women's Hospital (BWH) data.

Movie S1. Multiple cross-sections of cycle threshold (Ct) values can be combined to improve the estimation of the epidemic trajectory over time. Animation of epidemic trajectory estimation (**Bottom**) using the Gaussian process GP model fit repeatedly to weekly samples of observed cycle threshold (Ct) value data (**Top**) in an ongoing simulated epidemic. 2000 samples were randomly taken from the population each week. The red line indicates the true daily per capita incidence of the simulated data. The blue line and ribbons show the posterior median, 50% (dark blue) and 95% (light blue) credible intervals for the estimated daily per capita incidence curve. Dashed vertical lines show time of sample collection. Incidence was estimated back to 35 days prior to the first sample time.

Movie S2. Estimated epidemic trajectories using multiple cross-sections of cycle threshold (Ct) values at different weekly sample sizes. Using the same simulation and Gaussian process (GP) model as in Movie S1, the animation shows the results estimating the underlying per capita incidence curve each week using no data (prior only), 50, 200, 500, 1000 or 2000 Ct values (including negative samples) obtained each week through randomly sampling the population. Dashed vertical lines show time of sample collection. The blue line and ribbons show the posterior median, 50% (dark blue) and 95% (light blue) credible intervals for the estimated daily per capita incidence curve. Incidence was estimated back to 35 days prior to the first sample time.

Movie S3. Multiple cross-sections of cycle threshold (Ct) values can estimate the underlying incidence curve when sampling is initiated partway through the epidemic. Using the same simulation, sampling scheme and Gaussian process (GP) model as in Movie S1, but with sampling commencing part way through the epidemic. (**Top**) cycle threshold (Ct) data used for the simulation. (**Bottom**) the red line indicates the true daily per capita incidence of the simulated data. The blue line and ribbons show the posterior median, 50% (dark blue) and 95% (light blue) credible intervals for the estimated daily per capita incidence curve. Dashed vertical lines show time of sample collection. Incidence was estimated back to 35 days prior to the first sample time.

Data S1. Ct values and collection dates for samples obtained from four long-term care facilities in Massachusetts. Variables included are anonymized facility identifier, collection date and week, RT-qPCR result, anonymized unique identifier, and RP (control), N2 and N1 Ct values.

Data S2. ORF1ab Ct values and collection dates for samples obtained from the Brigham & Women's Hospital, Massachusetts.

References and Notes

1. H. V. Fineberg, M. E. Wilson, Epidemic science in real time. *Science* **324**, 987 (2009). doi:10.1126/science.1176297 Medline

2. World Health Organization, Public health surveillance for COVID-19: Interim guidance (2020); www.who.int/publications/i/item/who-2019-nCoV-surveillanceguidance-2020.8.

3. T. Jombart, K. van Zandvoort, T. W. Russell, C. I. Jarvis, A. Gimma, S. Abbott, S. Clifford, S. Funk, H. Gibbs, Y. Liu, C. A. B. Pearson, N. I. Bosse, Centre for the Mathematical Modelling of Infectious Diseases COVID-19 Working Group, R. M. Eggo, A. J. Kucharski, W. J. Edmunds, Inferring the number of COVID-19 cases from recently reported deaths. *Wellcome Open Res.* **5**, 78 (2020). doi:10.12688/wellcomeopenres.15786.1

4. M. Lipsitch, D. L. Swerdlow, L. Finelli, Defining the epidemiology of COVID-19—Studies needed. *N. Engl. J. Med.* **382**, 1194–1196 (2020). doi:10.1056/NEJMp2002125 Medline

5. R. A. Betensky, Y. Feng, Accounting for incomplete testing in the estimation of epidemic parameters. *Int. J. Epidemiol.* **49**, 1419–1426 (2020). doi:10.1093/ije/dyaa116 Medline

6. J. L. Vaerman, P. Saussoy, I. Ingargiola, Evaluation of real-time PCR data. *J. Biol. Regul. Homeost. Agents* **18**, 212–214 (2004). Medline

7. M. R. Tom, M. J. Mina, To interpret the SARS-CoV-2 test, consider the cycle threshold value. *Clin. Infect. Dis.* **71**, 2252–2254 (2020). doi:10.1093/cid/ciaa619 Medline

8. T. C. Quinn, M. J. Wawer, N. Sewankambo, D. Serwadda, C. Li, F. Wabwire-Mangen, M. O. Meehan, T. Lutalo, R. H. Gray, Rakai Project Study Group, Viral load and heterosexual transmission of human immunodeficiency virus type 1. *N. Engl. J. Med.* **342**, 921–929 (2000). doi:10.1056/NEJM200003303421303 Medline

9. J. A. Fuller, M. K. Njenga, G. Bigogo, B. Aura, M. O. Ope, L. Nderitu, L. Wakhule, D. D. Erdman, R. F. Breiman, D. R. Feikin, Association of the C_T values of real-time PCR of viral upper respiratory tract infection with clinical severity, Kenya. *J. Med. Virol.* **85**, 924–932 (2013). doi:10.1002/jmv.23455 Medline

10. S. Bolotin, S. L. Deeks, A. Marchand-Austin, H. Rilkoff, V. Dang, R. Walton, A. Hashim, D. Farrell, N. S. Crowcroft, Correlation of Real Time PCR Cycle Threshold Cut-Off with *Bordetella pertussis* Clinical Severity. *PLOS ONE* **10**, e0133209 (2015). doi:10.1371/journal.pone.0133209 Medline

11. T. K. Tsang, B. J. Cowling, V. J. Fang, K. H. Chan, D. K. M. Ip, G. M. Leung, J. S. M. Peiris, S. Cauchemez, Influenza A virus shedding and infectivity in households. *J. Infect. Dis.* **212**, 1420–1428 (2015). doi:10.1093/infdis/jiv225 Medline

12. M. Moraz, D. Jacot, M. Papadimitriou-Olivgeris, L. Senn, G. Greub, K. Jaton, O. Opota, Universal admission screening strategy for COVID-19 highlighted the clinical importance of reporting SARS-CoV-2 viral loads. *New Microbes New Infect.* **38**, 100820 (2020). doi:10.1016/j.nmni.2020.100820 Medline

13. Y. Chen, L. Li, SARS-CoV-2: Virus dynamics and host response. *Lancet Infect. Dis.* **20**, 515–516 (2020). doi:10.1016/S1473-3099(20)30235-8 Medline

14. D. Jacot, G. Greub, K. Jaton, O. Opota, Viral load of SARS-CoV-2 across patients and compared to other respiratory viruses. *Microbes Infect.* **22**, 617–621 (2020). doi:10.1016/j.micinf.2020.08.004 Medline

15. K. A. Walsh, K. Jordan, B. Clyne, D. Rohde, L. Drummond, P. Byrne, S. Ahern, P. G. Carty, K. K. O'Brien, E. O'Murchu, M. O'Neill, S. M. Smith, M. Ryan, P. Harrington, SARS-CoV-2 detection, viral load and infectivity over the course of an infection. *J. Infect.* **81**, 357–371 (2020). doi:10.1016/j.jinf.2020.06.067 Medline

16. A. S. Walker, E. Pritchard, T. House, J. V. Robotham, P. J. Birrell, I. Bell, J. I. Bell, J. N. Newton, J. Farrar, I. Diamond, R. Studley, J. Hay, K.-D. Vihta, T. Peto, N. Stoesser, P. C. Matthews, D. W. Eyre, K. B. Pouwels, COVID-19 Infection Survey Team, Ct threshold values, a proxy for viral load in community SARS-CoV-2 cases, demonstrate wide variation across populations and over time. medRxiv 2020.10.25.20219048 [Preprint]. 4 April 2021. https://doi.org/10.1101/2020.10.25.20219048.

17. M. Gousseff, P. Penot, L. Gallay, D. Batisse, N. Benech, K. Bouiller, R. Collarino, A. Conrad, D. Slama, C. Joseph, A. Lemaignen, F.-X. Lescure, B. Levy, M. Mahevas, B. Pozzetto, N. Vignier, B. Wyplosz, D. Salmon, F. Goehringer, E. Botelho-Nevers, COCOREC study group, Clinical recurrences of COVID-19 symptoms after recovery: Viral relapse, reinfection or inflammatory rebound? *J. Infect.* **81**, 816–846 (2020). doi:10.1016/j.jinf.2020.06.073 Medline

18. G. Rydevik, G. T. Innocent, G. Marion, R. S. Davidson, P. C. L. White, C. Billinis, P. Barrow, P. P. C. Mertens, D. Gavier-Widén, M. R. Hutchings, Using combined diagnostic test results to hindcast trends of infection from cross-sectional data. *PLOS Comput. Biol.* **12**, e1004901 (2016). doi:10.1371/journal.pcbi.1004901 Medline

19. N. Keyfitz, *Applied Mathematical Demography* (Springer, ed. 2, 1985).

20. J. Wallinga, M. Lipsitch, How generation intervals shape the relationship between growth rates and reproductive numbers. *Proc. R. Soc. B.* **274**, 599–604 (2007). doi:10.1098/rspb.2006.3754 Medline

21. B. Borremans, N. Hens, P. Beutels, H. Leirs, J. Reijniers, Estimating time of infection using prior serological and individual information can greatly improve incidence estimation of human and wildlife infections. *PLOS Comput. Biol.* **12**, e1004882 (2016). doi:10.1371/journal.pcbi.1004882 Medline

22. H. Salje, D. A. T. Cummings, I. Rodriguez-Barraquer, L. C. Katzelnick, J. Lessler, C. Klungthong, B. Thaisomboonsuk, A. Nisalak, A. Weg, D. Ellison, L. Macareo, I. K. Yoon, R. Jarman, S. Thomas, A. L. Rothman, T. Endy, S. Cauchemez, Reconstruction of antibody dynamics and infection histories to evaluate dengue risk. *Nature* **557**, 719–723 (2018). doi:10.1038/s41586-018-0157-4 Medline

23. J. A. Hay, A. Minter, K. E. C. Ainslie, J. Lessler, B. Yang, D. A. T. Cummings, A. J. Kucharski, S. Riley, An open source tool to infer epidemiological and immunological dynamics from serological data: Serosolver. *PLOS Comput. Biol.* **16**, e1007840 (2020). doi:10.1371/journal.pcbi.1007840 Medline

24. H. E. de Melker, F. G. A. Versteegh, J. F. P. Schellekens, P. F. M. Teunis, M. Kretzschmar, The incidence of *Bordetella pertussis* infections estimated in the population from a

combination of serological surveys. *J. Infect.* **53**, 106–113 (2006). doi:10.1016/j.jinf.2005.10.020 Medline

25. J. Simonsen, K. Mølbak, G. Falkenhorst, K. A. Krogfelt, A. Linneberg, P. F. M. Teunis, Estimation of incidences of infectious diseases based on antibody measurements. *Stat. Med.* **28**, 1882–1895 (2009). doi:10.1002/sim.3592 Medline

26. P. F. M. Teunis, J. C. H. van Eijkeren, C. W. Ang, Y. T. H. P. van Duynhoven, J. B. Simonsen, M. A. Strid, W. van Pelt, Biomarker dynamics: Estimating infection rates from serological data. *Stat. Med.* **31**, 2240–2248 (2012). doi:10.1002/sim.5322 Medline

27. D. A. Helb, K. K. A. Tetteh, P. L. Felgner, J. Skinner, A. Hubbard, E. Arinaitwe, H. Mayanja-Kizza, I. Ssewanyana, M. R. Kamya, J. G. Beeson, J. Tappero, D. L. Smith, P. D. Crompton, P. J. Rosenthal, G. Dorsey, C. J. Drakeley, B. Greenhouse, Novel serologic biomarkers provide accurate estimates of recent Plasmodium falciparum exposure for individuals and communities. *Proc. Natl. Acad. Sci. U.S.A.* **112**, E4438–E4447 (2015). doi:10.1073/pnas.1501705112 Medline

28. K. M. Pepin, S. L. Kay, B. D. Golas, S. S. Shriner, A. T. Gilbert, R. S. Miller, A. L. Graham, S. Riley, P. C. Cross, M. D. Samuel, M. B. Hooten, J. A. Hoeting, J. O. Lloyd-Smith, C. T. Webb, M. G. Buhnerkempe, Inferring infection hazard in wildlife populations by linking data across individual and population scales. *Ecol. Lett.* **20**, 275–292 (2017). doi:10.1111/ele.12732 Medline

29. N. J. Lennon, R. P. Bhattacharyya, M. J. Mina, H. L. Rehm, D. T. Hung, S. Smole, A. Woolley, E. S. Lander, S. B. Gabriel, Comparison of viral levels in individuals with or without symptoms at time of COVID-19 testing among 32,480 residents and staff of nursing homes and assisted living facilities in Massachusetts. medRxiv 2020.07.20.20157792 [Preprint]. 26 July 2020. https://doi.org/10.1101/2020.07.20.20157792.

30. T. K. Tsang, P. Wu, Y. Lin, E. H. Y. Lau, G. M. Leung, B. J. Cowling, Effect of changing case definitions for COVID-19 on the epidemic curve and transmission parameters in mainland China: A modelling study. *Lancet Public Health* **5**, e289–e296 (2020). doi:10.1016/S2468-2667(20)30089-X Medline

31. S. Flaxman, S. Mishra, A. Gandy, H. J. T. Unwin, T. A. Mellan, H. Coupland, C. Whittaker, H. Zhu, T. Berah, J. W. Eaton, M. Monod, Imperial College COVID-19 Response Team, A. C. Ghani, C. A. Donnelly, S. Riley, M. A. C. Vollmer, N. M. Ferguson, L. C. Okell, S. Bhatt, Estimating the effects of non-pharmaceutical interventions on COVID-19 in Europe. *Nature* **584**, 257–261 (2020). doi:10.1038/s41586-020-2405-7 Medline

32. K. M. Gostic, L. McGough, E. B. Baskerville, S. Abbott, K. Joshi, C. Tedijanto, R. Kahn, R. Niehus, J. A. Hay, P. M. De Salazar, J. Hellewell, S. Meakin, J. D. Munday, N. I. Bosse, K. Sherrat, R. N. Thompson, L. F. White, J. S. Huisman, J. Scire, S. Bonhoeffer, T. Stadler, J. Wallinga, S. Funk, M. Lipsitch, S. Cobey, Practical considerations for measuring the effective reproductive number, R_t. *PLOS Comput. Biol.* **16**, e1008409 (2020). doi:10.1371/journal.pcbi.1008409 Medline

33. S. Abbott, J. Hellewell, R. N. Thompson, K. Sherratt, H. P. Gibbs, N. I. Bosse, J. D. Munday, S. Meakin, E. L. Doughty, J. Y. Chun, Y.-W. D. Chan, F. Finger, P. Campbell, A. Endo, C. A. B. Pearson, A. Gimma, T. Russell, CMMID COVID modelling group, S. Flasche, A. J. Kucharski, R. M. Eggo, S. Funk, Estimating the time-varying reproduction number

of SARS-CoV-2 using national and subnational case counts. *Wellcome Open Res.* **5**, 112 (2020). doi:10.12688/wellcomeopenres.16006.2

34. X. Xu, T. Kypraios, P. D. O'Neill, Bayesian non-parametric inference for stochastic epidemic models using Gaussian Processes. *Biostatistics* **17**, 619–633 (2016). doi:10.1093/biostatistics/kxw011 Medline

35. Massachusetts Water Resources Authority, Wastewater COVID-19 Tracking (2021); www.mwra.com/biobot/biobotdata.htm.

36. S. Riley, C. Atchison, D. Ashby, C. A. Donnelly, W. Barclay, G. S. Cooke, H. Ward, A. Darzi, P. Elliott, REACT study group, REal-time Assessment of Community Transmission (REACT) of SARS-CoV-2 virus: Study protocol. *Wellcome Open Res.* **5**, 200 (2021). doi:10.12688/wellcomeopenres.16228.2

37. M. U. G. Kraemer, S. V. Scarpino, V. Marivate, B. Gutierrez, B. Xu, G. Lee, J. B. Hawkins, C. Rivers, D. M. Pigott, R. Katz, J. S. Brownstein, Data curation during a pandemic and lessons learned from COVID-19. *Nat. Comput. Sci.* **1**, 9–10 (2021). doi:10.1038/s43588-020-00015-6

38. M. J. Mina, C. J. E. Metcalf, A. B. McDermott, D. C. Douek, J. Farrar, B. T. Grenfell, A Global lmmunological Observatory to meet a time of pandemics. *eLife* **9**, e58989 (2020). doi:10.7554/eLife.58989 Medline

39. M. Kidd, A. Richter, A. Best, N. Cumley, J. Mirza, B. Percival, M. Mayhew, O. Megram, F. Ashford, T. White, E. Moles-Garcia, L. Crawford, A. Bosworth, S. F. Atabani, T. Plant, A. McNally, S-Variant SARS-CoV-2 Lineage B1.1.7 Is Associated With Significantly Higher Viral Load in Samples Tested by TaqPath Polymerase Chain Reaction. *J. Infect. Dis.* **223**, 1666–1670 (2021). doi:10.1093/infdis/jiab082 Medline

40. S. M. Kissler, J. R. Fauver, C. Mack, C. G. Tai, M. I. Breban, A. E. Watkins, R. M. Samant, D. J. Anderson, D. D. Ho, N. D. Grubaugh, Y. H. Grad, Densely sampled viral trajectories suggest longer duration of acute infection with B.1.1.7 variant relative to non-B.1.1.7 SARS-CoV-2. medRxiv 2021.02.16.21251535 [Preprint]. 19 February 2021. https://doi.org/10.1101/2021.02.16.21251535.

41. N. R. Faria, T. A. Mellan, C. Whittaker, I. M. Claro, D. D. S. Candido, S. Mishra, M. A. E. Crispim, F. C. S. Sales, I. Hawryluk, J. T. McCrone, R. J. G. Hulswit, L. A. M. Franco, M. S. Ramundo, J. G. de Jesus, P. S. Andrade, T. M. Coletti, G. M. Ferreira, C. A. M. Silva, E. R. Manuli, R. H. M. Pereira, P. S. Peixoto, M. U. G. Kraemer, N. Gaburo Jr., C. D. C. Camilo, H. Hoeltgebaum, W. M. Souza, E. C. Rocha, L. M. de Souza, M. C. de Pinho, L. J. T. Araujo, F. S. V. Malta, A. B. de Lima, J. D. P. Silva, D. A. G. Zauli, A. C. S. Ferreira, R. P. Schnekenberg, D. J. Laydon, P. G. T. Walker, H. M. Schlüter, A. L. P. Dos Santos, M. S. Vidal, V. S. Del Caro, R. M. F. Filho, H. M. Dos Santos, R. S. Aguiar, J. L. Proença-Modena, B. Nelson, J. A. Hay, M. Monod, X. Miscouridou, H. Coupland, R. Sonabend, M. Vollmer, A. Gandy, C. A. Prete Jr., V. H. Nascimento, M. A. Suchard, T. A. Bowden, S. L. K. Pond, C. H. Wu, O. Ratmann, N. M. Ferguson, C. Dye, N. J. Loman, P. Lemey, A. Rambaut, N. A. Fraiji, M. D. P. S. S. Carvalho, O. G. Pybus, S. Flaxman, S. Bhatt, E. C. Sabino, Genomics and epidemiology of the P.1 SARS-CoV-2 lineage in Manaus, Brazil. *Science* **372**, 815–821 (2021). doi:10.1126/science.abh2644 Medline

42. R. Niehus, E. van Kleef, Y. Mo, A. Turlej-Rogacka, C. Lammens, Y. Carmeli, H. Goossens, E. Tacconelli, B. Carevic, L. Preotescu, S. Malhotra-Kumar, B. S. Cooper, Quantifying antibiotic impact on within-patient dynamics of extended-spectrum beta-lactamase resistance. *eLife* **9**, e49206 (2020). doi:10.7554/eLife.49206 Medline

43. D. Rhoads, D. R. Peaper, R. C. She, F. S. Nolte, C. M. Wojewoda, N. W. Anderson, B. S. Pritt, College of American Pathologists (CAP) Microbiology Committee perspective: Caution must be used in interpreting the cycle threshold (Ct) value. *Clin. Infect. Dis.* **72**, e685–e686 (2021). doi:10.1093/cid/ciaa1199 Medline

44. A. T. Xiao, Y. X. Tong, S. Zhang, Profile of RT-PCR for SARS-CoV-2: A preliminary study from 56 COVID-19 patients. *Clin. Infect. Dis.* **71**, 2249–2251 (2020). doi:10.1093/cid/ciaa460 Medline

45. W. C. Ko, J. M. Rolain, N. Y. Lee, P. L. Chen, C. T. Huang, P. I. Lee, P. R. Hsueh, Arguments in favour of remdesivir for treating SARS-CoV-2 infections. *Int. J. Antimicrob. Agents* **55**, 105933 (2020). doi:10.1016/j.ijantimicag.2020.105933 Medline

46. E. Mahase, Covid-19: What have we learnt about the new variant in the UK? *BMJ* **371**, m4944 (2020). doi:10.1136/bmj.m4944 Medline

47. D. J. Earl, M. W. Deem, Parallel tempering: Theory, applications, and new perspectives. *Phys. Chem. Chem. Phys.* **7**, 3910–3916 (2005). doi:10.1039/b509983h Medline

48. The New York Times, Coronavirus (Covid-19) Data in the United States, Github (2021); https://github.com/nytimes/covid-19-data.

49. D. B. Larremore, B. Wilder, E. Lester, S. Shehata, J. M. Burke, J. A. Hay, M. Tambe, M. J. Mina, R. Parker, Test sensitivity is secondary to frequency and turnaround time for COVID-19 screening. *Sci. Adv.* **7**, eabd5393 (2021). doi:10.1126/sciadv.abd5393 Medline

50. A. Tahamtan, A. Ardebili, Real-time RT-PCR in COVID-19 detection: Issues affecting the results. *Expert Rev. Mol. Diagn.* **20**, 453–454 (2020). doi:10.1080/14737159.2020.1757437 Medline

51. K. K. W. To, O. T. Y. Tsang, W. S. Leung, A. R. Tam, T. C. Wu, D. C. Lung, C. C. Y. Yip, J. P. Cai, J. M. C. Chan, T. S. H. Chik, D. P. L. Lau, C. Y. C. Choi, L. L. Chen, W. M. Chan, K. H. Chan, J. D. Ip, A. C. K. Ng, R. W. S. Poon, C. T. Luo, V. C.-C. Cheng, J. F.-W. Chan, I. F.-N. Hung, Z. Chen, H. Chen, K.-Y. Yuen, Temporal profiles of viral load in posterior oropharyngeal saliva samples and serum antibody responses during infection by SARS-CoV-2: An observational cohort study. *Lancet Infect. Dis.* **20**, 565–574 (2020). doi:10.1016/S1473-3099(20)30196-1 Medline

52. R. Wölfel, V. M. Corman, W. Guggemos, M. Seilmaier, S. Zange, M. A. Müller, D. Niemeyer, T. C. Jones, P. Vollmar, C. Rothe, M. Hoelscher, T. Bleicker, S. Brünink, J. Schneider, R. Ehmann, K. Zwirglmaier, C. Drosten, C. Wendtner, Virological assessment of hospitalized patients with COVID-2019. *Nature* **581**, 465–469 (2020). doi:10.1038/s41586-020-2196-x Medline

53. Q. X. Long, X. J. Tang, Q. L. Shi, Q. Li, H. J. Deng, J. Yuan, J. L. Hu, W. Xu, Y. Zhang, F. J. Lv, K. Su, F. Zhang, J. Gong, B. Wu, X. M. Liu, J. J. Li, J. F. Qiu, J. Chen, A. L. Huang, Clinical and immunological assessment of asymptomatic SARS-CoV-2 infections. *Nat. Med.* **26**, 1200–1204 (2020). doi:10.1038/s41591-020-0965-6 Medline

54. Y. Liu, L. M. Yan, L. Wan, T. X. Xiang, A. Le, J. M. Liu, M. Peiris, L. L. M. Poon, W. Zhang, Viral dynamics in mild and severe cases of COVID-19. *Lancet Infect. Dis.* **20**, 656–657 (2020). doi:10.1016/S1473-3099(20)30232-2 Medline

55. M. Plummer, N. Best, K. Cowles, K. Vines, CODA: Convergence diagnosis and output analysis for MCMC. *R News* **6**, 7–11 (2006).

56. J. A. Hay, L. Kennedy-Shaffer, jameshay218/virosolver_paper: Publication release, version v1.0.1, Zenodo (2021); http://doi.org/10.5281/zenodo.4776834.

57. J. A. Hay, L. Kennedy-Shaffer, jameshay218/virosolver: Publication release, version v1.0.2, Zenodo (2021); http://doi.org/10.5281/zenodo.4776812.

58. J. A. Hay, jameshay218/lazymcmc: virosolver paper release, version v1.0.2, Zenodo (2021); http://doi.org/10.5281/zenodo.4768739.

59. S. Abbott, J. Hickson, P. Ellis, J. D. Munday, epiforecasts/EpiNow2: First stable release, version v1.1.0, Zenodo (2020); http://doi.org/10.5281/zenodo.3994783.

60. R. FitzJohn, Odin: ODE generation and integration, R package version 1.0.8 (2020); https://CRAN.R-project.org/package=odin.

61. R. McElreath, *Statistical Rethinking: A Bayesian Course with Examples in R and STAN* (Chapman and Hall/CRC Press, ed. 2, 2020).

62. A. Chandrashekar, J. Liu, A. J. Martinot, K. McMahan, N. B. Mercado, L. Peter, L. H. Tostanoski, J. Yu, Z. Maliga, M. Nekorchuk, K. Busman-Sahay, M. Terry, L. M. Wrijil, S. Ducat, D. R. Martinez, C. Atyeo, S. Fischinger, J. S. Burke, M. D. Slein, L. Pessaint, A. Van Ry, J. Greenhouse, T. Taylor, K. Blade, A. Cook, B. Finneyfrock, R. Brown, E. Teow, J. Velasco, R. Zahn, F. Wegmann, P. Abbink, E. A. Bondzie, G. Dagotto, M. S. Gebre, X. He, C. Jacob-Dolan, N. Kordana, Z. Li, M. A. Lifton, S. H. Mahrokhian, L. F. Maxfield, R. Nityanandam, J. P. Nkolola, A. G. Schmidt, A. D. Miller, R. S. Baric, G. Alter, P. K. Sorger, J. D. Estes, H. Andersen, M. G. Lewis, D. H. Barouch, SARS-CoV-2 infection protects against rechallenge in rhesus macaques. *Science* **369**, 812–817 (2020). doi:10.1126/science.abc4776 Medline

63. J. Yu, L. H. Tostanoski, L. Peter, N. B. Mercado, K. McMahan, S. H. Mahrokhian, J. P. Nkolola, J. Liu, Z. Li, A. Chandrashekar, D. R. Martinez, C. Loos, C. Atyeo, S. Fischinger, J. S. Burke, M. D. Slein, Y. Chen, A. Zuiani, F. J. N. Lelis, M. Travers, S. Habibi, L. Pessaint, A. Van Ry, K. Blade, R. Brown, A. Cook, B. Finneyfrock, A. Dodson, E. Teow, J. Velasco, R. Zahn, F. Wegmann, E. A. Bondzie, G. Dagotto, M. S. Gebre, X. He, C. Jacob-Dolan, M. Kirilova, N. Kordana, Z. Lin, L. F. Maxfield, F. Nampanya, R. Nityanandam, J. D. Ventura, H. Wan, Y. Cai, B. Chen, A. G. Schmidt, D. R. Wesemann, R. S. Baric, G. Alter, H. Andersen, M. G. Lewis, D. H. Barouch, DNA vaccine protection against SARS-CoV-2 in rhesus macaques. *Science* **369**, 806–811 (2020). doi:10.1126/science.abc6284 Medline

64. S. F. Sia, L. M. Yan, A. W. H. Chin, K. Fung, K. T. Choy, A. Y. L. Wong, P. Kaewpreedee, R. A. P. M. Perera, L. L. M. Poon, J. M. Nicholls, M. Peiris, H. L. Yen, Pathogenesis and transmission of SARS-CoV-2 in golden hamsters. *Nature* **583**, 834–838 (2020). doi:10.1038/s41586-020-2342-5 Medline

65. A. M. Bosco-Lauth, A. E. Hartwig, S. M. Porter, P. W. Gordy, M. Nehring, A. D. Byas, S. VandeWoude, I. K. Ragan, R. M. Maison, R. A. Bowen, Experimental infection of

domestic dogs and cats with SARS-CoV-2: Pathogenesis, transmission, and response to reexposure in cats. *Proc. Natl. Acad. Sci. U.S.A.* **117**, 26382–26388 (2020). doi:10.1073/pnas.2013102117 Medline

66. M. M. Arons, K. M. Hatfield, S. C. Reddy, A. Kimball, A. James, J. R. Jacobs, J. Taylor, K. Spicer, A. C. Bardossy, L. P. Oakley, S. Tanwar, J. W. Dyal, J. Harney, Z. Chisty, J. M. Bell, M. Methner, P. Paul, C. M. Carlson, H. P. McLaughlin, N. Thornburg, S. Tong, A. Tamin, Y. Tao, A. Uehara, J. Harcourt, S. Clark, C. Brostrom-Smith, L. C. Page, M. Kay, J. Lewis, P. Montgomery, N. D. Stone, T. A. Clark, M. A. Honein, J. S. Duchin, J. A. Jernigan, Public Health–Seattle and King County and CDC COVID-19 Investigation Team, Presymptomatic SARS-CoV-2 infections and transmission in a skilled nursing facility. *N. Engl. J. Med.* **382**, 2081–2090 (2020). doi:10.1056/NEJMoa2008457 Medline

67. L. Ferretti, C. Wymant, M. Kendall, L. Zhao, A. Nurtay, L. Abeler-Dörner, M. Parker, D. Bonsall, C. Fraser, Quantifying SARS-CoV-2 transmission suggests epidemic control with digital contact tracing. *Science* **368**, eabb6936 (2020). doi:10.1126/science.abb6936 Medline

68. S. A. Lauer, K. H. Grantz, Q. Bi, F. K. Jones, Q. Zheng, H. R. Meredith, A. S. Azman, N. G. Reich, J. Lessler, The incubation period of coronavirus disease 2019 (COVID-19) from publicly reported confirmed cases: Estimation and application. *Ann. Intern. Med.* **172**, 577–582 (2020). doi:10.7326/M20-0504 Medline

69. X. He, E. H. Y. Lau, P. Wu, X. Deng, J. Wang, X. Hao, Y. C. Lau, J. Y. Wong, Y. Guan, X. Tan, X. Mo, Y. Chen, B. Liao, W. Chen, F. Hu, Q. Zhang, M. Zhong, Y. Wu, L. Zhao, F. Zhang, B. J. Cowling, F. Li, G. M. Leung, Temporal dynamics in viral shedding and transmissibility of COVID-19. *Nat. Med.* **26**, 672–675 (2020). doi:10.1038/s41591-020-0869-5 Medline

70. M. Cevik, M. Tate, O. Lloyd, A. E. Maraolo, J. Schafers, A. Ho, SARS-CoV-2, SARS-CoV, and MERS-CoV viral load dynamics, duration of viral shedding, and infectiousness: A systematic review and meta-analysis. *Lancet Microbe* **2**, e13–e22 (2021). doi:10.1016/S2666-5247(20)30172-5 Medline

71. B. Borremans, A. Gamble, K. C. Prager, S. K. Helman, A. M. McClain, C. Cox, V. Savage, J. O. Lloyd-Smith, Quantifying antibody kinetics and RNA detection during early-phase SARS-CoV-2 infection by time since symptom onset. *eLife* **9**, e60122 (2020). doi:10.7554/eLife.60122 Medline

72. A. Singanayagam, M. Patel, A. Charlett, J. Lopez Bernal, V. Saliba, J. Ellis, S. Ladhani, M. Zambon, R. Gopal, Duration of infectiousness and correlation with RT-PCR cycle threshold values in cases of COVID-19, England, January to May 2020. *Euro Surveill.* **25**, 2001483 (2020). doi:10.2807/1560-7917.ES.2020.25.32.2001483 Medline

73. B. La Scola, M. Le Bideau, J. Andreani, V. T. Hoang, C. Grimaldier, P. Colson, P. Gautret, D. Raoult, Viral RNA load as determined by cell culture as a management tool for discharge of SARS-CoV-2 patients from infectious disease wards. *Eur. J. Clin. Microbiol. Infect. Dis.* **39**, 1059–1061 (2020). doi:10.1007/s10096-020-03913-9 Medline

74. J. Bullard, K. Dust, D. Funk, J. E. Strong, D. Alexander, L. Garnett, C. Boodman, A. Bello, A. Hedley, Z. Schiffman, K. Doan, N. Bastien, Y. Li, P. G. Van Caeseele, G. Poliquin, Predicting Infectious Severe Acute Respiratory Syndrome Coronavirus 2 From

Diagnostic Samples. *Clin. Infect. Dis.* **71**, 2663–2666 (2020). doi:10.1093/cid/ciaa638 Medline

75. J. van Beek, Z. Igloi, T. Boelsums, E. Fanoy, H. Gotz, R. Molenkamp, J. van Kampen, C. GeurtsvanKessel, A. van der Eijk, D. van de Vijver, M. Koopmans, From more testing to smart testing: data-guided SARS-CoV-2 testing choices. medRxiv 2020.10.13.20211524 [Preprint]. 14 October 2020. https://doi.org/10.1101/2020.10.13.20211524.

76. J. Y. Kim, J. H. Ko, Y. Kim, Y. J. Kim, J. M. Kim, Y. S. Chung, H. M. Kim, M. G. Han, S. Y. Kim, B. S. Chin, Viral load kinetics of SARS-CoV-2 infection in first two patients in Korea. *J. Korean Med. Sci.* **35**, e86 (2020). doi:10.3346/jkms.2020.35.e86 Medline

77. N. Li, X. Wang, T. Lv, Prolonged SARS-CoV-2 RNA shedding: Not a rare phenomenon. *J. Med. Virol.* **92**, 2286–2287 (2020). doi:10.1002/jmv.25952 Medline

78. H. Kawasuji, Y. Takegoshi, M. Kaneda, A. Ueno, Y. Miyajima, K. Kawago, Y. Fukui, Y. Yoshida, M. Kimura, H. Yamada, I. Sakamaki, H. Tani, Y. Morinaga, Y. Yamamoto, Viral load dynamics in transmissible symptomatic patients with COVID-19. medRxiv 2020.06.02.20120014 [Preprint]. 4 June 2020. https://doi.org/10.1101/2020.06.02.20120014.

79. J. Hellewell, T. W. Russell, SAFER Investigators and Field Study Team, Crick COVID-19 Consortium, CMMID COVID-19 working group, R. Beale, G. Kelly, C. Houlihan, E. Nastouli, A. J. Kucharski, Estimating the effectiveness of routine asymptomatic PCR testing at different frequencies for the detection of SARS-CoV-2 infections. *BMC Med.* **19**, 106 (2021). doi:10.1186/s12916-021-01982-x Medline
